# White spot syndrome viral protein VP9 alters the cellular higher‐order chromatin structure

**DOI:** 10.1096/fba.2019-00086

**Published:** 2020-03-17

**Authors:** Xi Tan, Andrea Ravasio, Hui T. Ong, Jinlu Wu, Choy L. Hew

**Affiliations:** ^1^ Mechanobiology Institute National University of Singapore Singapore Singapore; ^2^ Institute for Biological and Medical Engineering Schools of Engineering, Medicine and Biological Sciences Pontificia Universidad Católica de Chile Santiago de Chile Chile; ^3^ Department of Biological Sciences National University of Singapore Singapore Singapore; ^4^ Present address: School of Basic Medical Sciences Guizhou University of Traditional Chinese Medicine Guiyang Guizhou Province China

**Keywords:** chromatin, DNA mimic, histones, ICP11, VP9, white spot syndrome virus, WSSV

## Abstract

Viral protein 9 (VP9) is a non‐structural protein of white spot syndrome virus (WSSV) highly expressed during the early stage of infection. The crystal structure of VP9 suggests that the polymers of VP9 dimers resemble a DNA mimic, but its function remains elusive. In this study, we demonstrated that VP9 impedes histones binding to DNA via single‐molecule manipulation. We established VP9 expression in HeLa cells due to the lack of a WSSV‐susceptible cell line, and observed abundant VP9 in the nucleus, which mirrors its distribution in the hemocytes of WSSV‐infected shrimp. VP9 expression increased the dynamics and rotational mobility of histones in stable H3‐GFP HeLa cells as revealed by fluorescent recovery after photobleaching and fluorescence anisotropy imaging, which suggested a loosened compaction of chromatin structure. Successive salt fractionation showed that a prominent population of histones was solubilized in high salt concentrations, which implies alterations of bulk chromatin structure. Southern blotting identified that VP9 alters juxtacentromeric chromatin structures to be more accessible to micrococcal nuclease digestion. RNA microarray revealed that VP9 expression also leads to significant changes of cellular gene expression. Our findings provide evidence that VP9 alters the cellular higher‐order chromatin structure, uncovering a potential strategy adopted by WSSV to facilitate its replication.

**Abbreviations**
BRWD1: bromodomain and WD (Trp‐Asp) repeat domain containing 1
BSA: bovine serum albumin
CHX: cycloheximide
FACS: fluorescence‐activated cell sorting
FAI: fluorescence anisotropy imaging
FRAP: fluoresecence recovery after photobleaching
FRET: fluorescence resonance energy transfer
GO: gene ontology
HSV: herpes simplex virus
ICP11: infected cell protein 11
IFI6: interferon‐induced protein 6
IP: isoelectric point
MNase: micrococcal nuclease
MX1: myxovirus resistance 1
NAP1: nucleosome assembly protein 1
NMR: nuclear magnetic resonace
TADs: topological associate domains
VP9: viral protein 9
WSSV: white spot syndrome virus
ZNF326: zinc finger 326

## 1 INTRODUCTION

White spot syndrome virus (WSSV) is one of the most virulent and prevalent shrimp viral pathogens.^1^, ^2^ It is a large dsDNA virus with a genome of ~300 kb and approximately 180 ORFs,^3^, ^4^ being the only member of the genus *Whispovirus* in *Nimaviridae* family*.* The viral morphogenesis mainly occurs within virogenic stroma in the nucleus.^5^ So far, more than 50 viral structural and non‐structural proteins have been identified^6^, ^7^, ^8^, but three‐dimensional (3D) structural information is available for only a few proteins.^9^, ^10^, ^11^ The majority of their functions remain elusive.

Viral protein 9 (also named infected cell protein 11 [ICP11]), a non‐structural protein encoded by the WSSV *wsv230* gene, is highly expressed in both mRNA and protein levels during the early stage of viral infection.^9^, ^12^ The 3D structure of monomeric VP9 has been determined by both X‐ray crystallography and nuclear magnetic resonace (NMR), which revealed a ferredoxin fold with divalent metal ion binding sites.^9^ Subsequently, VP9 polymers were identified to function as a DNA mimic from the crystal structure.^13^ VP9 was found to co‐localize with histone H3 and H2A in both the nucleus and cytoplasm of hemocytes of WSSV‐infected shrimp. It was suggested that VP9 forms complexes with histone core proteins (H2A and H3 in particular) and may disrupt nucleosome assembly,^13^ but the detail of how VP9 could affect nucleosome and chromatin structure remains largely unknown. In addition, it is still unknown whether VP9 functions as monomer and/or polymer in a cell.

Viral morphogenesis studies indicate that viral replication in the host nucleus requires changes in host chromatin packaging, during which histones display significantly increased mobility and undergo relocation.^14^, ^15^, ^16^ On the other hand, viral genome structure is also affected by host cellular factors. For herpes simplex virus (HSV), the viral genomes are re‐organized into chromatin structure from a non‐nucleosomal structure upon transport to the nucleus, and the delicate balance between heterochromatin and euchromatin governs its lytic and latent life cycle.^17^, ^18^, ^19^ Recent reports showed that modulation of heterochromatin deposition is required for viral immediate early (IE) gene expression of HSV. Defects in modulation of the heterochromatin deposition lead to an increase of H3K9me3 at the IE transcript promoter, which causes the repression of IE gene transcription and is responsible for establishing latency. In the late infection stage, the cellular interchromosomal space is progressively expanded, resulting in host chromatin margination.^20^, ^21^ Many nuclear viruses, including WSSV, induce the accumulation of the host chromatin to the nuclear periphery, leaving the interchromosomal space available for viral particle assembly.^22^, ^23^ Although this gross alteration of chromatin architecture is commonly observed in different nuclear viral infections, only a few viral proteins have been identified or proposed to be involved in the regulation of nuclear architecture.^24^, ^25^

To further explore whether VP9 could potentially affect host chromatin organization, it would be ideal to conduct experiments in cells derived from their preferred host, the shrimp. However, there is a lack of well‐established WSSV‐susceptible shrimp cell lines. In the context of the chromatin organization, the canonical nucleosome assembly, nucleosome‐based condensation and high degree of the folding of chromatin structure share the formulation of dogmas, which tightly governs the concerted chromatin structural formation among eukaryotes, ranging from metazoan to human.^26^, ^27^, ^28^, ^29^, ^30^ The spatial organization of chromatin, as evolutionarily conserved topologically associating domains (TADs), plays povital roles in essential genome functions,^31^, ^32^, ^33^ which is thought to be driven by the TAD self‐assembly corresponded to the ability of charge‐based nucleosomal aggregation in Drosophila cells.^34^ Our study pointed to the role of VP9 for TADs, more specifically, in alteration of the extent of their homogeneity of compaction, dynamic aspects, and accessibility to complexes required for epigenetic regulations. The classical hierarchy of TADs across different species imprints with conserved chromatin‐chromatin contact, and VP9‐histone interactions were evident in HeLa cells. Considering other available tools, such as antibodies, H3‐GFP and H2B‐GFP stable HeLa cell lines, we carried out our experiments in Hela cells.

We first set out to establish VP9 expression in HeLa cells, and found that the expressed VP9 displays a similar distribution inside HeLa cells as in hemocytes of WSSV‐infected shrimp via immunostaining. Therefore, we performed in vitro single molecule manipulation, fluorescence recovery after photobleaching (FRAP), fluorescence anisotropy imaging (FAI), and salt fractionation experiments using HeLa cells to investigate whether VP9 was able to affect DNA‐histone interactions, chromatin packaging heterogeneity and alteration of chromatin structure. Our findings indicate that VP9 modulates the higher‐order structure of chromatin, especially in heterochromatin regions, but VP9 expression alone is unable to unfold primary chromatin structures or disrupt nucleosome assembly.

## 2 METHODS

### 2.1 Cell culture and transfection

HeLa cells were maintained in enriched Dulbecco's Modified Eagles's Medium (DMEM). 1.3 × 10^4^ cells were seeded in a 35 mm IWAKI glass bottom dish coated with fibronectin (Sigma) and maintained for 12‐16 hours before transfection. The medium was replaced with 2 mL of fresh pre‐warmed medium before the transfection solution was added. For live cell imaging, the transfection mix was composed of Lipofectamine^®^ 3000 (Invitrogen), 0.4 µg of pXJ40‐VP9, and 0.1 µg of pXJ40‐mCherry plasmids. The expression of mCherry proteins was used as an indicator to optimize transfection conditions to achieve at least 70% transfection efficiency. For immunostaining, the transfection was carried out by Lipofectamine^®^ 3000 (Invitrogen) using the same conditions, but only 0.4 µg of pXJ40‐VP9 was added. Control transfections were carried out with 0.4 µg of pXJ40 empty vector and 0.1 µg of pXJ40‐mCherry plasmid.

### 2.2 VP9 expression in HeLa cells and distribution in time course

Viral protein 9‐transfected cells were fixed with 4% formaldehyde and permeabilized with 0.3% Triton X‐100. The cells were then blocked with 5% bovine serum albumin (BSA), followed with incubation with the mouse monoclonal VP9 antibody.^9^ After washing with PBS, Alexa Fluor‐conjugated secondary antibody (1:500 dilution) was added and incubated for 1 hour at RT. After further washing, the cells were imaged with a Nikon A1Rsi confocal microscope using optimized laser power and scanning speed to avoid over saturation and bleaching. To quantify the relative intensity of VP9 expression, a custom code written in MATLAB was used. The average intensity of VP9 in the nucleus was calculated from the total value of each pixel within the nucleus divided by the nuclear area. The average intensity of VP9 in the cytoplasm was calculated by subtracting the nuclear VP9 intensity from the total VP9 intensity of the cell, and this value was then divided by the cytoplasmic area. The ratio of VP9 intensity in the nucleus *vs* cytoplasm was calculated by dividing the average nuclear intensity with the average cytoplasm intensity. Statistical analysis was carried out by ANOVA with Dunnett's post‐test. VP9 intensity at 9 hpt was used as a baseline for comparison of VP9 expression at later stages in order to assess significant changes. Rabbit polyclonal anti‐histone H3 and anti‐histone modification H3K9me3 antibodies were purchased from Abcam and Millipore, respectively. DAPI (Invitrogen) was used for nuclear staining. All secondary antibodies conjugated with Fluor dyes (405, 488, 561 nm) were purchased from Biotium, Inc.

### 2.3 Single‐molecule manipulation using Transverse Magnetic Tweezers

This method manipulates the folding/unfolding of a single copy of dsDNA around histone octamers by external force. The stepwise folding/unfolding is a unique feature of typical nucleosome assembly and disassembly.^35^, ^36^ When the external force is below a threshold level, histone‐DNA interaction favors nucleosome formation in the presence of histone chaperones. When the force is increased above the threshold, the DNA around octamers is stretched and unfolded.^37^ The experimental set up was adapted from Lim et al^38^ Briefly, No. 0 coverslips were polished and coated with streptavidin to allow specific tethering of a single DNA molecule, which was labeled with biotin at both ends by a polymerase reaction.^39^ The polished edge of the coverslip was placed at the center of a flow chamber in order to distinguish the polished edge where the DNA tethers reside. For these experiments, commercial λ‐DNA (48 502 bp), was used (Invitrogen, Life Technology). A low concentration (5 ng/μL) of the Biotin‐λ DNA‐Biotin mixture was added into the flow chamber and incubated for 10 minutes. The remaining unbound Biotin‐λ DNA‐Biotin was washed away with PBS buffer before adding 2.8‐μm‐sized streptavidin‐coated magnetic beads (Dynabeads M‐280 Streptavidin, Life Technology), followed by incubation for another 10 minutes to form a single tether with DNA. The DNA tether was manipulated using a permanent magnet (5 mm diameter) to impose a magnetic force on the bead. This magnet was brought close to the flow chamber in parallel geometry with the DNA tether. A long working distance 60X microscope objective lens was used to image the DNA tether onto a CCD camera (Pike F‐032, Allied Vision Technologies, Germany), which enabled recording of images at 100 frames per second. DNA extension was measured and recorded in real‐time using a program written by LabVIEW (National Instruments, US). The sub‐pixel localization of the magnetic bead was obtained using the centroid tracking method, and subtracted with the coverslip edge to obtain the DNA extension.^40^ The applied magnetic force was calculated using the following equation: $F=\frac{\kappa_{B}TZ}{〈\delta X^{2}〉}$, where *κ*_B_ is the Boltzmann constant, *T* is temperature, *Z* is the DNA extension, and <*δX* ^2^> is the transverse fluctuation along a direction perpendicular to the force direction. The force applied on the DNA tether can be modulated by changing the distance between the magnet and magnet bead, which was controlled by the LabVIEW program. The force‐extension curve of the DNA tether was obtained by measuring the DNA tether extension at various magnet positions which created various pulling forces. The constant pulling force was gradually reduced from 6.13 to 0.19 pN over 1000 seconds, and then recovered to the original 6.13 pN. At each constant pulling force, the DNA extension was recorded for about 70‐100 seconds. VP9 or BSA was pre‐incubated with histone octamer at a specified molar ratio (ie, The 1:100 ratio is composed of 10 nmol/L histone octamer with 10 mmol/L VP9) and allowed to incubate for 30 minutes at room temperature. The protein mixture was then added into the flow chamber.

### 2.4 Fluorescence recovery after photobleaching assay

Fluorescence recovery after photobleaching is a well‐established technique used to characterize the mobility of cellular molecules.^41^ It has been applied to document the intrinsic properties of histone kinetics and to quantify the proportions of histones with rapid, slow, and very slow mobility.^42^ Histones with rapid mobility refers to the unbound histone (free histone), which usually comprises approximately 5 to 10% of the total histone proteins.^42^, ^43^ These diffuse across the bleached spot easily and the bleached spot recovers rapidly. Histones with low mobility usually represent the histones in euchromatin, which can undergo exchange from their binding sites although the bleached spot recovered slowly. The histones with very slow mobility are hardly exchanged from their binding sites, and these represent highly compacted chromatin such as heterochromatin.

H3‐GFP and H2B‐GFP stable HeLa cell lines were a gift from H. Kimura (Osaka University). The microscopy (Nikon A1Rsi Confocal Microscope) set up and image collection protocol were adapted from H. Kimura.^42^ Briefly, control and VP9‐transfected H3‐GFP and H2B‐GFP stable cells were grown in petri dishes for 22 hours. A 1.9 × 1.9 μm^2^ area in the nucleus of a single cell was then bleached once with 100% laser power. To gain the full stack of focal planes during recoveries, the pinhole of 488 nm was adjusted to 255.4 nm and the Galvano scanning mode was used. Images were collected over a period of 558 minutes at the following rates and durations: 4 frames in every 15 seconds in the beginning; followed by 7 frames in every 1 minutes; 7 frames in every 10 minutes; 16 frames in every 30 minutes.

The average intensity values of the bleached ROI (Region of interest) and background region were measured using Fiji software while the average intensity per pixel of each nucleus varied in time was determined using Imaris (version 7.7.1). The values for each time point were normalized using the double normalization method.^44^ The normalized values were fitted with a double exponential (exp) function: $\text{In}=P1\left(1-\exp^{-k_{1}t}\right)+P2\left(1-\exp^{-k_{2}t}\right)$, where In = Relative Intensity, *P* = Plateau value, *K* = association constant, and *t* = time. The half‐life for each population was computed using equation: $t_{1/2}=\frac{\ln2}{K}$. Curves were analyzed using GraphPad Prism (version 6.0). The errors were presented as 95% confidence intervals. The difference between two FRAP curves (control and VP9‐transfected) can be distinguished by the recovery rate and the location of the plateau. At the same concentration of the fluorescent molecule of interest, a higher recovery rate represents higher mobility, whereas the plateau reflects the existence of different binding states and the plateau height reflects the proportion of the molecular fractions occupying each binding state.^41^ In order to minimize the effects of VP9 expression on histone abundance, we carried out an experiment in parallel with cycloheximide (CHX, Sigma) in order to inhibit protein synthesis. 10 ug/ml CHX was added to the cell culture medium 1 hour before bleaching.

### 2.5 Fluorescent anisotropy assay

Fluorescence anisotropy imaging was performed using the anisotropy module on a NikonA1R inverted microscope.^45^ In brief, control (mCherry only) and mCherry‐VP9‐transfected H3‐GFP stable cells were grown in petri dishes for 22 hours. Two images of the H3‐EGFP nuclei were obtained in parallel and cross polarization emission channels. Anisotropy was computed from these images after background subtraction and 3 × 3 image smoothening using a custom‐written program in MATLAB. Anisotropy was calculated as $r=\frac{I_{\text{para}}-g\times I_{\text{cross}}}{I_{\text{para}}+2g\times I_{\text{cross}}}$, where $I_{\text{para}}$ and $I_{\text{cross}}$ are the intensities in the parallel and cross polarization channels, respectively, and *g* represents the *g*‐factor, the ratio of sensitivity of experimental setup for parallel and cross polarizations. The *g*‐factor is calculated using fluorescein isothiocyanate solution with a known anisotropy of 0.02. If fluorophore molecules are at fluorescence resonance energy transfer (FRET) distance, homo‐FRET will cause depolarization of the emission, even if the rotational mobility is low. In such case, the measured anisotropy value does not directly reflect the rotational mobility. However, photobleaching experiments revealed that H3‐EGFP anisotropy did not change in both control and VP9‐transfected cells, confirming that homo‐FRET does not contribute to the anisotropy measured in our experiments (Figure S6B). Thus, the low value of anisotropy represents a high rotational mobility of H3, indicating the high fluidity (low compaction) of chromatin^45^, ^46^ (Figure 3A).

To exclude the possibility of non‐specific effects of VP9, we designed another control in which a plasmid containing GFP‐NLS (nuclear localization sequence) was co‐transfected with pXJ40‐VP9. GFP‐NSL is a recombinant protein that does not interact with DNA and histones.^47^ It is used as a standard negative control. We measured the anisotropy of GFP‐NLS in both VP9‐expressed cells and non‐VP9‐expressed cells.

### 2.6 Salt fractionation

The protocol of nuclei isolation was adapted from Caille's method.^48^ Briefly, after 24 hours transfection with VP9 plasmids, HeLa cells in a 150 mm dish were rinsed twice with ice‐cold PBS and then treated with ice‐chilled citric acid solution, which was composed of 0.01% Igepal CA‐630 (octylphenoxyl polyethoxyethanol, Sigma), and 1% citric acid solution (Sigma) in nuclease‐free water supplemented with 1 mmol/L β‐mercaptoethanol (Biolab), 1 mmol/L CaCl_2_, 1 mmol/L MgCl_2_ and EDTA‐free protease inhibitor (AEBSF 1 mmol/L, Aprotinin 800 nmol/L, Bestatin 50 umol/L, E64 15 umol/L, Leupeptin 20 umol/L, Pepstatin A 10 umol/L, Thermo Scientific Research). Nuclei were released whereas the cytomatrix remained adherent on the dish. The solution was harvested and centrifuged, and the resulting pellet (nuclei) was resuspended in PBS supplemented with EDTA‐free protease inhibitor, 1 mmol/L CaCl_2_ and 1 mmol/L MgCl_2._

The salt fractionation was carried out according to a previous study^49^ with modifications. For non‐successive fractionation, seven aliquots of nuclei were pelleted, and six of them were resuspended in parallel in a series of cold TM buffers (10 mmol/L Tris·HCl pH 7.4, 0.2% Triton X‐100, 1 mmol/L CaCl_2_ and 1 mmol/L MgCl_2_, EDTA‐free protease inhibitor) containing six different NaCl concentrations (0, 0.3, 0.6, 1, 1.5, and 2 mol/L). The remaining pellet that was not salt extracted was used to measure the total amount of proteins for calibration (both soluble and insoluble proteins). The suspended nuclei were rotated over‐night at 4°C and then pelleted for 1 hour at 13 000 *g*. The supernatant from each aliquot after centrifugation was stored at −20°C for SDS‐PAGE analysis. For successive fractionation, one nuclei preparation was successively extracted with the previously described buffers. After each extraction, the nuclei was pelleted down at 5000 *g* for 30 minutes at 4°C for the subsequent extraction, while the supernatant was collected, respectively, for SDS‐PAGE analysis. The bands containing histone proteins were further verified by mass spectrometry identification.^50^ The intensities of protein bands were quantified by ImageJ software.

### 2.7 Micrococcal nuclease accessibility assay

Control and VP9‐transfected cells were cultured on 100 mm petri dishes for 24 hours and permeabilized with a solution containing 100 μg/ml lysolecithin, 150 mmol/L sucrose, 80 mmol/L KCl, 35 mmol/L HEPES, pH 7.4, 5 mmol/L K_2_HPO_4_, 5 mmol/L MgCl, and 0.5 mmol/L CaCl_2_ at RT for 2.5 minutes.^51^ The cells were then treated with different concentrations of micrococcal nuclease (MNase) (0, 24, 48, and 96 U, Thermo Scientific) in 3 mL of buffer containing 150 mmol/L sucrose, 50 mmol/L Tris·HCl, pH 7.5, 50 mmol/L NaCl, 2 mmol/L CaCl_2_ for 20 minutes at RT. After lysis with 2.8 mL of 2 × TNESK solution (20 mmol/L Tris·HCl, pH 7.4, 0.2 mol/L NaCl, 2 mmol/L EDTA, 2% SDS, 5 μg/mL RNase), proteins were digested with Proteinase K at 50°C for overnight. DNA was then purified with phenol‐chloroform and resolved by agarose gel electrophoresis. Images were obtained and analyzed using an Agilent 2100 bioanalyzer.

### 2.8 Southern Blotting assay

A piece of agarose gel containing MNase digested DNA from both control and VP9‐transfected cells was placed in 0.25 N HCl solution for 7 minutes for depurination, following by denaturation in 4 N NaOH solution for 30 minutes. The DNA was then neutralized in a buffer containing 0.5 mol/L Tris, 1.5 mol/L NaCl, and 1 mmol/L EDTA and transferred onto a nylon membrane by incubating in 20X SSC buffer (3 mol/L NaCl, 300 mmol/L sodium citrate, pH 7.0). To immobilize the DNA, the membrane was baked at 80°C for 4 hours. Pre‐hybridization was performed at 42°C for 6 hours in a pre‐warmed buffer containing 50% formamide (Invitrogen) and 100 μg/mL of sheared salmon sperm DNA. The probes targeting centromere boundary (probe 1) and pericentromeres (satellite 2, probe 2) were designed by referring to an early study.^52^ They were synthesized with a Digoxigenin (DIG) tag by GeneDesign, Inc Japan. After overnight hybridization at 52°C for the centromere boundary probe and 49°C for the pericentromere probe, highly stringent washes were carried out (0.5x SSC buffer, 0.1% SDS at 65°C, two washes, each for 15 minutes). Southern blots were visualized using an Anti‐DIG fluorescent detection kit (Roche). The line scan images were quantitatively analyzed by the ImageJ software. The sequences of two probes are given below:

- Probe 1: Centromere boundary^52^
- TATTTTGACCTCTTTGAGGCCTTCGTTGGAAATGGGATTTCTTCA;
- Probe 2: Satellite 2 (NCBI Assembly DB AC253579)
- AGGAGTCATCATCTAATGGAATTGCATGGAATCATCATAAAATGGAATCG

### 2.9 Microarray

Total RNA was extracted from the control and VP9‐transfected HeLa cells at 24 hpt and labeled with a low Input Amp Labeling Kit, One‐Color (Agilent p/n 5190‐2305) following manufacturer's instructions (One‐color Microarray‐based Gene Expression, analysis low Input Quick Amp Labeling, version 6.5). Briefly, 100 ng of total RNA was converted into double‐stranded cDNA by priming with an oligo‐dT primer containing the recognition site for T7 RNA polymerase. 600 ng of labeled cDNA was hybridized onto Agilent SurePrint G3 Human GE 8 X 60K Microarray slide. The raw signal data were extracted from the TIFF image with Agilent Feature Extraction Software (V10.7.1.1). The robust multiarray analysis (RMA) value for each gene was obtained in triplicate, and their *P* values were computed by student T‐test. Genes with *P* value < 0.05 were considered to be significantly altered, and a cutoff of 1.2 fold was used to identify differentially expressing genes. DAVID bioinformatics resources^53^ were used for gene ontology (GO) analysis.

## 3 RESULTS

### 3.1 VP9 is enriched in the nucleus during the early stage of its expression in HeLa cells

**Figure 1:**
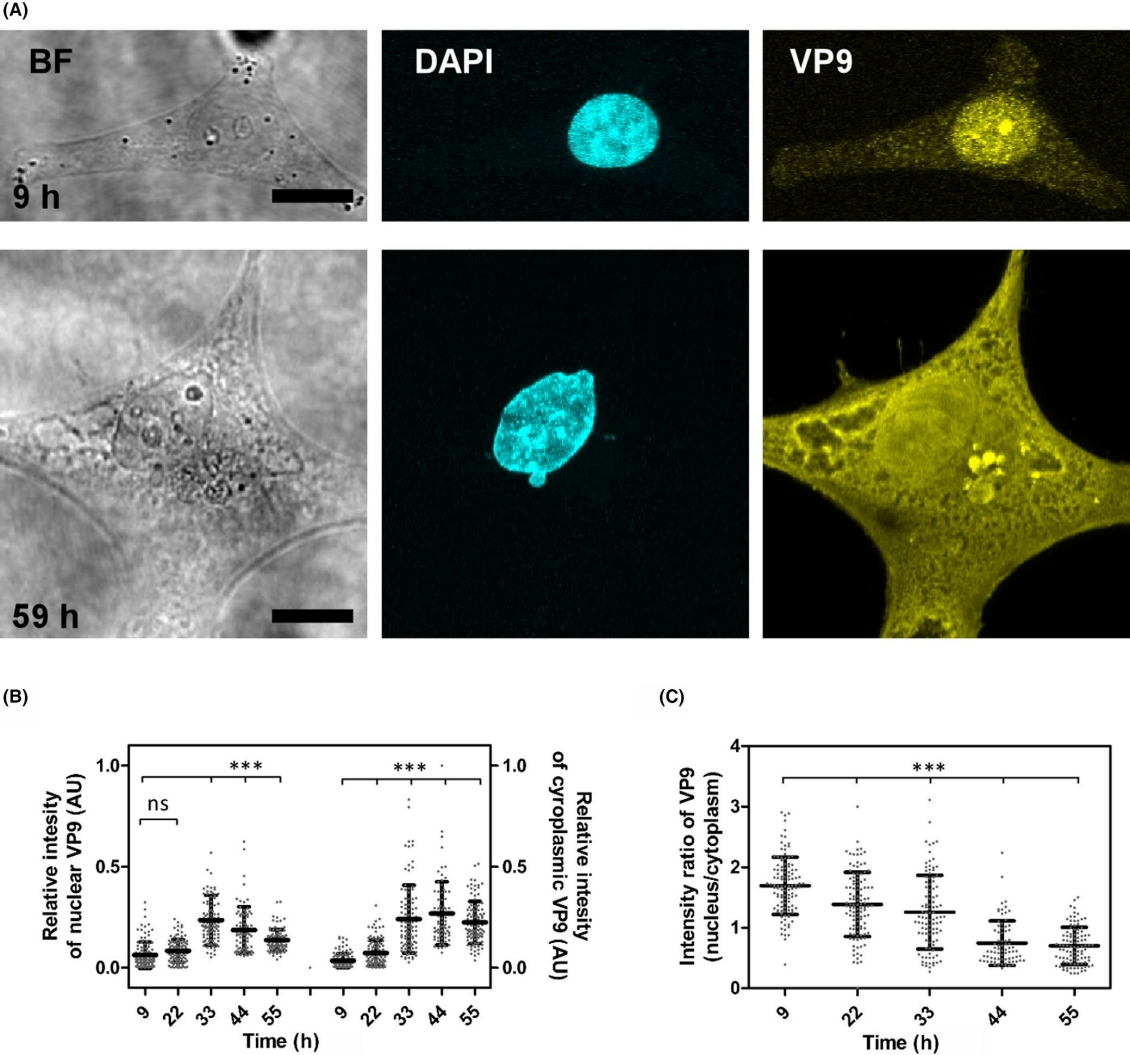
Localization of expressed VP9 in HeLa cells. A, Representative bright field and confocal images of VP9 distribution at 9 and 59 h post‐transfection. VP9 immunostaining was visualized with yellow fluorescence, and the DAPI counterstain was visualized in cyan. Scale bar = 15 μm; B, The relative intensity of VP9 in the nucleus and cytoplasm over time was quantified with MATLAB software (mean ± SD of five independent experiments, number of cells examined is between 76 and 114). C, The ratio of VP9 intensity in the nucleus vs the cytoplasm over time shows the majority of VP9 was translocated into the nucleus in the early phase after expression. Significant difference is determined by One‐way ANOVA, ***P < .001; ns, not significant; VP9, viral protein 9

HeLa cells were transfected with pXJ40‐VP9 plasmids. VP9 expression and distribution in the cell were examined at 9, 22, 33, 44, and 55 hours post‐transfection (hpt) by immunostaining with a validated mouse monoclonal antibody.^9^ Two representative cells at the early (9 hpt) and the late stage (55 hpt) are shown in Figure 1A, and quantitative analysis of VP9 expression at all the five time points are presented in Figure 1B,C. VP9 was expressed at a higher intensity in the nucleus compared to the cytoplasm at the three early time points, but this decreased over time (Figure 1C). In the early stage (9 hpt), although overall VP9 abundance was low in both nucleus and cytoplasm, approximately 87% (99 out of 114 examined cells) of cells exhibited relatively enriched VP9 in the nucleus. In the middle stage (between 22‐33 hpt), VP9 abundance was increased in the both nucleus and cytoplasm, and remained relatively high in the nucleus. After 33 hpt, VP9 expression appears to have reached a plateau and starts to decline. The intensity ratio of VP9 between the nucleus and the cytoplasm also decreases, with slightly more VP9 present in the cytoplasm. These results indicate that the majority of expressed VP9 was transported into the nucleus in the early phase of expression and remained highly abundant until 33 hpt. A previous study revealed that VP9 (named ICP11) was detected in the nucleus and the cytoplasm of hemocytes from WSSV‐infected shrimp,^12^ which suggests that the expressed recombinant VP9 has a similar cellular distribution in HeLa cells as it does in WSSV‐infected shrimp cells. Therefore, we proceeded to explore VP9 function in transfected HeLa cells.

### 3.2 VP9 impedes the interaction between DNA and histones in vitro

The crystal structure of VP9 has shown that it can act as a DNA mimic.^13^ Since VP9 localizes to the nucleus in transfected HeLa cells and WSSV‐infected shrimp cells, we performed a preliminary in vitro single molecule manipulation study via transverse magnetic tweezers to examine whether VP9 impedes histones binding to DNA.

A pilot experiment was first carried out to investigate whether VP9 directly interacts with DNA by testing the extension (elasticity and stiffness) of DNA. Naked dsDNA was incubated with four different concentrations of VP9 (0, 0.01, 0.1, and 1 mg/mL) and tested under four different forces separately (0.17, 0.46, 1.19, and 3.21 pN) (Figure S1A). The extension of DNA did not change significantly under all tested conditions over a period of time (0‐600 seconds) (Figure S1B), which suggests that there was negligible direct interaction between VP9 and DNA.

The histone chaperon protein NAP1 has been previously demonstrated to facilitate the formation of nucleosomes.^54^ NAP1 was used as a positive control to ensure that experimental conditions were well suited for nucleosome formation. Upon incubation of DNA with NAP1, stepwise folding of dsDNA was observed under a constant force of 6 pN (Figure S2A) and application of 15 pN pulling force caused stepwise unfolding (Figure S2B). Detailed investigation of the stepwise unfolding data revealed that the DNA unfolded with a 50 nm step size (Figure S2C), which corresponds to a DNA length of approximately 150 bp wrapping around one histone octamer.^35^

Under the same experimental setup, we investigated the effects of different concentrations of VP9 on histones binding to DNA. In a control experiment, histone octamers (100 nmol/L) alone were incubated with dsDNA. This caused a dramatic decrease of the dsDNA extension by 4 μm under the force of 6 pN, but there was no stepwise folding under this constant force. When the force was decreased gradually to 0.19 pN, the dsDNA extension also gradually decreased to approximately 1 μm (Figure S3A). However, when the force was reversed back from 0.19 to 6 pN, the extension of the dsDNA remained about 1 μm and was unable to be stretched (Figure S3A, red arrow). This suggests that dsDNA was strongly associated with histones and condensed. As expected, the typical stepwise folding and unfolding of nucleosomes, corresponding to nucleosome assembly and disassembly, respectively, was not observed in the absence of histone chaperone NAP1.

However, when a solution of pre‐mixed VP9 (10 µmol/L) and histone octamers (100 nmol/L) was incubated with dsDNA, no dramatic shortening of dsDNA extension was observed. When the force was reduced to 0.19 pN, the DNA extension was reduced to 10.5 μm (Figure S3B). The dsDNA could be stretched slightly to a distance of up to 6 μm when the force was reversed back to 6 pN (Figure S3B, red arrow), suggesting that VP9 diminished the binding capacity of histones on the DNA tether. When the VP9 concentration was increased to 100 μmol/L, a stronger interference in the interaction of dsDNA‐histones was observed as demonstrated by the reduction of DNA extension to 10 μm at 0.19 pN (Figure S3C), and an increase of the extension to 11 μmol/L upon reversal to a 6 pN force (Figure S3C, red arrow). These experimental data indicate that VP9 weakened the interaction between dsDNA and histones, and DNA compaction by histones.

Bovine serum albumin is a negatively charged protein under neutral pH with an isoelectric point (IP) of 4.7, similar to the VP9 IP of 4.2. When pre‐mixed BSA (100 μmol/L) and histone octamers (100 nmol/L) was added to dsDNA, it caused a sudden reduction in dsDNA extension to 2 μm at 6 pN, and this extension was largely reduced when the force decreased from 6 to 0.88 pN, but the extension did not decrease further with the further lowering of the force to 0.19 pN (Figure S3D). In addition, no stretching of the dsDNA was observed under the 6 pN pulling force (Figure S3D, red arrow). This result indicates that VP9 specifically impedes the interaction(s) of dsDNA and histones.

### 3.3 Histone H3 displays a higher mobility in the VP9‐transfected cells

Although VP9 has been proposed to disrupt nucleosome assembly,^13^ there is no direct evidence to support this hypothesis. It was also reported that when the histone H3 proteins were pre‐incubated with dsDNA, VP9 was unable to displace dsDNA from the histone‐dsDNA complex.^13^ This suggests that VP9 might interfere with de novo nucleosome formation, but might not exert a strong enough effect to disrupt existing nucleosomes, the primary chromatin structure. Additionally, our single‐molecule manipulation experiments also indicate that VP9 causes a moderate weakening of histone‐DNA interactions. On the basis of these initial findings, we examined how VP9 might affect the interplay between histones and DNA within a living cell by FRAP assay.

**Figure 2:**
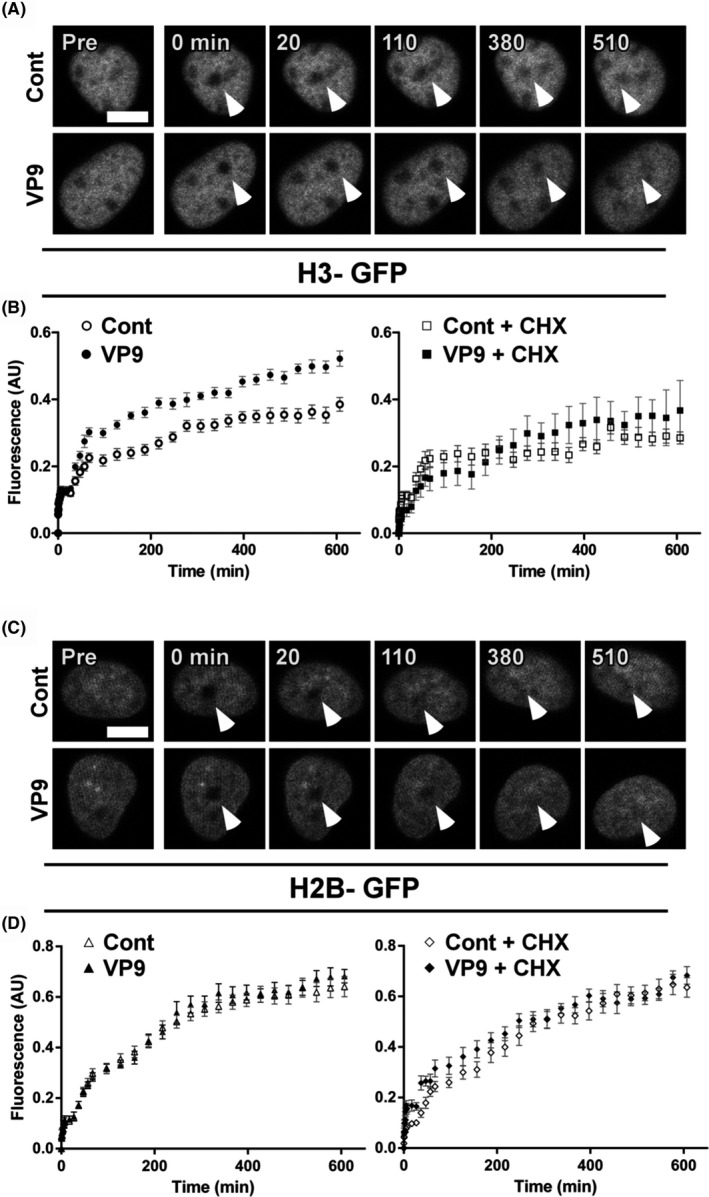
FRAP analysis of histone kinetics in VP9‐transfected HeLa cells. A, Time‐lapse imaging of control‐ and VP9‐transfected H3‐GFP stable cell line, arrows indicate the bleached area. Scale bar = 10 μm; B, FRAP curves of H3‐GFP are shown as mean ± SE (standard error), four independent experiments, n = 10‐32 cells; Left: H3‐GFP cells after 24 h post‐transfection (hpt) with plasmid pXJ40‐VP9 before bleaching; Right: H3‐GFP cells after 24 hpt with plasmid pXJ40‐VP9 with addition of cycloheximide (CHX) one hour before bleaching. C, Time‐lapse imaging of control‐ and VP9‐transfected H2B‐GFP stable cell line after bleaching, arrows indicate the bleached area, Scale Bar = 10 μm; (D) FRAP curves of H2B‐GFP are shown as mean ± SE, four independent experiments, n = 10‐19 cells; Left: H2B‐GFP cells after 24 hpt with plasmid pXJ40‐VP9 before bleaching; Right: H2B‐GFP cells after 24 hpt with plasmid pXJ40‐VP9 with addition of CHX one hour before bleaching. FRAP, fluoresecence recovery after photobleaching; VP9, viral protein 9

HeLa cells were co‐transfected with pXJ40‐VP9 (non‐fluorescent) and pXJ40‐mCherry plasmids. The expression of mCherry was used to optimize transfection conditions and to select transfected cells for experiments (Figure S4). The recovery of bleached spots (Figure 2A,C, arrow heads) was recorded for a period of approximately 558 minutes (Figure 2B,D). The rapid fraction of subpopulation of histones recovered within one minute. The very slow fraction refers to the histones that recovered too slowly to be monitored within 558 minutes, and the slow fraction refers to the histones recovered between the rapid and very slow fractions.^42^

In the VP9‐transfected H3‐GFP cells, the recoveries were fast at the beginning and gradually gained until 558 minutes, achieving approximately 50% of pre‐bleached intensity. In contrast, control‐transfected H3‐GFP cells demonstrated slow recoveries and reached a plateau after 400 minutes, only achieving approximately 37% of pre‐bleached intensity (Figure 2B, Left column). The rapid fraction was similar in the both control‐ and VP9‐transfected cells. However, the slow fraction of H3 in VP9‐transfected cells was significantly larger than in control‐transfected cells (38% vs 27%), and the very slow fraction in VP9‐transfected cells was less than in control‐transfected cells (52% vs 62%) (Table 1). These data indicate that H3 displayed a higher mobility in VP9‐transfected cells compared to that in the control. The increased proportion of the slow fraction of H3 in VP9‐transfected cells likely came from the very slow fraction, suggesting that a proportion of this fraction, which is probably associated with heterochromatin,^42^ became more mobile upon VP9 expression.

**Table 1:** Information of different kinetic populations of histone‐GFP revealed by FRAP

	Histone	Rapid		Slow		Very slow	
		Half‐life (min)	Fraction (%) (±SE)	Half‐life (min)	Fraction (%) (±SE)	Half‐life (min)	Fraction (%)
Control cells	H3GFP	0.4	11.0 ± 0.5	142.7	27.0 ± 0.9	>510	62.0
VP9‐transfected cells	H3GFP	0.4	10.0 ± 0.5	103.7	38.0 ± 0.7	>510	52.0
Control cells	H3GFP+CHX	0.3	7.0 ± 0.9	39.9	19.4 ± 1	>510	73.6
VP9‐transfected cells	H3GFP+CHX	3.4	7.5 ± 2.7	191.1	32.0 ± 4	>510	60.5
Control cells	H2BGFP	0.4	7.0 ± 0.9	125.8	58.7 ± 1.4	>510	34.3
VP9‐transfected cells	H2BGFP	0.4	8.0 ± 0.9	150.5	63.3 ± 1.7	>510	28.7
Control cells	H2BGFP+CHX	0.5	6.5 ± 1	195.1	65.5 ± 3	>510	28.0
VP9‐transfected cells	H2BGFP+CHX	0.6	14.0 ± 1	166.5	54.3 ± 2.2	>510	31.7

Abbreviation: CHX, cycloheximide; FRAP, fluoresecence recovery after photobleaching; VP9, viral protein 9.

In order to exclude the possibility that the increased mobile fraction of H3 resulted from an increase in H3 synthesis upon VP9 expression, another set of FRAP experiments was performed using cycloheximide (CHX) to inhibit protein synthesis.^55^ Inhibition of protein synthesis slowed down H3‐GFP recoveries within both control and VP9‐transfected groups, but the mobility of H3‐GFP as well as mobile fractions remained higher in VP9‐transfected cells than that of the control (Figure 2B, right column, Table 1). Taken together, these FRAP experiments indicate that VP9 expression alters H3 dynamics. VP9 may impede H3‐DNA interaction and thus loosen up chromatin compaction. In agreement with a previous report that VP9 has relatively mild interaction with H2B,^13^ VP9‐expression seemingly did not induce obvious changes in H2B mobility (Figure 2D, Table 1).

### 3.4 The compaction level of higher‐order chromatin is reduced in VP9‐transfected cells

**Figure 3:**
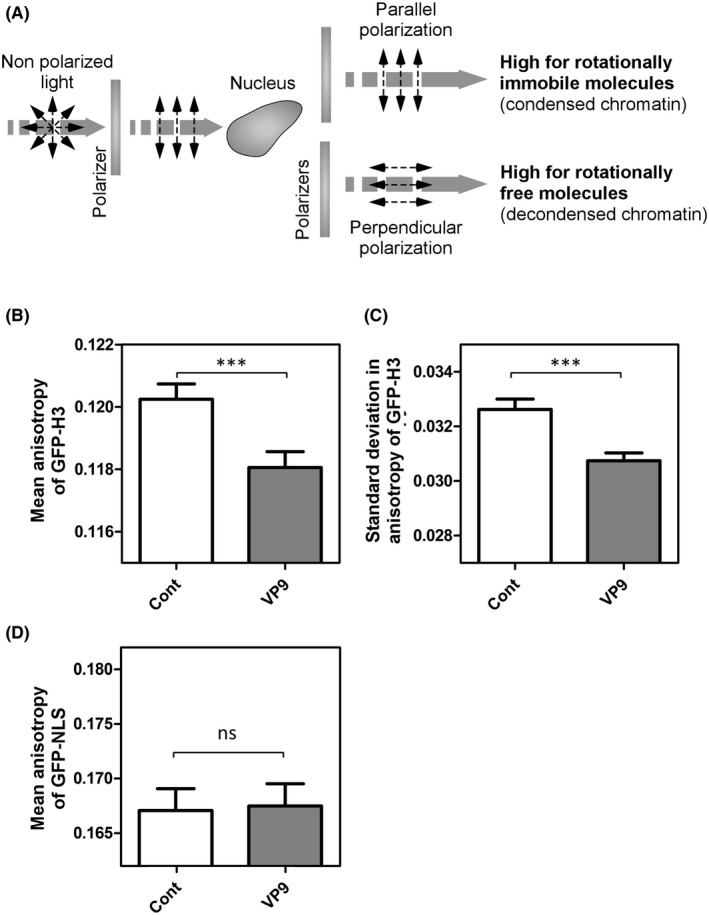
Fluorescent anisotropy of nuclei of control‐ and VP9‐transfected H3‐GFP stable cells at 24 h post‐transfection. A, Schematic illustration of fluorescence polarization in anisotropy. When linearly polarized light is incident on a fluorescent sample, the depolarization of emission is proportional to the rotational mobility of the fluorophores; B, Bar graph of mean anisotropy (*r* ± SEM, n = 50 individual cells from 1 replicate); C, Bar graph of the standard deviation (sd(*r*) ± SEM, n = 50) of anisotropy. An overall reduction of the mean anisotropy indicates global decondensation of the chromatin; D, Bar graph of mean anisotropy of GFP‐NLS (*r* ± SD, n = 10 individual cells from 1 replicate). ***P < .05; ns, not significant; VP9, viral protein 9

Our FRAP data demonstrated that VP9 increases histone mobility and possibly loosens up chromatin structure. Given that cell death also leads the changes in chromatin architecture, we performed the fluorescence‐activated cell sorting (FACS) assay after dual staining with annexin V and DAPI. As a result, we found VP9 expression did not give rise to significant apoptotic and necrotic cell death (Figure S5). Therefore, we further examined whether VP9 could bring about changes in chromatin compaction via two experiments: FAI and salt fractionation. Since histone core proteins are key components of nucleosomes, which subsequently assemble as chromatin, histone mobility is an indicator of chromatin compaction.^56^ Using FAI, we measured the value of anisotropy (r) of GFP tagged H3 histones. Low anisotropy values represent high rotational mobility of H3, indicating high fluidity (suggesting low compaction) of chromatin^45^, ^46^ (Figure 3A).

The anisotropy images for representative control and VP9‐transfected cells were plotted with color‐coding maps to differentiate various levels of fluorophore rotational mobility and therefore chromatin fluidity (Figure S6). Highly fluid regions (lower anisotropy) are shown in blue while highly immobile regions are shown in dark red (higher anisotropy). In order to compare the average chromatin compaction and heterogeneity of compaction within the entire nucleus, we quantified mean (*r*) and standard deviation (sd(*r*)) of H3‐GFP anisotropy in control‐ and VP9‐transfected stable H3‐GFP cells (n = 50) (Figure 3B,C). Both the mean (*r*) and sd(*r*) were significantly lower in VP9‐transfected cells than in control cells (*P* < .05), denoting a high rotational mobility (low anisotropy) of H3‐GFP and an overall low level of chromatin compaction (high fluidity) in VP9‐transfected cells. This is consistent with the results of our FRAP experiments, where VP9 expression led to the increase in the slow mobile fraction of H3. In contrast, anisotropy of a nuclear‐localized recombinant protein GFP‐NLS, which does not interact with DNA, was not affected by VP9 expression (Figure 3D), proving the specificity of VP9 on H3 mobility.

Changes in the core histone mobility may partially or temporarily interrupt the geometric and mechanical properties of nucleosomes, which could exert an influence on chromatin solubility.^57^ Therefore, we carried out salt‐fractionation experiments according to Henikoff's method^43^ to investigate whether the core histones become loose and could be easily solubilized.

**Figure 4:**
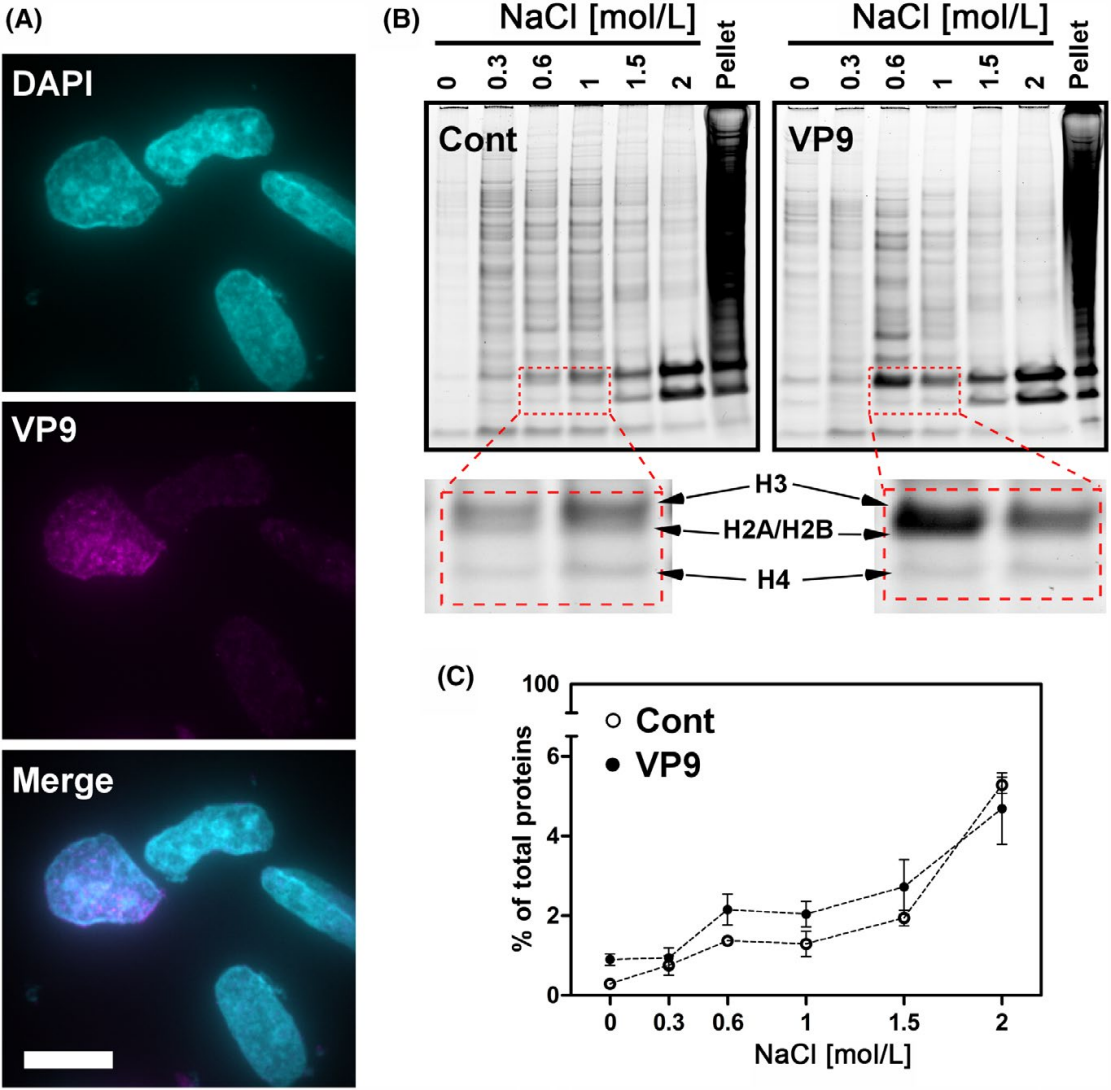
Successive salt fractionation of histone proteins at 24 h post‐transfection. A, Representative image of isolated nuclei from VP9‐transfected cells; VP9 (magenta), DNA (cyan), Scale bar = 15 μm; B, SDS‐polyacrylamide gel analysis (SDS‐PAGE) of histone proteins from control or VP9 expressing cells following successive salt extraction steps; The bottom inserts show the relative amount of histones solubilized at 0.6 and 1 mol/L salt concentrations is significantly higher than the amount solubilized at 0.3 mol/L in VP9‐transfected cells, whereas this increase does not occur in control‐transfected cells; C, Quantitative analysis of the proportion of soluble histone fractions including H3, H2A, H2B, and H4, in total nuclear proteins (mean ± SE from three independent experiments), showing the high amount of histones eluted from VP‐transfected cells at 0.6, 1, and 1.5 mol/L salt concentrations. VP9, viral protein 9

The presence of VP9 was confirmed in the isolated nuclei by immunofluorescent staining (Figure 4A) although VP9 was largely lost during the isolation of nuclei due to its small size and permeabilization treatment with Igepal CA‐630. Previous studies reported that ~90% of histones were solubilized in high‐salt concentrations (0.6 mol/L up to 2 mol/L NaCl).^42^, ^43^ These are classified as the higher‐salt soluble fractions, representing oligonucleosomes that are attached to large protein complexes to form bulk chromatins, whereas the low‐salt soluble histone fractions (below 0.25 mol/L) are mostly constituted of short nucleosomal strings at a mononucleosome level, representing a small proportion of total chromatin.^58^, ^59^ In the non‐successive extraction, the relative amount of soluble histone fractions in VP9‐transfected cells at the low‐salt concentrations (0 and 0.3 mol/L) did not change much compared with the control‐transfected group, whereas the histone fraction at the 0.6 mol/L salt concentration increased more than 4‐fold (~60%) in VP9‐expressing cells (Figure S7). Increasing salt concentration further to 1, 1.5, and 2 mol/L did not lead to an increase in eluted histones. In contrast, a clear increase in solubilized histones was observed when the salt concentrations were increased from 1 to 2 mol/L in control‐transfected cells. Taken together, the results suggest that VP9 alters bulk chromatin structure, and these changes are likely to be in the higher order heterochromatin.

The successive fractionation stepwise elutes the subpopulations of histones that are associated with different levels of chromatin compaction.^60^ A similar trend was observed as in the non‐successive salt fractionation experiment, with no obvious increase of the histone fractions solubilized at the low‐salt concentrations (0 and 0.3 mol/L), which might indicate that VP9 transfection did not result in obvious alteration of “active chromatin.” In contrast, a prominent subpopulation of histones were solubilized at the 0.6 mol/L salt concentration (Figure 4B, dotted red square) and the relative amounts of soluble histones in extracts taken at 1 and 1.5 mol/L NaCl were also higher in the VP9‐transfected group than in the control group (Figure 4C). This suggests a less condensed bulk chromatin structure following VP9 transfection and 24 hours of expression.

### 3.5 VP9 expression increases the accessibility of heterochromatin by MNase

**Figure 5:**
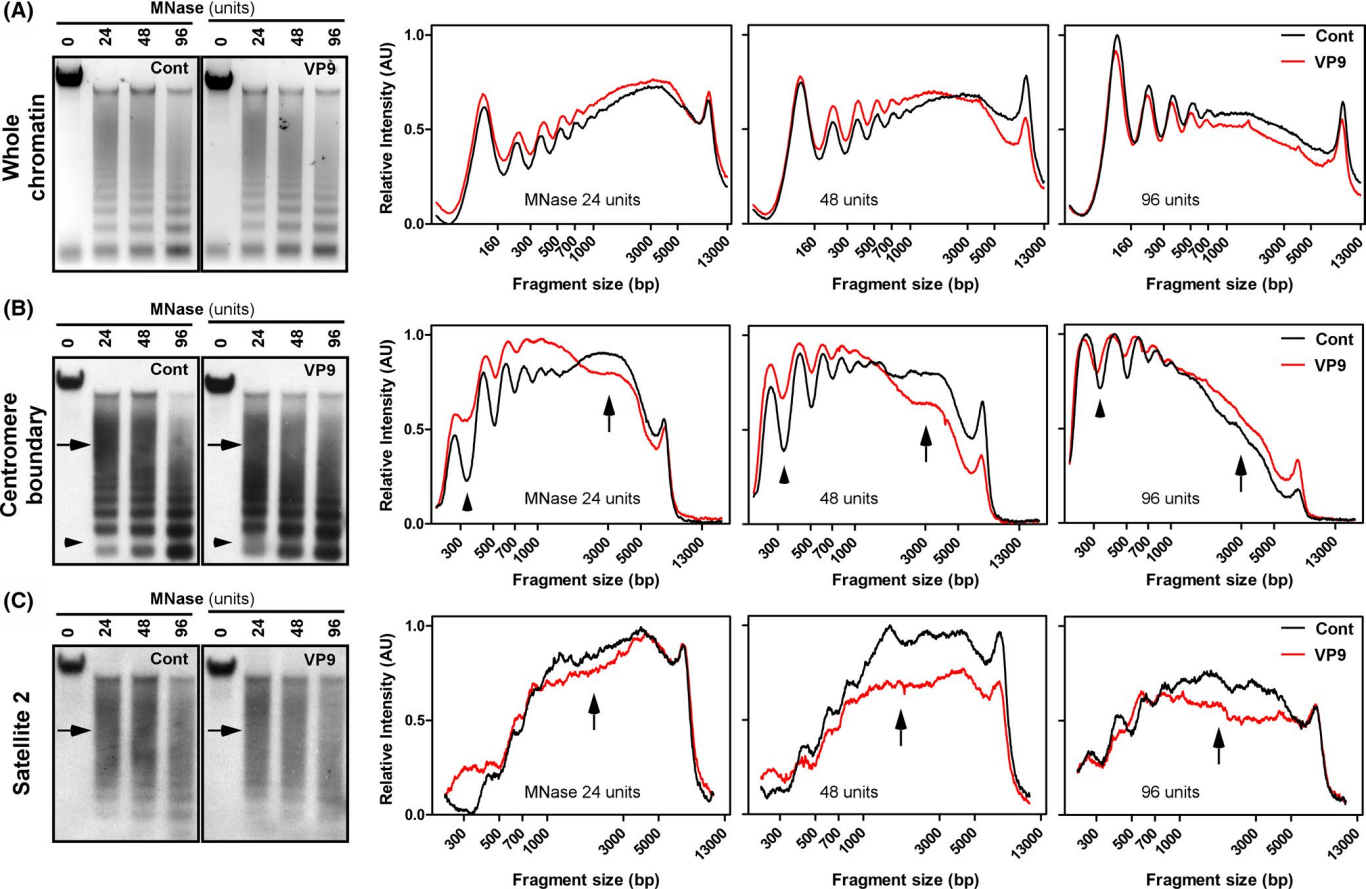
VP9 increases the MNase accessibility to juxtacentromeric chromatins at 24 h post‐transfection. A, Control and VP9‐transfected cells were pre‐permeabilized and treated with different concentrations of MNase. 5 μg purified DNA from each treatment was separated on one agarose gel, stained with Sybr safe (left) and analyzed by Image J (right). There was no obvious difference in the DNA profile following MNase digestion in control or VP9 cells; B, Separated DNA on the agarose gel was blotted onto a nylon membrane and hybridized with a probe targeting the centromere boundary. MNase treatment produced DNA ladders with higher densities (arrowheads on electropherogram map) and retained less incomplete digestion (arrows) in VP9‐transfected cells compared to control cells. C, A parallel experiment was carried out with the satellite 2 probe, which shows much less incomplete digestion of pericentromeres (arrows on the eletropherograms) in VP9‐transfected cells compared with its control. This experiment suggests the juxtacentrimeric region (comprising the pericentromere and centromere boundary) is altered and becomes more accessible to MNase upon VP9 expression. VP9, viral protein 9

The results of our salt fractionation experiments hint that VP9 expression alters higher order chromatin structure (correlated with bulk chromatins), but may not cause dramatic unfolding of chromatin fibers to the mononucleosome level, because the fraction of histones at low salt concentrations did not change significantly. In order to determine the level of chromatin unfolding, we performed MNase digestion assays.^61^ Following gel electrophoresis, quantitative analysis revealed that the overall DNA profiles of VP9‐ and control‐transfected cells were similar in terms of intensity and the number of DNA bands (Figure 5A). The DNA ladder further indicated that nucleosomes were maintained in VP9‐transfected cells. These data support the hypothesis that VP9‐transfection does not unwind chromatin to the mononucleosome level.

Since the overall DNA profiles after MNase treatment did not show a discrete change, we designed specific probes associated with heterochromatin regions to investigate whether there are distinct regional changes by southern blotting. A typical DNA ladder profile was observed following visualization with Probe 1, which targeted the centromere boundaries (Figure 5B). However, intensity scans showed that DNA ladders generated from VP9‐transfected cells were denser than the corresponding bands from control‐transfected cells after low (24 U) and moderate (48 U) MNase treatment (Figure 5B, arrow heads). More importantly, the level of incompletely digested DNA is reduced in VP9‐transfected cells compared to control‐transfected cells (Figure 5B, arrows). These results indicate that the highly condensed chromatin at the centromere boundaries became more accessible to MNase digestion following VP9 expression, suggesting that the highly compacted heterochromatin has become less condensed. In addition, the density of the smear between the ladders (Figure 5B, southern blot image) was higher following VP9 expression than in control cells, which may result from multiple cleavages of the linker regions between nucleosomes. This would indicate that the stability of linker histone association with histone core proteins was reduced and the centromere boundary regions might be loosened upon VP9 expression.

Probe 2 specifically binds to the pericentromeric heterochromatin.^62^ DNA ladders were not prominent in both VP9‐ and control‐transfected cells at all MNase concentrations, but the level of incompletely digested fractions were reduced in VP9‐transfected cells compared to control cells (Figure 5C, arrows), suggesting that pericentromeric regions also became more accessible to MNase digestion following VP9 expression. Taken together, our results suggest that VP9 expression alters juxtacentromeric heterochromatin structures around the centromere region.

**Figure 6:**
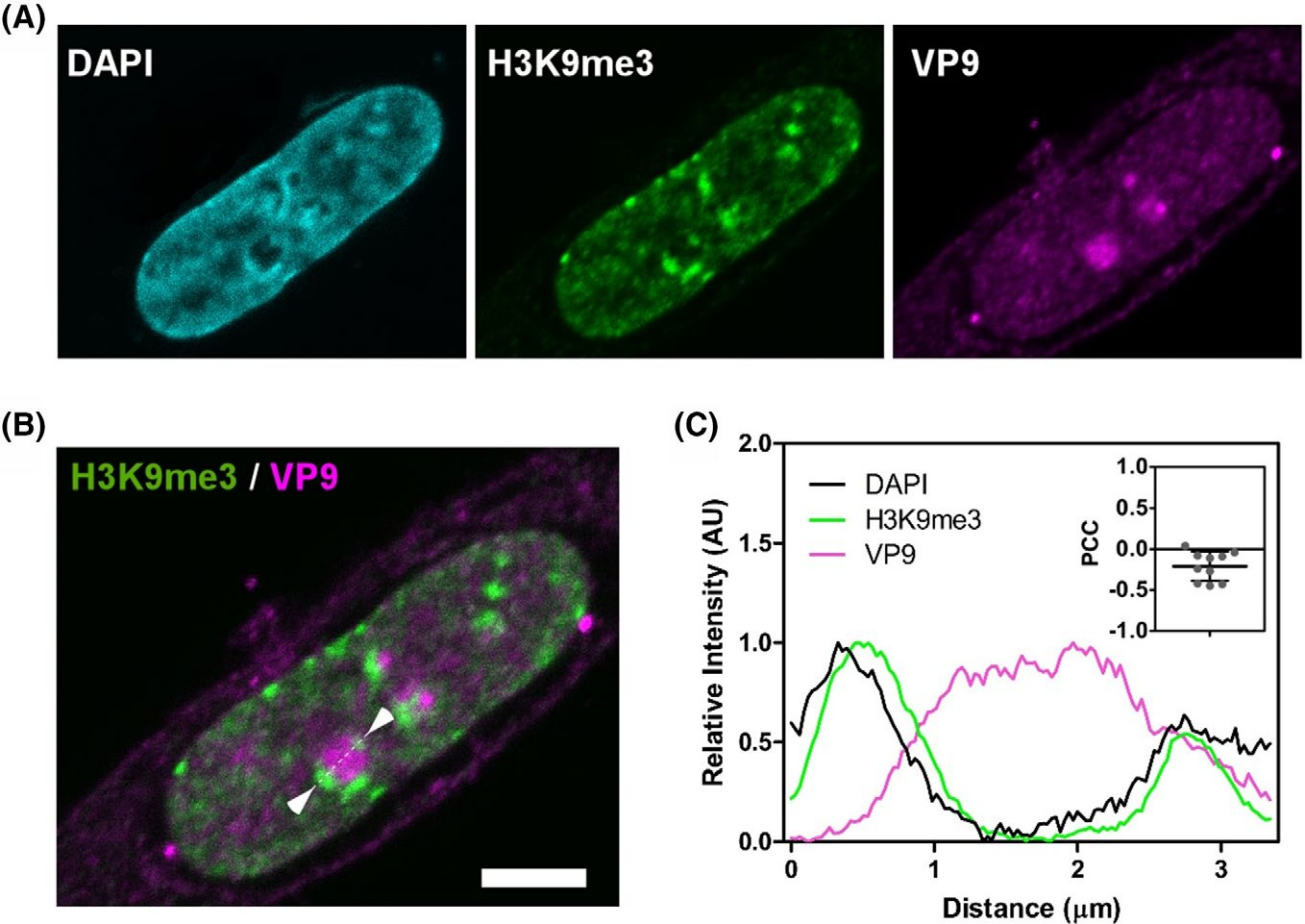
VP9 is closely associated with heterochromatin. A, A representative image of HeLa cells immunostained for H3K9me3 (green, a marker of heterochromatin) and VP9 (magenta), counterstained with DAPI (blue), scale bar = 5 μm; B, The merged image reveals that the histone modification marker H3K9me closely associates with VP9 granules, localizing at the concave capped regions at the edge of the VP9 granule (arrow heads). C, The density scanning profile across the VP9 granule (dotted line in B) confirms the spatial relationship between VP9 and heterochromatin. Pearson's correlation test provides a negative value, demonstrating a clear anti‐correlation. Therefore, where VP9 is present, heterochromatin is excluded, and vice versa (C inset), PPC, Pearson Correlation Coefficient; VP9, viral protein 9

We carried out confocal fluorescence microscopy to determine whether heterochromatin and VP9 were closely associated with each other. H3K9Me3 was used as the marker of heterochromatin^63^ (Figure 6A, green). At 24 hpt, VP9 was abundant in the nucleus, and appears to aggregate to form granules (Figure 6A, magenta). The merged image and density scanning demonstrated that highly‐concentrated VP9 was closely associated with H3K9Me3 (Figure 6B,C), which provides additional evidence that VP9 might be directly involved in the alteration of heterochromatin compaction.

### 3.6 VP9 expression alters cellular gene expression

Following our demonstration that VP9 is able to alter cellular chromatin structure, we carried out a preliminary investigation into whether this alteration causes changes in cellular gene expression. A microarray assay revealed more than 600 genes were differentially regulated upon VP9 expression (Table S1). These genes were sorted according to GO which revealed distinct gene clusters involved in transcription (23%), protein functions (13%), RNA processes (10%), apoptosis (6%), cell cycle (5%), and chromosome organization (3%) (Figure S8A). The remaining 40% of genes that did not fit into any of these categories were classified as having another function (Others, Figure S8A). With the caveat that HeLa cells are not a natural host for WSSV infection, we highlight expression changes for a few conserved pathways relating to DNA and RNA metabolism, apoptosis, nucleosome organization, and cell cycle regulation (Figure S8B), which may give hints for further exploration of VP9 function.

For example, markedly down‐regulated genes include transcription factor 2 (E2F), Bromodomain and WD repeat domain containing 1 (BRWD1), zinc finger 326 (ZNF326), and cyclin family members, which are involved in a variety of cellular processes including cell cycle progression and transcription regulation. The genes for kinesin family proteins, which are involved in chromosome positioning, centrosome separation, and establishment of a bipolar spindle during cell mitosis were also down‐regulated. On the other hand, genes involved in anti‐viral inflammation and pro‐apoptosis pathways were up‐regulated, such as MX dynamin‐like GTPase 1 (myxovirus resistance 1 [MX1]) and interferon‐induced protein 6 (IFI6).

## 4 DISCUSSION

Chromatin dynamics play important roles in both viral and host chromosome biology. As viruses are obligate parasites, they need to regulate host chromatin structure in both spatial and temporal dimensions in order to hijack the host machinery for self‐replication.^14^, ^15^, ^64^, ^65^ Although histone proteins are evolutionarily conserved, their spacing and epigenetic modification provide structural and signaling capacity to regulate chromatin and thus gene expression.^66^, ^67^ While viruses deploy various tactics to take over the host nucleus for replication and/or virion assembly, the highly organized cellular chromatin structure functions as a barrier,^68^ limiting the movement of viral machineries and their accessibility to the host genome. The nuclear viruses that can enter, egress, and persist in the nucleus must therefore contend with the forces that regulate host chromatin structure and drive chromatin rearrangement.

Although VP9 was identified as an abundant non‐structural viral protein and later revealed to be a DNA mimic,^13^ our single‐molecule manipulation experiment provides the first direct evidence that VP9 specifically impedes histones binding to dsDNA in vitro. In order to examine whether VP9 might affect chromatin structure inside a living cell, we first confirmed that VP9 was expressed and localized to the nucleus. FRAP experiments showed that VP9 expression increased the mobility of histone H3 in both the slow and very slow histone fractions, which may represent the histones associated with higher‐order structures of chromatin. FAI data further revealed that histones in VP9‐transfected cells display a high rotational mobility as well as heterogeneity in chromatin packaging. These results led us to investigate whether chromatin structure is altered due to the high mobility of H3 induced by VP9 expression. Interestingly, salt fractionation experiments revealed that significantly more histones were solubilized at a salt concentration of 0.6 mol/L in VP9‐transfected cells compared to control cells. Histones solubilized by high concentrations of salt (0.6 mol/L and above) are often associated with bulk chromatin (or oligonucleosomes), which supports the data from FRAP and FAI experiments. Furthermore, MNase treatment combined with southern blotting revealed that VP9 expression leads to more hydrolyzed heterochromatins at both centromere boundaries and the pericentromere, which are both regions of juxtacentromeric chromatin. Taken together, our results indicate that expression of VP9 in HeLa cells increases the mobility and solubility of histones, and alters the higher order chromatin structure. The results also demonstrate that VP9 is unlikely to unfold chromatin structure into single nucleosomes or disrupt basic nucleosome structures.

Currently only a few DNA mimic proteins have been identified.^69^ Although they have different functions, DNA mimics share a common mode of action through the control of DNA binding proteins involved in DNA repair, packaging, recombination, and expression. VP9 was identified as a DNA mimic through its crystal structure, which revealed two rows of negatively charged spots resembling the phosphate backbone of dsDNA. Our FRAP data revealed that VP9 expression increases the mobility of histone H3 significantly, but did not alter H2B mobility, demonstrating the specific interplay between VP9 and different histones. The mechanism of VP9‐histone interaction is unknown, but may result from spatial arrangement or epigenetic histone modifications.

Compared to hyperacetylated euchromatin regions, where the charges on the histone tails are neutralized at various levels, the charges on the individual lysine and arginine residues of methylated pericentromeric heterochromatin are not greatly affected.^70^ The different modifications between heterochromatin and euchromatin might provide a unique differentiator that favors interaction of VP9 with heterochromatin. Moreover, previous studies reported that proteins or peptides (eg, DELQPASITD) that induce chromatin decondensation usually contain a region of acidic‐hydrophobic amino acid residues, for example VP16 of HSV type 1 (aa 467‐479: ALDMADFEFEQMF), GAL4 (aa 861‐873: MDDVYNYLFDDED), p53 (aa 1‐73) and others.^71^ An intriguing alternate model suggests that the acidic‐hydrophobic activation motifs may act by interacting directly with histones, possibly distorting them structurally.^72^ The β1 (TFQTD), β2 (DFLLVG), and β6 strands (LEIQP) of VP9 also exhibit similar acidic‐hydrophobic properties, which could enable VP9 to induce chromatin decondensation. Alternatively, noticed that de novo interfering histone core proteins with DNA interactions may result in the reduction of the stability of linker histone protein association with histone core proteins. Cooperately, the spatial rearrangement of chromatin would tend to as the consequence of the deficiency in maintenance per se of nuclesomal integration.

In addition, the VP9 monomer structure contains a ferredoxin fold and NMR titration has shown that VP9 binds with both zinc and cadmium ions.^9^ Divalent ions have been shown to have various effects on nucleosome assembly and chromatin structure.^73^, ^74^ It would be interesting to investigate whether VP9 could alter chromatin structure via binding to divalent ions.^74^, ^75^, ^76^ The detailed mechanism of how VP9 alters higher order structure of chromatin needs to be further studied, and a key question that remains to be determined is whether VP9 functions as a monomer or polymer in WSSV‐infected cells.

Juxtacentromeric chromatin is largely transcriptionally inert, but plays critical roles in ensuring genomic stability and accurate chromosome segregation. It has been reported that disrupted juxtacentromeric chromatin leads to a failure in recruiting proteins required for mitotic progression (eg, Aurora B kinase), as well as a loss in retaining heterochromatin protein 1 (HP1), which lead to defects in chromosome segregation.^77^, ^78^ Our studies provide the first evidence that VP9 increases histone dynamics and alters the organization of juxtacentromeric chromatin. VP9‐triggered rearrangement of chromatin may hinder host cell division, less condensed chromatin structure may become vulnerable to be hijacked by the viral machinery while facilitating viral replication, which advances our fundamental understanding of WSSV replication strategy and pathogenesis.

## CONFLICT OF INTEREST

The authors declare that they have no conflicts of interest.

## AUTHOR CONTRIBUTIONS

X. T. designed and performed the majority of the experiments and drafted the manuscript. A. R. and O. H. T developed the analytical tools and guided the analysis. J.W and CLH guided this research and edited the manuscript.
