# Supplementary figures and images for: White spot syndrome viral protein VP9 alters the cellular higher‐order chromatin structure
Source: FASEB Bioadv. 2020 Mar 17;2(4):264–79. doi: 10.1096/fba.2019-00086 (PMC7133739; doi:10.1096/fba.2019-00086)

**A**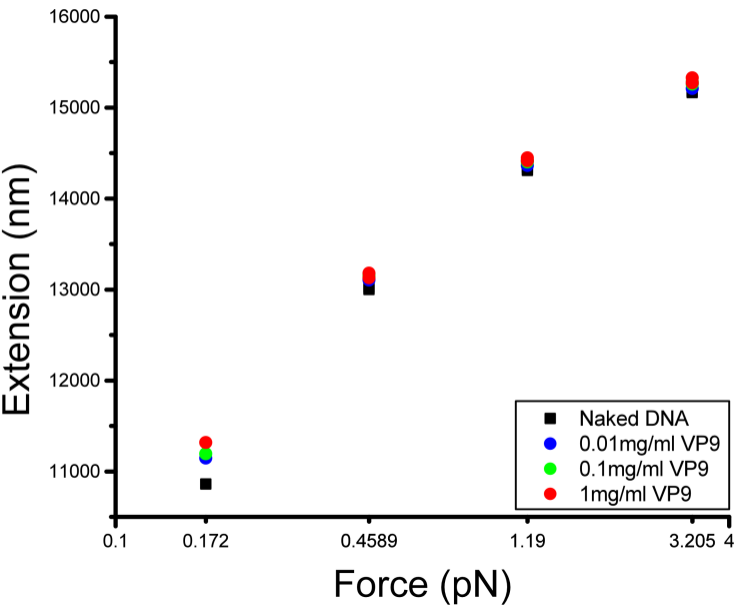**B**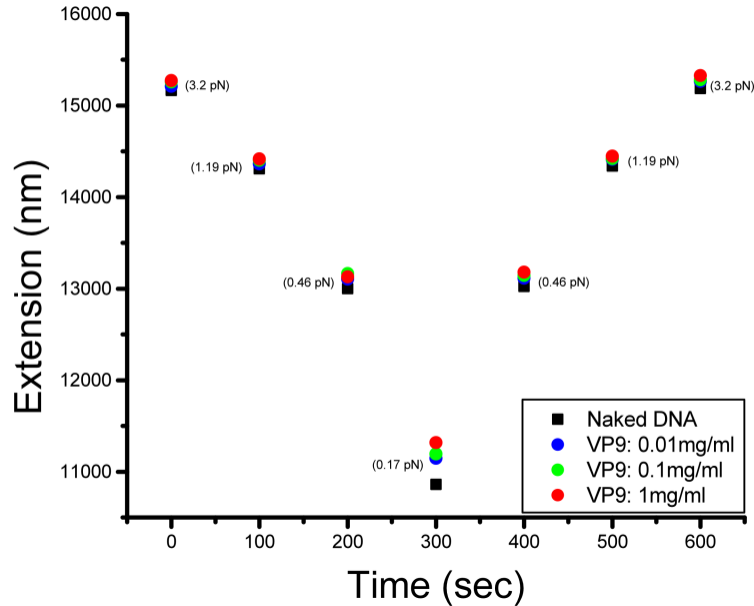

Supplement: Supplementary file 1 [file FBA2-2-264-s001.pdf]

**A**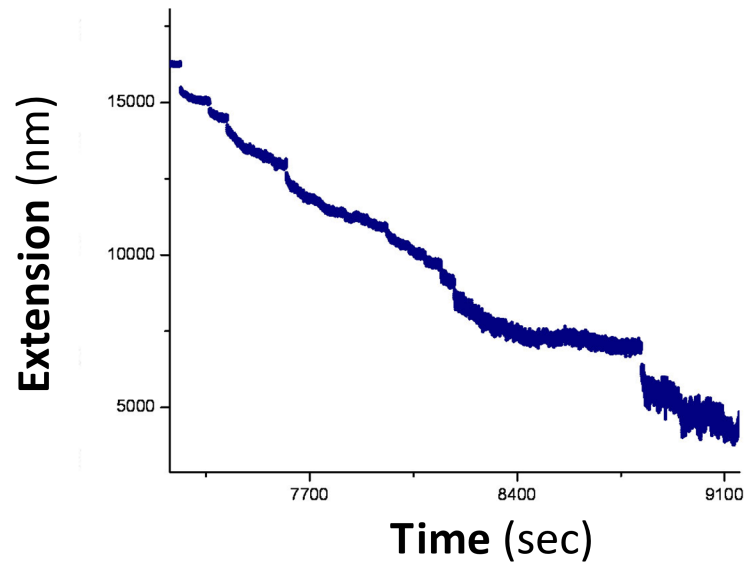**B**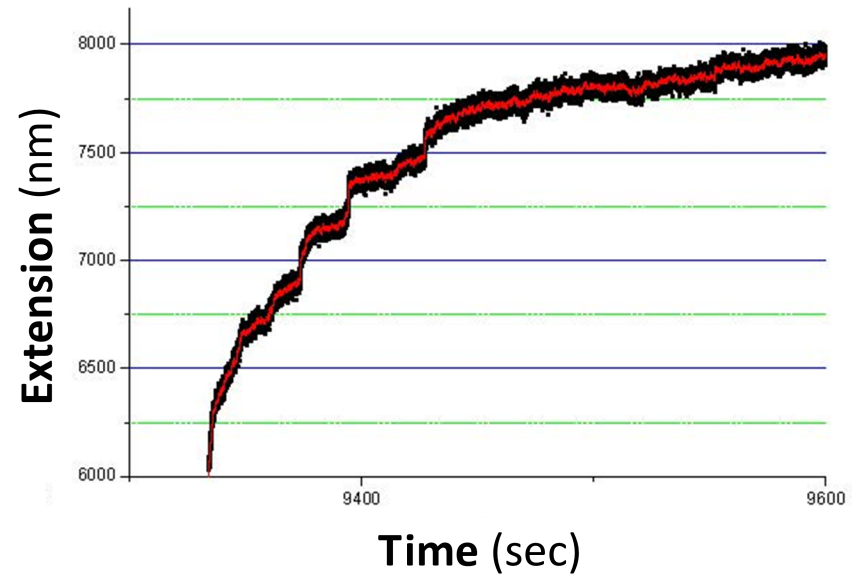**C**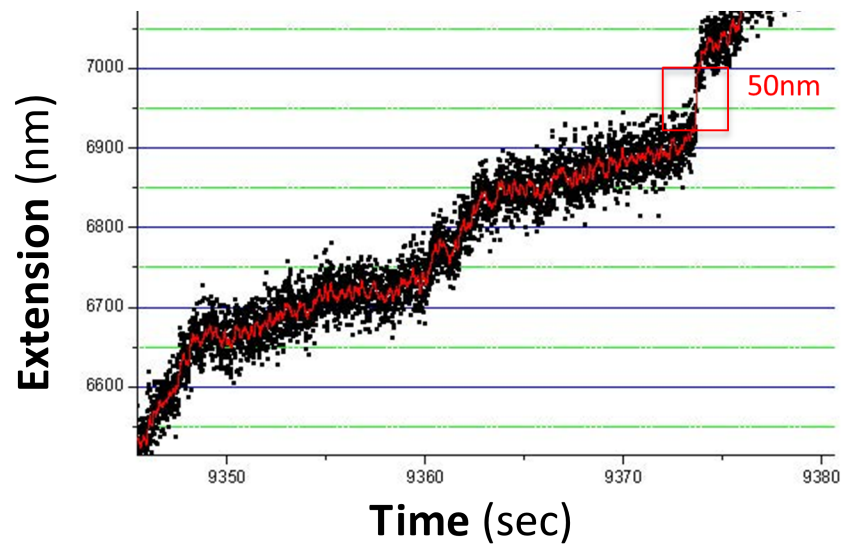

Supplement: Supplementary file 2 [file FBA2-2-264-s002.pdf]

**A**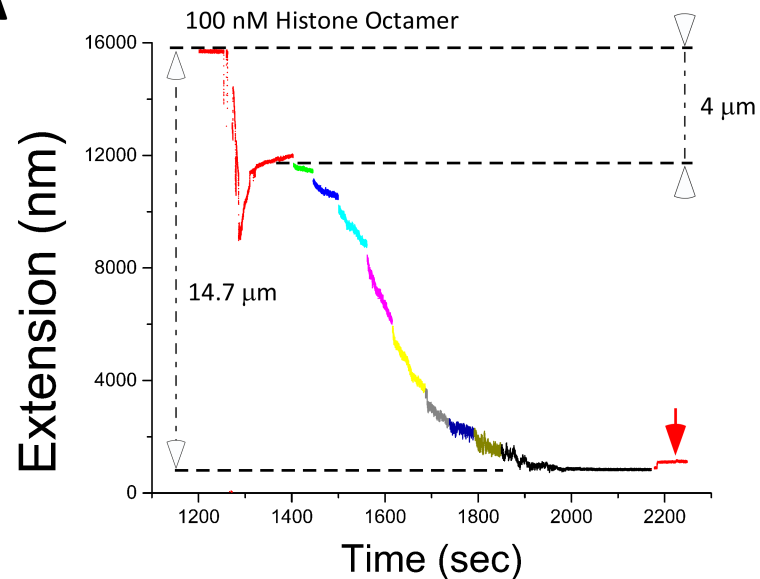**B**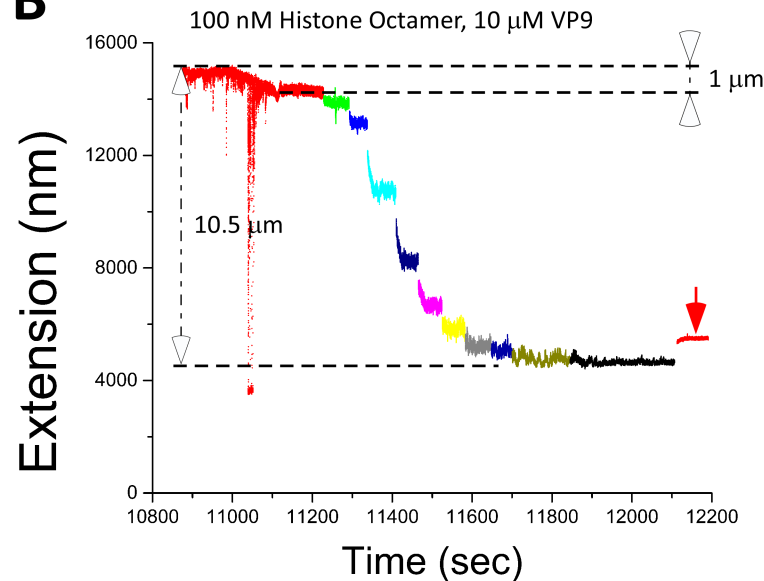**E**

|   |         |
|---|---------|
| — | 6.13 pN |
| — | 4.26 pN |
| — | 3.11 pN |
| — | 2.23 pN |
| — | 1.62 pN |
| — | 1.2 pN  |
| — | 0.88 pN |
| — | 0.66 pN |
| — | 0.41 pN |
| — | 0.29 pN |
| — | 0.22 pN |
| — | 0.19 pN |

**C**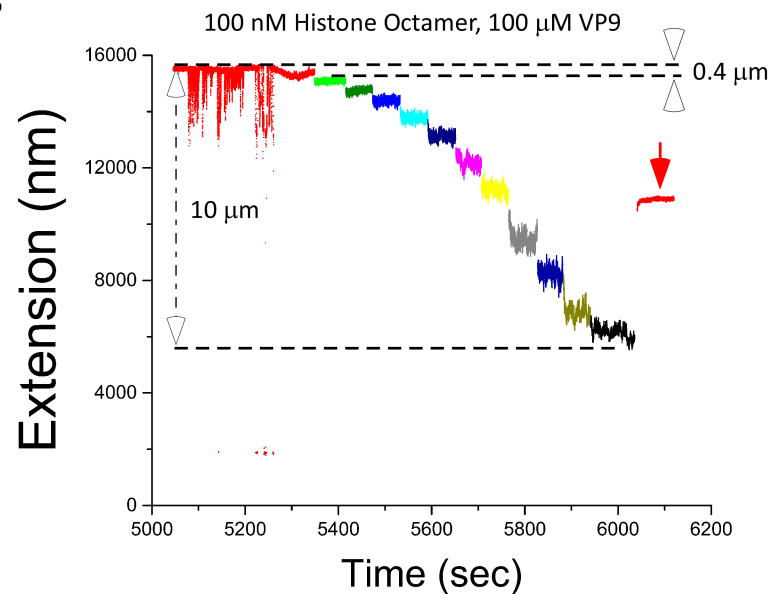**D**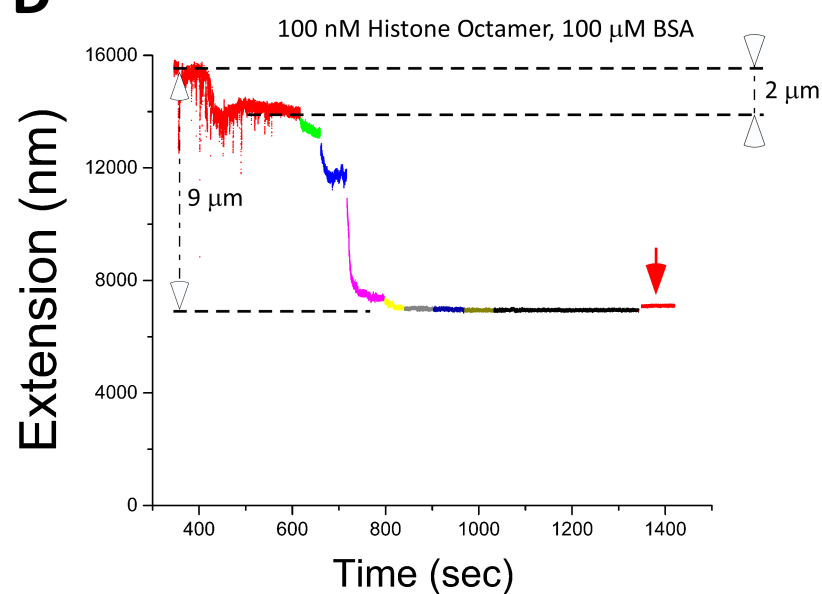

Supplement: Supplementary file 3 [file FBA2-2-264-s003.pdf]

Region 1

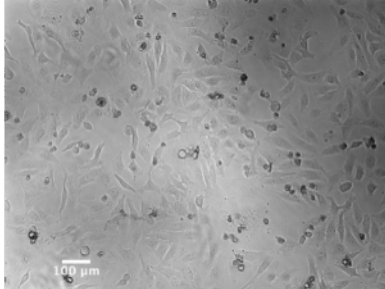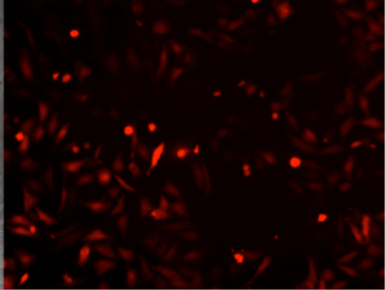

Region 2

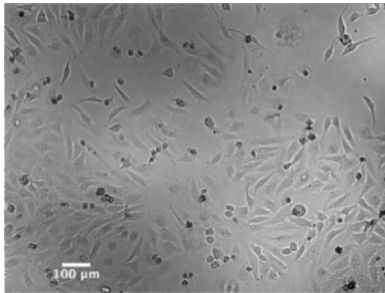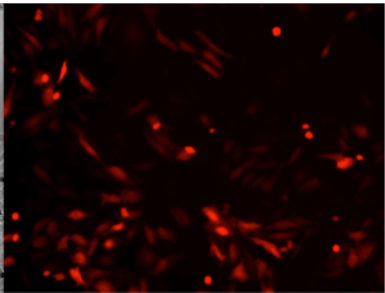

Supplement: Supplementary file 4 [file FBA2-2-264-s004.pdf]

**A**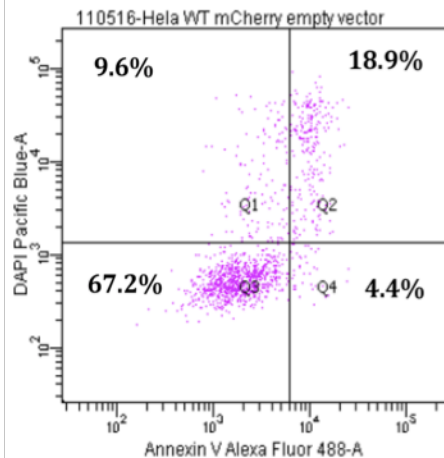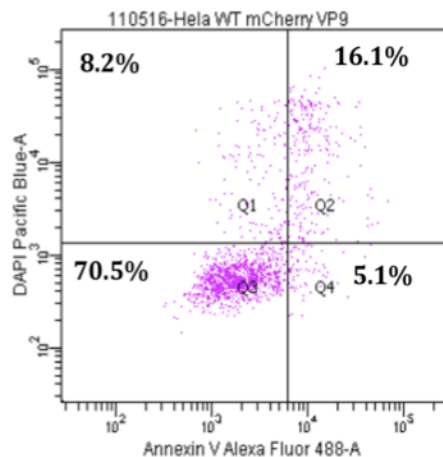**B**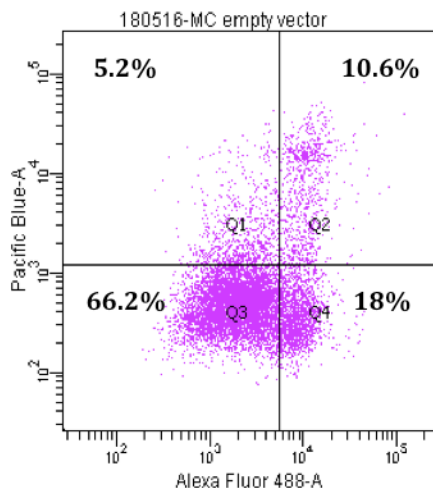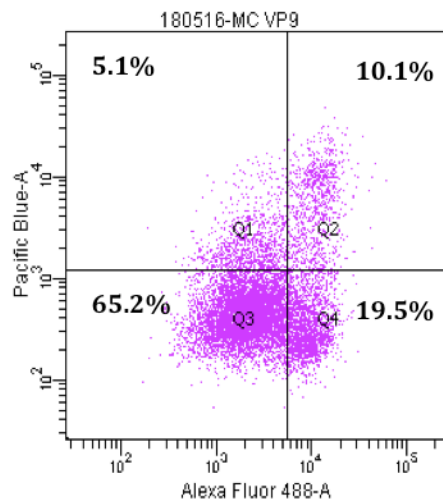

Supplement: Supplementary file 5 [file FBA2-2-264-s005.pdf]

**A**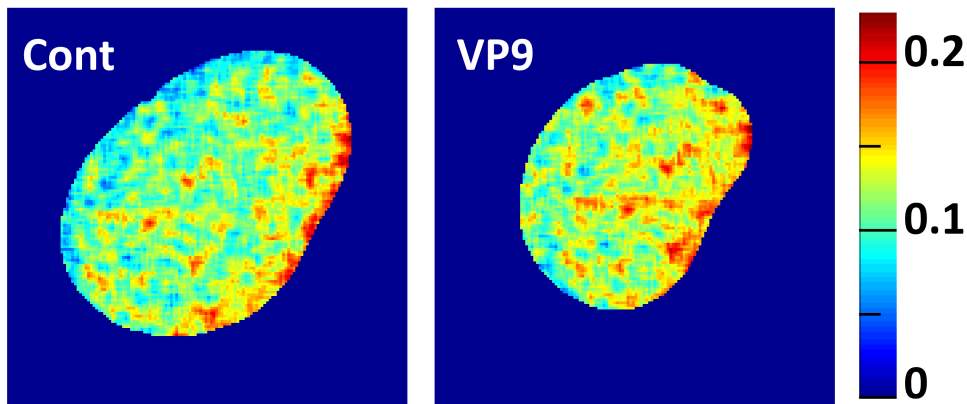**B**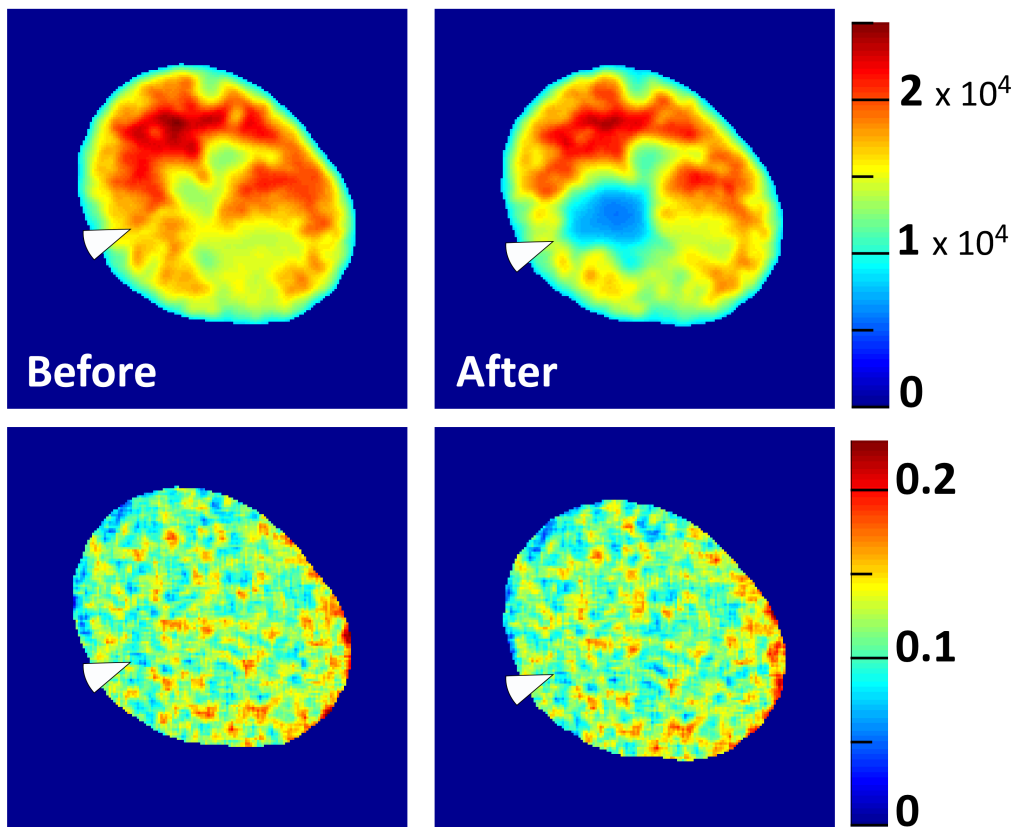

Supplement: Supplementary file 6 [file FBA2-2-264-s006.pdf]

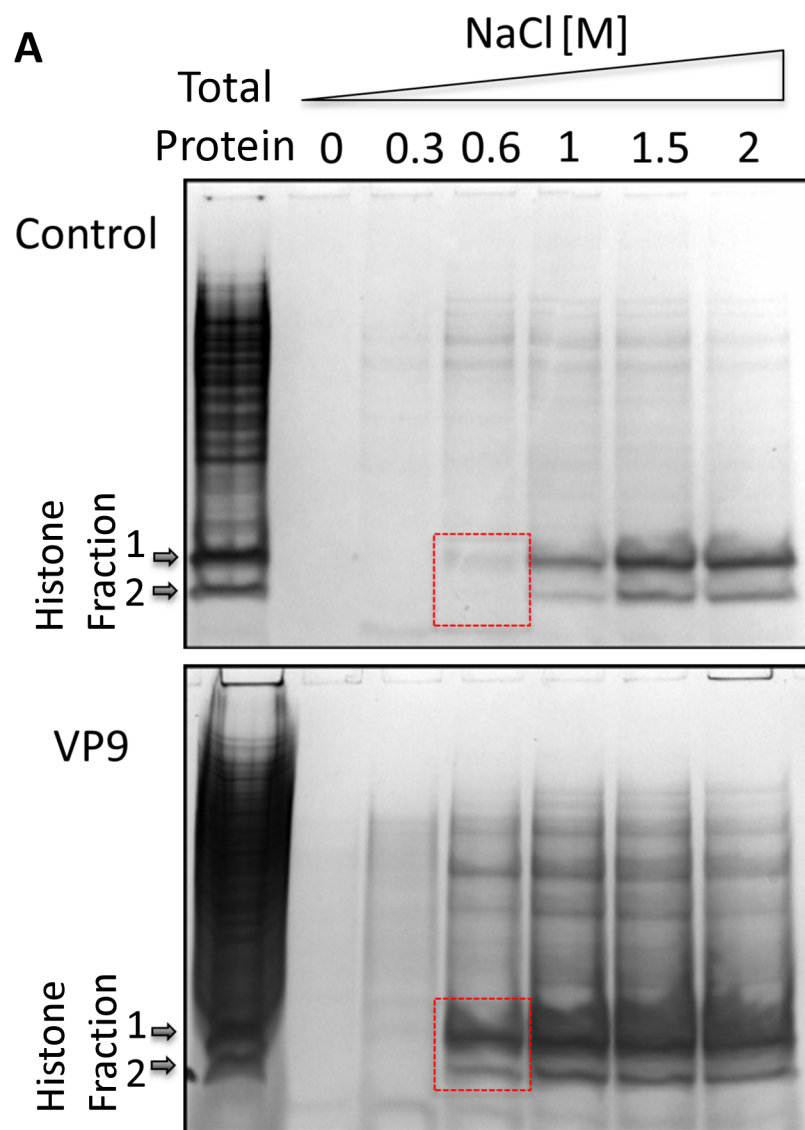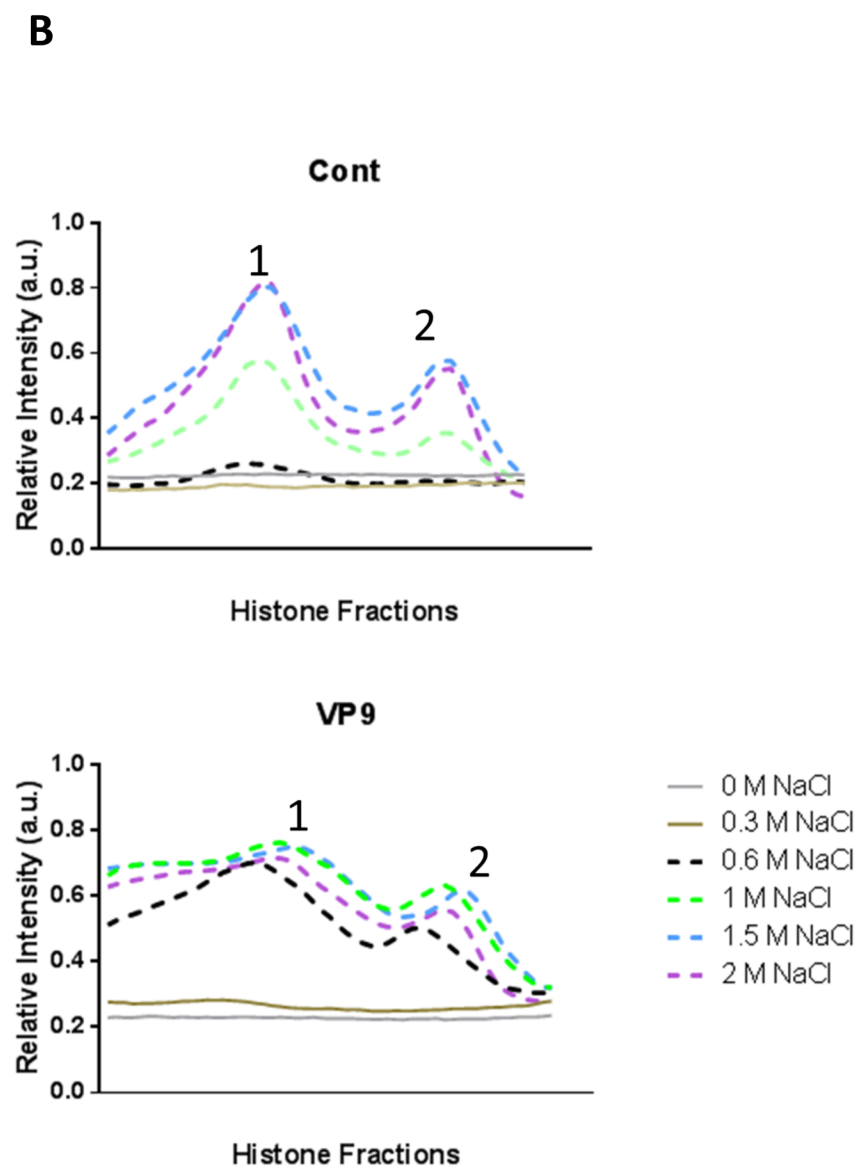

Supplement: Supplementary file 7 [file FBA2-2-264-s007.pdf]

**A**

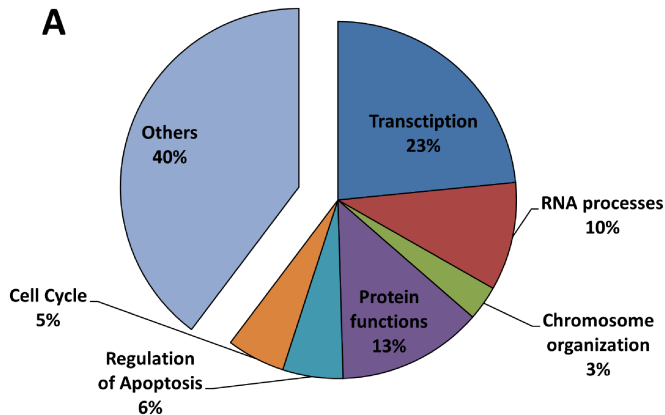

**B**

### DNA and RNA metabolism

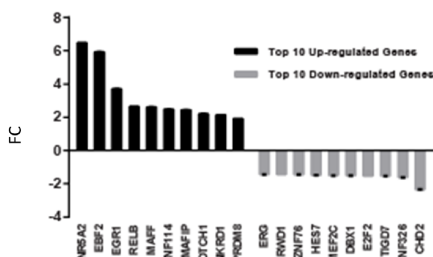

### Cell apoptosis

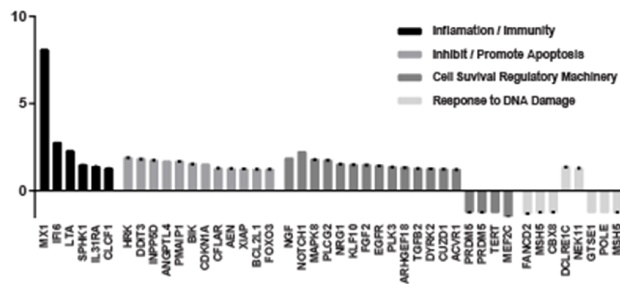

### Nucleosome Organization

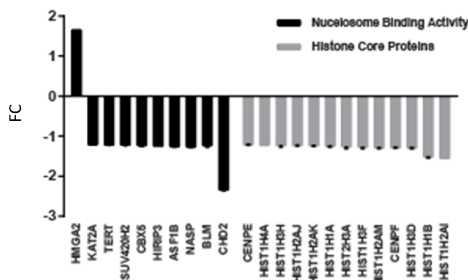

### Cell Cycle

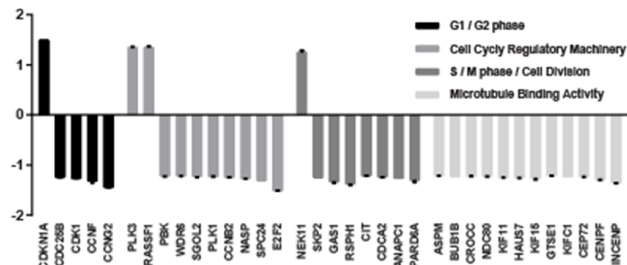

Supplement: Supplementary file 8 [file FBA2-2-264-s008.pdf]
