# Supplementary material for: White spot syndrome viral protein VP9 alters the cellular higher‐order chromatin structure
Source: FASEB Bioadv. 2020 Mar 17;2(4):264–79. doi: 10.1096/fba.2019-00086 (PMC7133739; doi:10.1096/fba.2019-00086)
Supplement: Supplementary file 9 [file FBA2-2-264-s009.docx]

**Table S1. The list of the differential regulation of gene expression upon VP9 expression**

| GeneSymbol | Description | Fold Change | Regulation |
| --- | --- | --- | --- |
| PKP2 | Homo sapiens plakophilin 2 (PKP2), transcript variant 2b, mRNA [NM_004572] | 1.41914 | up |
| LOC391764 | PREDICTED: Homo sapiens putative TAF11-like protein ENSP00000332601-like (LOC391764), mRNA [XM_001713926] | 1.6150459 | down |
| ALDH1A2 | Homo sapiens aldehyde dehydrogenase 1 family, member A2 (ALDH1A2), transcript variant 3, mRNA [NM_170697] | 1.3973216 | down |
| DIS3L2 | Homo sapiens DIS3 mitotic control homolog (S. cerevisiae)-like 2 (DIS3L2), mRNA [NM_152383] | 152.37708 | up |
| MX2 | Homo sapiens myxovirus (influenza virus) resistance 2 (mouse) (MX2), mRNA [NM_002463] | 1.423376 | up |
| PARP12 | Homo sapiens poly (ADP-ribose) polymerase family, member 12 (PARP12), mRNA [NM_022750] | 1.3178442 | up |
| CCRN4L | Homo sapiens CCR4 carbon catabolite repression 4-like (S. cerevisiae) (CCRN4L), mRNA [NM_012118] | 1.6139969 | up |
| CIRBP | Homo sapiens cold inducible RNA binding protein (CIRBP), transcript variant 1, mRNA [NM_001280] | 1.2596532 | down |
| PPM1J | Homo sapiens protein phosphatase, Mg2+/Mn2+ dependent, 1J (PPM1J), mRNA [NM_005167] | 1.2700812 | down |
|  | lincRNA:chr13:52490949-52502949 forward strand | 1.2306985 | down |
| EDN1 | Homo sapiens endothelin 1 (EDN1), transcript variant 1, mRNA [NM_001955] | 1.5972241 | up |
| SLAMF7 | Homo sapiens SLAM family member 7 (SLAMF7), mRNA [NM_021181] | 1.4220196 | up |
| PDZD2 | Homo sapiens PDZ domain containing 2 (PDZD2), mRNA [NM_178140] | 1.2134669 | up |
| EPHA2 | Homo sapiens EPH receptor A2 (EPHA2), mRNA [NM_004431] | 1.5517979 | up |
| AMZ1 | Homo sapiens archaelysin family metallopeptidase 1 (AMZ1), mRNA [NM_133463] | 1.9672248 | up |
| REN | Homo sapiens renin (REN), mRNA [NM_000537] | 1.6567273 | up |
| LOC253805 | Homo sapiens hypothetical protein LOC253805, mRNA (cDNA clone IMAGE:4824622). [BC028622] | 1.447419 | down |
| NPC1L1 | Homo sapiens NPC1 (Niemann-Pick disease, type C1, gene)-like 1 (NPC1L1), transcript variant 1, mRNA [NM_013389] | 1.4259622 | up |
| CDCP1 | Homo sapiens CUB domain containing protein 1 (CDCP1), transcript variant 1, mRNA [NM_022842] | 1.2535913 | up |
| SPRY2 | Homo sapiens sprouty homolog 2 (Drosophila) (SPRY2), mRNA [NM_005842] | 1.2904657 | up |
| HERC5 | Homo sapiens hect domain and RLD 5 (HERC5), mRNA [NM_016323] | 1.5747774 | up |
| OR5I1 | Homo sapiens olfactory receptor, family 5, subfamily I, member 1 (OR5I1), mRNA [NM_006637] | 1.35543 | down |
| SNCA | Homo sapiens synuclein, alpha (non A4 component of amyloid precursor) (SNCA), transcript variant 4, mRNA [NM_007308] | 1.2470508 | down |
| PTPRR | Homo sapiens protein tyrosine phosphatase, receptor type, R (PTPRR), transcript variant 1, mRNA [NM_002849] | 1.595302 | up |
|  | lincRNA:chr2:139225080-139239905 reverse strand | 1.8760788 | down |
| PLCG2 | Homo sapiens phospholipase C, gamma 2 (phosphatidylinositol-specific) (PLCG2), mRNA [NM_002661] | 1.7291832 | up |
|  | lincRNA:chr9:138999696-139000927 reverse strand | 1.2079631 | up |
| NFKB2 | Homo sapiens nuclear factor of kappa light polypeptide gene enhancer in B-cells 2 (p49/p100) (NFKB2), transcript variant 3, mRNA [NM_001077493] | 1.3038856 | up |
| WDR26 | Homo sapiens WD repeat domain 26 (WDR26), transcript variant 1, mRNA [NM_025160] | 1.2390693 | up |
| NCF1 | Homo sapiens neutrophil cytosolic factor 1 (NCF1), mRNA [NM_000265] | 100.97418 | up |
|  | lincRNA:chr5:44816593-44823034 forward strand | 1.2865502 | down |
| LCE2D | Homo sapiens late cornified envelope 2D (LCE2D), mRNA [NM_178430] | 87.534676 | up |
| TRMT1L | Homo sapiens TRM1 tRNA methyltransferase 1-like (TRMT1L), transcript variant 1, mRNA [NM_030934] | 1.257339 | down |
| AR | Homo sapiens androgen receptor (AR), transcript variant 1, mRNA [NM_000044] | 1.2894131 | down |
| AKAP1 | Homo sapiens A kinase (PRKA) anchor protein 1 (AKAP1), nuclear gene encoding mitochondrial protein, transcript variant 1, mRNA [NM_003488] | 1.2223836 | down |
| FLJ43663 | Homo sapiens uncharacterized LOC378805 (FLJ43663), transcript variant 1, non-coding RNA [NR_015431] | 1.2194973 | up |
| GAS1 | Homo sapiens growth arrest-specific 1 (GAS1), mRNA [NM_002048] | 1.3207618 | down |
| C11orf86 | Homo sapiens chromosome 11 open reading frame 86 (C11orf86), mRNA [NM_001136485] | 1.332629 | down |
| OMA1 | Homo sapiens OMA1 homolog, zinc metallopeptidase (S. cerevisiae) (OMA1), mRNA [NM_145243] | 1.264486 | down |
| ZFAND4 | Homo sapiens AN1, ubiquitin-like, homolog (Xenopus laevis) (ANUBL1), transcript variant 1, mRNA [NM_174890] | 1.2030641 | down |
| HAS2 | Homo sapiens hyaluronan synthase 2 (HAS2), mRNA [NM_005328] | 1.25958 | up |
| ADAT2 | Homo sapiens adenosine deaminase, tRNA-specific 2 (ADAT2), mRNA [NM_182503] | 1.2043434 | down |
| PLA2G4A | Homo sapiens phospholipase A2, group IVA (cytosolic, calcium-dependent) (PLA2G4A), mRNA [NM_024420] | 1.2319317 | down |
| TBL1Y | Homo sapiens transducin (beta)-like 1, Y-linked (TBL1Y), transcript variant 1, mRNA [NM_033284] | 1.2241524 | down |
| ASF1B | Homo sapiens ASF1 anti-silencing function 1 homolog B (S. cerevisiae) (ASF1B), mRNA [NM_018154] | 1.245836 | down |
|  | tripartite motif containing 16 [Source:HGNC Symbol;Acc:17241] [ENST00000494759] | 1.2401744 | down |
|  | lincRNA:chr4:23771777-23778877 reverse strand | 1.3363919 | up |
| PLAU | Homo sapiens plasminogen activator, urokinase (PLAU), transcript variant 1, mRNA [NM_002658] | 1.4801782 | up |
| ELL3 | Homo sapiens elongation factor RNA polymerase II-like 3 (ELL3), mRNA [NM_025165] | 1.2236211 | down |
| RASAL2 | Homo sapiens RAS protein activator like 2 (RASAL2), transcript variant 2, mRNA [NM_170692] | 1.2904543 | up |
|  | lincRNA:chrX:135994109-136086359 forward strand | 1.4875612 | down |
| GPER | Homo sapiens G protein-coupled estrogen receptor 1 (GPER), transcript variant 3, mRNA [NM_001039966] | 1.328983 | up |
| ERAP1 | Homo sapiens endoplasmic reticulum aminopeptidase 1 (ERAP1), transcript variant 2, mRNA [NM_001040458] | 1.2839193 | up |
| YPEL5 | Homo sapiens yippee-like 5 (Drosophila) (YPEL5), transcript variant 4, mRNA [NM_016061] | 1.2667655 | up |
| GBP1P1 | Homo sapiens guanylate binding protein 1, interferon-inducible pseudogene 1 (GBP1P1), non-coding RNA [NR_003133] | 86.53044 | up |
|  | lincRNA:chr7:131337972-131438914 forward strand | 1.3140681 | down |
| ARG2 | Homo sapiens arginase, type II (ARG2), nuclear gene encoding mitochondrial protein, mRNA [NM_001172] | 1.3327695 | down |
| EGFR | Homo sapiens epidermal growth factor receptor (EGFR), transcript variant 1, mRNA [NM_005228] | 1.4153833 | up |
| DOCK5 | Homo sapiens dedicator of cytokinesis 5 (DOCK5), mRNA [NM_024940] | 1.2962822 | up |
| GRB10 | Homo sapiens growth factor receptor-bound protein 10 (GRB10), transcript variant 4, mRNA [NM_001001555] | 1.2071909 | up |
| LRRC8C | Homo sapiens leucine rich repeat containing 8 family, member C (LRRC8C), mRNA [NM_032270] | 1.4615285 | up |
| DDIT4 | Homo sapiens DNA-damage-inducible transcript 4 (DDIT4), mRNA [NM_019058] | 1.4626784 | down |
| PRKAG3 | Homo sapiens protein kinase, AMP-activated, gamma 3 non-catalytic subunit (PRKAG3), mRNA [NM_017431] | 1.4227308 | up |
| TMEM37 | Homo sapiens transmembrane protein 37 (TMEM37), mRNA [NM_183240] | 1.2011477 | down |
| ZNF185 | Homo sapiens zinc finger protein 185 (LIM domain) (ZNF185), transcript variant 1, mRNA [NM_001178106] | 1.2839414 | up |
| PLA2G4F | Homo sapiens phospholipase A2, group IVF (PLA2G4F), transcript variant 1, mRNA [NM_213600] | 59.09128 | up |
| MFAP3L | Homo sapiens microfibrillar-associated protein 3-like (MFAP3L), transcript variant 1, mRNA [NM_021647] | 1.2480688 | up |
| MMAB | Homo sapiens methylmalonic aciduria (cobalamin deficiency) cblB type (MMAB), nuclear gene encoding mitochondrial protein, transcript variant 1, mRNA [NM_052845] | 1.487791 | down |
| PRRT3 | Homo sapiens proline-rich transmembrane protein 3 (PRRT3), mRNA [NM_207351] | 1.2031226 | down |
| PCSK1 | Homo sapiens proprotein convertase subtilisin/kexin type 1 (PCSK1), transcript variant 1, mRNA [NM_000439] | 1.2833052 | down |
| PDGFA | Homo sapiens platelet-derived growth factor alpha polypeptide (PDGFA), transcript variant 1, mRNA [NM_002607] | 1.3757068 | up |
| CHRM2 | Homo sapiens cholinergic receptor, muscarinic 2 (CHRM2), transcript variant 1, mRNA [NM_001006630] | 1.2338772 | down |
| LOC440131 | Homo sapiens uncharacterized LOC440131 (LOC440131), non-coding RNA [NR_033889] | 1.225747 | down |
| MSMO1 | Homo sapiens methylsterol monooxygenase 1 (MSMO1), transcript variant 1, mRNA [NM_006745] | 1.2182277 | up |
| E2F2 | Homo sapiens E2F transcription factor 2 (E2F2), mRNA [NM_004091] | 1.5012331 | down |
| ASB13 | Homo sapiens ankyrin repeat and SOCS box containing 13 (ASB13), transcript variant 1, mRNA [NM_024701] | 1.2811493 | down |
|  | lincRNA:chr19:56807436-56826208 reverse strand | 1.44005 | up |
| NGF | Homo sapiens nerve growth factor (beta polypeptide) (NGF), mRNA [NM_002506] | 1.8352522 | up |
| ZNF295 | Homo sapiens zinc finger protein 295 (ZNF295), transcript variant 1, mRNA [NM_001098402] | 1.2571146 | up |
| ARHGEF12 | Homo sapiens Rho guanine nucleotide exchange factor (GEF) 12 (ARHGEF12), transcript variant 1, mRNA [NM_015313] | 1.214893 | down |
| RSPH1 | Homo sapiens radial spoke head 1 homolog (Chlamydomonas) (RSPH1), mRNA [NM_080860] | 1.3654355 | down |
| LOC100129104 | PREDICTED: Homo sapiens hypothetical LOC100129104 (LOC100129104), miscRNA [XR_108889] | 1.6999253 | up |
| HIVEP1 | Homo sapiens human immunodeficiency virus type I enhancer binding protein 1 (HIVEP1), mRNA [NM_002114] | 1.2695861 | up |
| ZSWIM5 | Homo sapiens zinc finger, SWIM-type containing 5 (ZSWIM5), mRNA [NM_020883] | 1.2598675 | down |
| KIF20A | Homo sapiens kinesin family member 20A (KIF20A), mRNA [NM_005733] | 1.3533505 | down |
| ANGPT1 | Homo sapiens angiopoietin 1 (ANGPT1), transcript variant 1, mRNA [NM_001146] | 1.4090086 | down |
|  |  | 1.9487876 | down |
| CTGF | Homo sapiens connective tissue growth factor (CTGF), mRNA [NM_001901] | 1.2667496 | up |
| CUZD1 | Homo sapiens CUB and zona pellucida-like domains 1 (CUZD1), transcript variant 1, mRNA [NM_022034] | 1.2174797 | up |
| KIAA0586 | Homo sapiens KIAA0586 (KIAA0586), transcript variant 5, mRNA [NM_014749] | 1.2031473 | down |
| CNTNAP3B | contactin associated protein-like 3B [Source:HGNC Symbol;Acc:32035] [ENST00000276974] | 1.2169347 | down |
| CENPE | Homo sapiens centromere protein E, 312kDa (CENPE), mRNA [NM_001813] | 1.20776 | down |
|  | lincRNA:chr4:53577368-53616143 reverse strand | 1.2416335 | down |
| HIST2H3A | Homo sapiens histone cluster 2, H3a (HIST2H3A), mRNA [NM_001005464] | 1.2523575 | down |
| OR5AK4P | Homo sapiens olfactory-like receptor (PJCG3) pseudogene, mRNA sequence. [AF309700] | 1.2439032 | down |
| LOC285696 | Homo sapiens cDNA FLJ43202 fis, clone FEBRA2008360. [AK125192] | 1.3525014 | down |
| CXCR3 | Homo sapiens chemokine (C-X-C motif) receptor 3 (CXCR3), transcript variant 1, mRNA [NM_001504] | 1.7714849 | up |
| MCOLN1 | Homo sapiens mucolipin 1 (MCOLN1), mRNA [NM_020533] | 1.2412459 | up |
| ASAP2 | Homo sapiens ArfGAP with SH3 domain, ankyrin repeat and PH domain 2 (ASAP2), transcript variant 1, mRNA [NM_003887] | 1.2194208 | up |
| SLCO2A1 | Homo sapiens solute carrier organic anion transporter family, member 2A1 (SLCO2A1), mRNA [NM_005630] | 1.2870678 | up |
| LINC00473 | Homo sapiens long intergenic non-protein coding RNA 473 (LINC00473), transcript variant 1, non-coding RNA [NR_026860] | 1.5182707 | down |
| TAF15 | Homo sapiens TAF15 RNA polymerase II, TATA box binding protein (TBP)-associated factor, 68kDa (TAF15), transcript variant 1, mRNA [NM_139215] | 1.252604 | down |
|  |  | 1.2031611 | up |
| TERT | Homo sapiens telomerase reverse transcriptase (TERT), transcript variant 1, mRNA [NM_198253] | 1.2048038 | down |
| NTNG1 | Homo sapiens netrin G1 (NTNG1), transcript variant 3, mRNA [NM_014917] | 1.3591341 | down |
|  | 603252951F1 NIH_MGC_97 Homo sapiens cDNA clone IMAGE:5295349 5', mRNA sequence [BI559448] | 28.884754 | up |
| SCLY | Homo sapiens selenocysteine lyase (SCLY), mRNA [NM_016510] | 1.2137613 | down |
| XKRX | Homo sapiens XK, Kell blood group complex subunit-related, X-linked (XKRX), mRNA [NM_212559] | 1.2392032 | up |
|  | lincRNA:chr10:118588285-118590480 reverse strand | 1.221337 | down |
| C1QTNF3 | Homo sapiens C1q and tumor necrosis factor related protein 3 (C1QTNF3), transcript variant 2, mRNA [NM_181435] | 1.3211688 | down |
| OSBPL3 | Homo sapiens oxysterol binding protein-like 3 (OSBPL3), transcript variant 1, mRNA [NM_015550] | 1.3096077 | up |
|  | lincRNA:chr12:12884108-12894958 forward strand | 1.2384365 | down |
| FAM196B | Homo sapiens family with sequence similarity 196, member B (FAM196B), mRNA [NM_001129891] | 1.525867 | up |
| LOC392196 | Homo sapiens ubiquitin carboxyl-terminal hydrolase 17-like pseudogene (LOC392196), non-coding RNA [NR_003275] | 1.3348778 | up |
| FFAR3 | Homo sapiens free fatty acid receptor 3 (FFAR3), mRNA [NM_005304] | 1.4424264 | up |
|  | lincRNA:chr13:44760050-44767325 reverse strand | 1.4637454 | down |
| ITPRIP | Homo sapiens inositol 1,4,5-trisphosphate receptor interacting protein (ITPRIP), mRNA [NM_033397] | 1.4481363 | up |
| PLCB4 | Homo sapiens phospholipase C, beta 4 (PLCB4), transcript variant 3, mRNA [NM_001172646] | 1.204926 | up |
|  | lincRNA:chr3:160981722-160983945 forward strand | 27.942644 | up |
| STX3 | Homo sapiens syntaxin 3 (STX3), transcript variant 1, mRNA [NM_004177] | 1.3501537 | up |
| TLR3 | Homo sapiens toll-like receptor 3 (TLR3), mRNA [NM_003265] | 1.2939495 | up |
|  | lincRNA:chr4:15135577-15211177 forward strand | 1.343798 | down |
|  | lincRNA:chr8:28543756-28558056 forward strand | 1.2381225 | down |
| PRDM5 | Homo sapiens PR domain containing 5 (PRDM5), mRNA [NM_018699] | 1.2009677 | down |
| KCTD12 | Homo sapiens potassium channel tetramerisation domain containing 12 (KCTD12), mRNA [NM_138444] | 1.2701592 | down |
|  | lincRNA:chr6:14706771-14748171 reverse strand | 21.446869 | up |
| CACNA2D3 | Homo sapiens calcium channel, voltage-dependent, alpha 2/delta subunit 3 (CACNA2D3), mRNA [NM_018398] | 1.3616723 | down |
| BRI3BP | Homo sapiens BRI3 binding protein (BRI3BP), mRNA [NM_080626] | 1.3749969 | down |
| TRIM54 | Homo sapiens tripartite motif containing 54 (TRIM54), transcript variant 1, mRNA [NM_032546] | 1.4157821 | up |
| NFKBIE | Homo sapiens nuclear factor of kappa light polypeptide gene enhancer in B-cells inhibitor, epsilon (NFKBIE), mRNA [NM_004556] | 1.2233729 | up |
| DIO2 | Homo sapiens deiodinase, iodothyronine, type II (DIO2), transcript variant 1, mRNA [NM_013989] | 1.2517 | down |
|  | lincRNA:chr10:80202694-80215169 reverse strand | 1.5079373 | up |
| KLHL30 | Homo sapiens kelch-like 30 (Drosophila) (KLHL30), mRNA [NM_198582] | 1.6120353 | up |
| LPAR5 | Homo sapiens lysophosphatidic acid receptor 5 (LPAR5), transcript variant 1, mRNA [NM_020400] | 1.8499231 | up |
| TIGD7 | Homo sapiens tigger transposable element derived 7 (TIGD7), mRNA [NM_033208] | 1.5140693 | down |
| DCLK1 | Homo sapiens doublecortin-like kinase 1 (DCLK1), transcript variant 1, mRNA [NM_004734] | 1.2722827 | up |
| KRT34 | Homo sapiens keratin 34 (KRT34), mRNA [NM_021013] | 1.9973203 | up |
| BIK | Homo sapiens BCL2-interacting killer (apoptosis-inducing) (BIK), mRNA [NM_001197] | 1.5234232 | up |
| LOC100507206 | Homo sapiens uncharacterized LOC100507206 (LOC100507206), non-coding RNA [NR_038256] | 17.03735 | up |
| FLG | Homo sapiens filaggrin (FLG), mRNA [NM_002016] | 15.421414 | up |
| LOC100506190 | Homo sapiens uncharacterized LOC100506190 (LOC100506190), non-coding RNA [NR_038955] | 1.269559 | up |
|  | lincRNA:chr3:129602510-129611708 reverse strand | 1.2130597 | down |
| FXYD7 | Homo sapiens FXYD domain containing ion transport regulator 7 (FXYD7), mRNA [NM_022006] | 1.6193036 | down |
| KLF9 | Homo sapiens Kruppel-like factor 9 (KLF9), mRNA [NM_001206] | 1.282553 | down |
| TWIST2 | Homo sapiens twist homolog 2 (Drosophila), mRNA (cDNA clone MGC:117334 IMAGE:6021554), complete cds. [BC103755] | 1.2343515 | down |
| C9orf93 | Homo sapiens chromosome 9 open reading frame 93 (C9orf93), mRNA [NM_173550] | 1.3494716 | down |
| XLOC_012826 | BROAD Institute lincRNA (XLOC_012826), lincRNA [TCONS_00026204] | 1.3868102 | down |
| CLCN4 | Homo sapiens chloride channel 4 (CLCN4), mRNA [NM_001830] | 1.2358524 | up |
| MAP2K6 | Homo sapiens mitogen-activated protein kinase kinase 6 (MAP2K6), mRNA [NM_002758] | 1.3718182 | down |
| CIT | Homo sapiens citron (rho-interacting, serine/threonine kinase 21) (CIT), transcript variant 2, mRNA [NM_007174] | 1.2012309 | down |
| ASB2 | Homo sapiens ankyrin repeat and SOCS box containing 2 (ASB2), transcript variant 2, mRNA [NM_016150] | 1.4390107 | up |
| RIMKLB | Homo sapiens ribosomal modification protein rimK-like family member B (RIMKLB), mRNA [NM_020734] | 1.4430546 | up |
|  | lincRNA:chr21:44014781-44021030 forward strand | 1.3008549 | down |
| LOC100128160 | PREDICTED: Homo sapiens hypothetical LOC100128160 (LOC100128160), mRNA [XM_001714107] | 14.508068 | up |
| TMEM57 | Homo sapiens transmembrane protein 57 (TMEM57), mRNA [NM_018202] | 1.266009 | up |
| TMEM57 | Homo sapiens transmembrane protein 57 (TMEM57), mRNA [NM_018202] | 1.2941006 | up |
| OR1F2P | Homo sapiens olfactory receptor, family 1, subfamily F, member 2 (OR1F2P), non-coding RNA [NR_002169] | 12.160087 | up |
| ZNF587 | Homo sapiens zinc finger protein 587, mRNA (cDNA clone IMAGE:4153939), with apparent retained intron. [BC011243] | 1.2321274 | down |
| GPR87 | Homo sapiens G protein-coupled receptor 87 (GPR87), mRNA [NM_023915] | 1.5358706 | up |
| FAM81A | Homo sapiens family with sequence similarity 81, member A (FAM81A), mRNA [NM_152450] | 1.2533891 | down |
| TMC6 | Homo sapiens transmembrane channel-like 6 (TMC6), transcript variant 2, mRNA [NM_007267] | 1.2622344 | up |
| ATRIP | Homo sapiens ATR interacting protein (ATRIP), transcript variant 2, mRNA [NM_032166] | 1.2190479 | down |
| G3BP1 | GTPase activating protein (SH3 domain) binding protein 1 [Source:HGNC Symbol;Acc:30292] [ENST00000394123] | 1.2234688 | down |
| C3orf51 | Homo sapiens chromosome 3 open reading frame 51 (C3orf51), non-coding RNA [NR_024615] | 1.2534769 | down |
| NELL2 | Homo sapiens NEL-like 2 (chicken) (NELL2), transcript variant 2, mRNA [NM_006159] | 1.3226848 | down |
| CCDC74B | Homo sapiens coiled-coil domain containing 74B (CCDC74B), mRNA [NM_207310] | 1.2697148 | down |
| GFPT1 | Homo sapiens glutamine--fructose-6-phosphate transaminase 1 (GFPT1), transcript variant 1, mRNA [NM_001244710] | 1.2223516 | up |
| OLFML2B | Homo sapiens olfactomedin-like 2B (OLFML2B), mRNA [NM_015441] | 10.095885 | up |
| LTA4H | Homo sapiens leukotriene A4 hydrolase (LTA4H), mRNA [NM_000895] | 1.9344501 | down |
|  |  | 1.2467074 | down |
| FAM189A2 | Homo sapiens family with sequence similarity 189, member A2 (FAM189A2), transcript variant 1, mRNA [NM_004816] | 1.3627884 | up |
|  | lincRNA:chr5:72707719-72723794 reverse strand | 1.2780436 | up |
| ANGPTL5 | Homo sapiens angiopoietin-like 5 (ANGPTL5), mRNA [NM_178127] | 9.803392 | up |
| HIST1H2AM | Homo sapiens histone cluster 1, H2am (HIST1H2AM), mRNA [NM_003514] | 1.2804301 | down |
| HIST1H1D | Homo sapiens histone cluster 1, H1d (HIST1H1D), mRNA [NM_005320] | 1.3386854 | down |
|  | PREDICTED: Homo sapiens hypothetical LOC100127974 (LOC100127974), miscRNA [XR_109126] | 9.522657 | up |
| CCL5 | Homo sapiens chemokine (C-C motif) ligand 5 (CCL5), mRNA [NM_002985] | 9.0436945 | up |
| IMPA2 | Homo sapiens inositol(myo)-1(or 4)-monophosphatase 2 (IMPA2), mRNA [NM_014214] | 1.3160658 | down |
| MSH5 | Homo sapiens mutS homolog 5 (E. coli) (MSH5), transcript variant 3, mRNA [NM_002441] | 1.231561 | down |
| LDB3 | Homo sapiens LIM domain binding 3 (LDB3), transcript variant 5, mRNA [NM_001171610] | 1.7256662 | up |
| ASGR1 | Homo sapiens asialoglycoprotein receptor 1 (ASGR1), transcript variant 1, mRNA [NM_001671] | 1.342751 | up |
| KBTBD7 | Homo sapiens kelch repeat and BTB (POZ) domain containing 7 (KBTBD7), mRNA [NM_032138] | 1.2371567 | down |
| PLK1 | Homo sapiens polo-like kinase 1 (PLK1), mRNA [NM_005030] | 1.2215021 | down |
|  | lincRNA:chr6:147189982-147499757 reverse strand | 1.3324043 | down |
| ZFP36L2 | Homo sapiens zinc finger protein 36, C3H type-like 2 (ZFP36L2), mRNA [NM_006887] | 1.2904272 | up |
| TUB | Homo sapiens tubby homolog (mouse) (TUB), transcript variant 1, mRNA [NM_003320] | 1.2476768 | down |
| PLEKHO1 | Homo sapiens pleckstrin homology domain containing, family O member 1 (PLEKHO1), mRNA [NM_016274] | 1.3539096 | up |
| LOC100130348 | Uncharacterized protein [Source:UniProtKB/TrEMBL;Acc:E9PME8] [ENST00000528029] | 8.46929 | up |
| CAMK2N1 | Homo sapiens calcium/calmodulin-dependent protein kinase II inhibitor 1 (CAMK2N1), mRNA [NM_018584] | 1.5606071 | up |
| GSTA4 | Homo sapiens glutathione S-transferase alpha 4 (GSTA4), mRNA [NM_001512] | 1.2048079 | down |
| ABL2 | Homo sapiens v-abl Abelson murine leukemia viral oncogene homolog 2 (ABL2), transcript variant b, mRNA [NM_007314] | 1.5002213 | up |
| PTGES | Homo sapiens prostaglandin E synthase (PTGES), mRNA [NM_004878] | 1.2692086 | up |
| MAGEE1 | Homo sapiens melanoma antigen family E, 1 (MAGEE1), mRNA [NM_020932] | 1.2857838 | down |
| WIPF1 | Homo sapiens WAS/WASL interacting protein family, member 1, mRNA (cDNA clone MGC:111041 IMAGE:5184105), complete cds. [BC110288] | 1.2330588 | down |
| MYOM1 | Homo sapiens myomesin 1, 185kDa (MYOM1), transcript variant 1, mRNA [NM_003803] | 1.3505435 | down |
| SKIL | Homo sapiens SKI-like oncogene (SKIL), transcript variant 1, mRNA [NM_005414] | 1.2951645 | up |
| FUT1 | Homo sapiens fucosyltransferase 1 (galactoside 2-alpha-L-fucosyltransferase, H blood group) (FUT1), mRNA [NM_000148] | 1.3159684 | up |
| ARRDC2 | Homo sapiens arrestin domain containing 2 (ARRDC2), transcript variant 2, mRNA [NM_001025604] | 1.2019 | up |
| RTN4IP1 | Homo sapiens reticulon 4 interacting protein 1 (RTN4IP1), nuclear gene encoding mitochondrial protein, mRNA [NM_032730] | 1.2072775 | down |
| STXBP6 | Homo sapiens syntaxin binding protein 6 (amisyn) (STXBP6), mRNA [NM_014178] | 1.3511322 | up |
| ELK1 | Homo sapiens ELK1, member of ETS oncogene family (ELK1), transcript variant 2, mRNA [NM_005229] | 1.2065153 | down |
| MX1 | Homo sapiens myxovirus (influenza virus) resistance 1, interferon-inducible protein p78 (mouse) (MX1), transcript variant 2, mRNA [NM_002462] | 8.0683 | up |
| LOC100287765 | Homo sapiens uncharacterized LOC100287765 (LOC100287765), non-coding RNA [NR_038988] | 1.2398375 | up |
|  | lincRNA:chr7:17018702-17092594 forward strand | 7.941562 | up |
| STX1A | Homo sapiens syntaxin 1A (brain) (STX1A), transcript variant 1, mRNA [NM_004603] | 1.3385193 | up |
|  | lincRNA:chr8:114487974-115096649 forward strand | 1.2310448 | up |
| KCNJ12 | Homo sapiens potassium inwardly-rectifying channel, subfamily J, member 12 (KCNJ12), mRNA [NM_021012] | 1.5692387 | up |
| CDK1 | Homo sapiens cyclin-dependent kinase 1 (CDK1), transcript variant 1, mRNA [NM_001786] | 1.2583376 | down |
| CDAN1 | Homo sapiens congenital dyserythropoietic anemia, type I (CDAN1), mRNA [NM_138477] | 1.2149539 | down |
| FNIP1 | Homo sapiens folliculin interacting protein 1 (FNIP1), transcript variant 1, mRNA [NM_133372] | 1.3104929 | up |
|  |  | 1.5115868 | down |
|  | lincRNA:chr7:30551275-30562550 forward strand | 1.3333972 | up |
| RCCD1 | Homo sapiens RCC1 domain containing 1 (RCCD1), transcript variant 1, mRNA [NM_033544] | 1.2251433 | down |
| HES7 | Homo sapiens hairy and enhancer of split 7 (Drosophila) (HES7), transcript variant 1, mRNA [NM_001165967] | 1.4667103 | down |
| RASSF1 | Homo sapiens Ras association (RalGDS/AF-6) domain family member 1 (RASSF1), transcript variant C, mRNA [NM_170713] | 1.3406322 | up |
| NXF2 | Homo sapiens nuclear RNA export factor 2 (NXF2), mRNA [NM_022053] | 7.81558 | up |
| C5 | Homo sapiens complement component 5 (C5), mRNA [NM_001735] | 1.2950246 | down |
| SYNGR1 | Homo sapiens synaptogyrin 1 (SYNGR1), transcript variant 1c, mRNA [NM_145738] | 1.3167876 | down |
| PLEKHO2 | Homo sapiens pleckstrin homology domain containing, family O member 2 (PLEKHO2), transcript variant 1, mRNA [NM_025201] | 1.2338845 | up |
| OR6W1P | Homo sapiens olfactory receptor, family 6, subfamily W, member 1 pseudogene (OR6W1P), non-coding RNA [NR_002140] | 7.505643 | up |
| SAT1 | Homo sapiens spermidine/spermine N1-acetyltransferase 1 (SAT1), transcript variant 1, mRNA [NM_002970] | 1.2510787 | up |
|  | lincRNA:chr15:69951596-69988646 forward strand | 6.510302 | up |
| S100A7 | Homo sapiens S100 calcium binding protein A7 (S100A7), mRNA [NM_002963] | 1.2937791 | up |
| SHOX2 | Homo sapiens short stature homeobox 2 (SHOX2), transcript variant 1, mRNA [NM_003030] | 1.3630332 | up |
| LOC100506190 |  | 1.224792 | down |
| LOC100132356 | Homo sapiens uncharacterized LOC100132356 (LOC100132356), non-coding RNA [NR_034127] | 1.2070063 | up |
|  | lincRNA:chr6:155203224-155205268 forward strand | 1.2255412 | down |
| DBP | Homo sapiens D site of albumin promoter (albumin D-box) binding protein (DBP), mRNA [NM_001352] | 1.3214495 | down |
|  | lincRNA:chr5:44826637-44828692 forward strand | 1.3005754 | down |
| HYDIN2 | PREDICTED: Homo sapiens hydrocephalus inducing homolog 2 (mouse) (HYDIN2), miscRNA [XR_133104] | 1.5657394 | down |
| SLC25A34 | Homo sapiens solute carrier family 25, member 34 (SLC25A34), mRNA [NM_207348] | 1.5416662 | up |
|  | PREDICTED: Homo sapiens hypothetical LOC100506936, transcript variant 1 (LOC100506936), miscRNA [XR_108774] | 1.363991 | up |
| SPRR2E | Homo sapiens small proline-rich protein 2E (SPRR2E), mRNA [NM_001024209] | 1.3408015 | down |
| INCENP | Homo sapiens inner centromere protein antigens 135/155kDa (INCENP), transcript variant 1, mRNA [NM_001040694] | 1.3417151 | down |
| SLC12A3 | Homo sapiens solute carrier family 12 (sodium/chloride transporters), member 3 (SLC12A3), transcript variant 1, mRNA [NM_000339] | 1.3345475 | down |
| CHIC2 | Homo sapiens cysteine-rich hydrophobic domain 2 (CHIC2), mRNA [NM_012110] | 1.316024 | up |
| MACF1 | Homo sapiens microtubule-actin crosslinking factor 1 (MACF1), transcript variant 1, mRNA [NM_012090] | 1.3445212 | up |
| KIFC1 | Homo sapiens kinesin family member C1 (KIFC1), mRNA [NM_002263] | 1.2206526 | down |
|  | lincRNA:chr14:101389472-101539847 forward strand | 1.5462509 | up |
| NFATC1 | Homo sapiens nuclear factor of activated T-cells, cytoplasmic, calcineurin-dependent 1 (NFATC1), transcript variant 1, mRNA [NM_172390] | 1.4060198 | up |
|  | lincRNA:chr7:55526931-55538319 reverse strand | 1.4640131 | down |
| LOC100289509 | Homo sapiens uncharacterized LOC100289509 (LOC100289509), non-coding RNA [NR_045118] | 1.5103061 | down |
| SPC24 | Homo sapiens cDNA FLJ90806 fis, clone Y79AA1000750. [AK075287] | 1.296951 | down |
| MGLL | Homo sapiens monoglyceride lipase (MGLL), transcript variant 1, mRNA [NM_007283] | 1.2511185 | up |
|  | ALU6_HUMAN (P39193) Alu subfamily SP sequence contamination warning entry, partial (18%) [THC2521288] | 1.3626136 | up |
| SUOX | Homo sapiens sulfite oxidase (SUOX), nuclear gene encoding mitochondrial protein, transcript variant 1, mRNA [NM_000456] | 1.2022558 | down |
| NR5A2 | Homo sapiens nuclear receptor subfamily 5, group A, member 2 (NR5A2), transcript variant 1, mRNA [NM_205860] | 6.474522 | up |
| TUFT1 | Homo sapiens tuftelin 1 (TUFT1), transcript variant 1, mRNA [NM_020127] | 1.4282084 | up |
| F3 | Homo sapiens coagulation factor III (thromboplastin, tissue factor) (F3), transcript variant 1, mRNA [NM_001993] | 1.5013429 | up |
| CTSC | Homo sapiens cathepsin C (CTSC), transcript variant 2, mRNA [NM_148170] | 1.2393275 | down |
| VAV3 | Homo sapiens vav 3 guanine nucleotide exchange factor (VAV3), transcript variant 1, mRNA [NM_006113] | 1.427099 | down |
| KIAA1875 | Homo sapiens KIAA1875 (KIAA1875), non-coding RNA [NR_024207] | 1.2951833 | up |
| PDK2 | Homo sapiens pyruvate dehydrogenase kinase, isozyme 2 (PDK2), nuclear gene encoding mitochondrial protein, transcript variant 1, mRNA [NM_002611] | 1.2222253 | down |
| MKS1 | Homo sapiens Meckel syndrome, type 1 (MKS1), transcript variant 1, mRNA [NM_017777] | 1.207259 | down |
| RFX8 | Homo sapiens regulatory factor X, 8 (RFX8), mRNA [NM_001145664] | 1.6612246 | up |
|  | lincRNA:chrX:68147000-68166900 reverse strand | 5.915538 | up |
|  | lincRNA:chr21:44907647-44916227 reverse strand | 1.2658473 | down |
| KCNH2 | Homo sapiens potassium voltage-gated channel, subfamily H (eag-related), member 2 (KCNH2), transcript variant 2, mRNA [NM_172056] | 1.231455 | down |
| LRRC27 | Homo sapiens leucine rich repeat containing 27 (LRRC27), transcript variant 4, mRNA [NM_001143759] | 1.3518431 | down |
| WNT9B | Homo sapiens wingless-type MMTV integration site family, member 9B (WNT9B), mRNA [NM_003396] | 1.3293214 | down |
| MCM9 | Homo sapiens minichromosome maintenance complex component 9 (MCM9), transcript variant 2, mRNA [NM_153255] | 1.2147297 | down |
| CITED2 | Homo sapiens Cbp/p300-interacting transactivator, with Glu/Asp-rich carboxy-terminal domain, 2 (CITED2), transcript variant 1, mRNA [NM_006079] | 1.3733646 | up |
| BDH1 | Homo sapiens 3-hydroxybutyrate dehydrogenase, type 1 (BDH1), nuclear gene encoding mitochondrial protein, transcript variant 3, mRNA [NM_203314] | 1.3337286 | down |
|  |  | 1.3628012 | down |
| PRIM1 | Homo sapiens primase, DNA, polypeptide 1 (49kDa) (PRIM1), mRNA [NM_000946] | 1.3284235 | down |
| RARRES3 | Homo sapiens retinoic acid receptor responder (tazarotene induced) 3 (RARRES3), mRNA [NM_004585] | 1.789093 | up |
|  |  | 1.3207119 | down |
| P2RY2 | Homo sapiens purinergic receptor P2Y, G-protein coupled, 2 (P2RY2), transcript variant 1, mRNA [NM_176072] | 1.63183 | up |
| DCAF11 | Homo sapiens DDB1 and CUL4 associated factor 11 (DCAF11), transcript variant 1, mRNA [NM_025230] | 1.2026372 | down |
| RND3 | Homo sapiens Rho family GTPase 3 (RND3), mRNA [NM_005168] | 1.4009705 | up |
|  | lincRNA:chr4:8352025-8359075 reverse strand | 1.2162248 | up |
| TBL1X | Homo sapiens transducin (beta)-like 1X-linked (TBL1X), transcript variant 1, mRNA [NM_005647] | 1.2830127 | down |
|  | lincRNA:chr8:128807043-128820943 forward strand | 1.2321641 | down |
| CUTC | Homo sapiens cutC copper transporter homolog (E. coli) (CUTC), mRNA [NM_015960] | 1.2789567 | down |
| TMEM177 | Homo sapiens transmembrane protein 177 (TMEM177), transcript variant 2, mRNA [NM_030577] | 1.2849091 | down |
| EBF2 | Homo sapiens early B-cell factor 2 (EBF2), mRNA [NM_022659] | 5.9128637 | up |
| RPA4 | Homo sapiens replication protein A4, 30kDa (RPA4), mRNA [NM_013347] | 1.355732 | up |
| PSG1 | Homo sapiens pregnancy specific beta-1-glycoprotein 1 (PSG1), transcript variant 1, mRNA [NM_006905] | 1.5253483 | up |
| PTGR2 | Homo sapiens prostaglandin reductase 2 (PTGR2), transcript variant 1, mRNA [NM_152444] | 1.255306 | down |
|  | lincRNA:chr4:42095918-42105968 reverse strand | 1.7595857 | up |
| C15orf50 | Homo sapiens chromosome 15 open reading frame 50 (C15orf50), non-coding RNA [NR_026764] | 5.8366585 | up |
| REEP6 | Homo sapiens receptor accessory protein 6 (REEP6), mRNA [NM_138393] | 1.2471461 | down |
| IFI27 | Homo sapiens interferon, alpha-inducible protein 27 (IFI27), transcript variant 2, mRNA [NM_005532] | 5.53059 | up |
| C21orf33 | Homo sapiens chromosome 21 open reading frame 33 (C21orf33), nuclear gene encoding mitochondrial protein, transcript variant 1, mRNA [NM_004649] | 1.2206995 | down |
|  | lincRNA:chr13:31179400-31191346 reverse strand | 5.484109 | up |
| HIST1H2AI | Homo sapiens histone cluster 1, H2ai (HIST1H2AI), mRNA [NM_003509] | 1.541755 | down |
| NAPEPLD | Homo sapiens N-acyl phosphatidylethanolamine phospholipase D (NAPEPLD), transcript variant 1, mRNA [NM_001122838] | 1.2542067 | down |
| CORIN | Homo sapiens corin, serine peptidase (CORIN), mRNA [NM_006587] | 5.039831 | up |
| TBL1X | Homo sapiens transducin (beta)-like 1X-linked (TBL1X), transcript variant 1, mRNA [NM_005647] | 1.2314832 | down |
| ANKRD33B | Homo sapiens ankyrin repeat domain 33B (ANKRD33B), mRNA [NM_001164440] | 1.429199 | up |
| LOC100131242 | Homo sapiens cDNA FLJ42492 fis, clone BRACE2033720. [AK124483] | 1.3279916 | down |
| PTBP2 | Homo sapiens polypyrimidine tract binding protein 2 (PTBP2), mRNA [NM_021190] | 1.2626957 | up |
| DENND2C | DENN/MADD domain containing 2C [Source:HGNC Symbol;Acc:24748] [ENST00000369540] | 1.2187619 | up |
| NT5E | Homo sapiens 5'-nucleotidase, ecto (CD73) (NT5E), transcript variant 1, mRNA [NM_002526] | 1.7698766 | up |
| LOC253039 | Homo sapiens uncharacterized LOC253039 (LOC253039), non-coding RNA [NR_024408] | 1.2851938 | down |
|  | lincRNA:chrX:15690954-15744304 reverse strand | 1.4657989 | up |
| GBP3 | Homo sapiens guanylate binding protein 3 (GBP3), mRNA [NM_018284] | 1.4531174 | up |
| ASCC1 | Homo sapiens activating signal cointegrator 1 complex subunit 1 (ASCC1), transcript variant 3, mRNA [NM_015947] | 1.201743 | down |
|  | lincRNA:chr15:74781942-74809300 forward strand | 1.3142523 | down |
| KAT2A | Homo sapiens K(lysine) acetyltransferase 2A (KAT2A), mRNA [NM_021078] | 1.2024238 | down |
| EXPH5 | Homo sapiens exophilin 5 (EXPH5), mRNA [NM_015065] | 1.283548 | down |
| MBD5 | Homo sapiens methyl-CpG binding domain protein 5 (MBD5), mRNA [NM_018328] | 5.0059857 | up |
| IGFLR1 | Homo sapiens IGF-like family receptor 1 (IGFLR1), mRNA [NM_024660] | 1.298851 | down |
| OR10AD1 | Homo sapiens olfactory receptor, family 10, subfamily AD, member 1 (OR10AD1), mRNA [NM_001004134] | 4.983494 | up |
| KRT23 | Homo sapiens keratin 23 (histone deacetylase inducible) (KRT23), mRNA [NM_015515] | 4.609063 | up |
| IRF9 | Homo sapiens interferon regulatory factor 9 (IRF9), mRNA [NM_006084] | 1.7654378 | up |
|  | lincRNA:chr16:14398724-14425224 reverse strand | 4.6052027 | up |
|  | lincRNA:chr1:9209361-9210752 forward strand | 1.2836056 | up |
| PNMA6C | Homo sapiens paraneoplastic antigen like 6C (PNMA6C), mRNA [NM_001170944] | 1.2079619 | up |
| CENPN | Homo sapiens centromere protein N (CENPN), transcript variant 1, mRNA [NM_001100625] | 1.2731584 | down |
| FOXO3 | Homo sapiens forkhead box O3 (FOXO3), transcript variant 1, mRNA [NM_001455] | 1.2009268 | up |
| NBEAP1 | Homo sapiens neurobeachin pseudogene 1 (NBEAP1), non-coding RNA [NR_027992] | 1.3405347 | up |
| ANK3 | Homo sapiens ankyrin 3, node of Ranvier (ankyrin G) (ANK3), transcript variant 4, mRNA [NM_001204404] | 4.591236 | up |
| OMA1 | Homo sapiens OMA1 homolog, zinc metallopeptidase (S. cerevisiae) (OMA1), mRNA [NM_145243] | 1.2956542 | down |
| SNAI2 | Homo sapiens snail homolog 2 (Drosophila) (SNAI2), mRNA [NM_003068] | 1.5130798 | up |
| LBH | Homo sapiens limb bud and heart development homolog (mouse) (LBH), mRNA [NM_030915] | 1.2725669 | up |
| FAM46A | Homo sapiens family with sequence similarity 46, member A (FAM46A), mRNA [NM_017633] | 1.3435221 | down |
| COQ5 | Homo sapiens coenzyme Q5 homolog, methyltransferase (S. cerevisiae) (COQ5), nuclear gene encoding mitochondrial protein, mRNA [NM_032314] | 1.2160428 | down |
| PTPRB | Homo sapiens protein tyrosine phosphatase, receptor type, B (PTPRB), transcript variant 2, mRNA [NM_002837] | 1.3468239 | up |
| DCUN1D3 | Homo sapiens DCN1, defective in cullin neddylation 1, domain containing 3 (S. cerevisiae) (DCUN1D3), mRNA [NM_173475] | 1.2361854 | up |
| PRUNE | Homo sapiens prune homolog (Drosophila) (PRUNE), mRNA [NM_021222] | 1.2028207 | down |
| LOC257358 | Homo sapiens uncharacterized LOC257358 (LOC257358), non-coding RNA [NR_026945] | 1.5985284 | down |
| EDN1 | Homo sapiens endothelin 1 (EDN1), transcript variant 1, mRNA [NM_001955] | 1.4569212 | up |
|  | Homo sapiens cDNA FLJ30593 fis, clone BRAWH2008790. [AK055155] | 1.4351861 | down |
| DEFB124 | Homo sapiens defensin, beta 124 (DEFB124), mRNA [NM_001037500] | 1.4862448 | up |
| BAMBI | Homo sapiens BMP and activin membrane-bound inhibitor homolog (Xenopus laevis) (BAMBI), mRNA [NM_012342] | 1.3190714 | up |
| GPR143 | Homo sapiens G protein-coupled receptor 143 (GPR143), mRNA [NM_000273] | 1.4293724 | up |
| GJC1 | Homo sapiens gap junction protein, gamma 1, 45kDa (GJC1), transcript variant 1, mRNA [NM_005497] | 1.252011 | up |
|  | full-length cDNA clone CS0DL001YG15 of B cells (Ramos cell line) Cot 25-normalized of Homo sapiens (human) [CR591103] | 1.9799324 | up |
| COL9A1 | Homo sapiens collagen, type IX, alpha 1 (COL9A1), transcript variant 1, mRNA [NM_001851] | 1.2491995 | up |
| SOX30 | Homo sapiens SRY (sex determining region Y)-box 30 (SOX30), transcript variant 1, mRNA [NM_178424] | 1.3039204 | down |
| FST | Homo sapiens follistatin (FST), transcript variant FST344, mRNA [NM_013409] | 1.6585295 | up |
| ERG | Homo sapiens v-ets erythroblastosis virus E26 oncogene homolog (avian) (ERG), transcript variant 2, mRNA [NM_004449] | 1.416773 | down |
| LRRC2 | Homo sapiens leucine rich repeat containing 2 (LRRC2), mRNA [NM_024512] | 1.342527 | up |
| CDK5R1 | Homo sapiens cyclin-dependent kinase 5, regulatory subunit 1 (p35) (CDK5R1), mRNA [NM_003885] | 1.4134759 | up |
| KLF10 | Homo sapiens Kruppel-like factor 10 (KLF10), transcript variant 1, mRNA [NM_005655] | 1.4782952 | up |
| PAQR9 | Homo sapiens progestin and adipoQ receptor family member IX (PAQR9), mRNA [NM_198504] | 1.8304299 | up |
| TTLL5 | Homo sapiens tubulin tyrosine ligase-like family, member 5 (TTLL5), mRNA [NM_015072] | 1.2343887 | down |
|  | lincRNA:chr10:80511394-80516912 forward strand | 1.3004112 | up |
| RUNX1 | Homo sapiens runt-related transcription factor 1 (RUNX1), transcript variant 2, mRNA [NM_001001890] | 1.3635299 | up |
| TMCC1 | Homo sapiens transmembrane and coiled-coil domain family 1 (TMCC1), transcript variant 1, mRNA [NM_001017395] | 1.3383508 | up |
| PDZD7 | Homo sapiens PDZ domain containing 7 (PDZD7), transcript variant 1, mRNA [NM_001195263] | 1.8918885 | up |
|  | lincRNA:chr2:218574905-218583393 reverse strand | 1.6778082 | down |
| SPAG9 | Homo sapiens sperm associated antigen 9 (SPAG9), transcript variant 3, mRNA [NM_003971] | 1.2444547 | up |
| CASZ1 | Homo sapiens castor zinc finger 1 (CASZ1), transcript variant 1, mRNA [NM_001079843] | 1.5555401 | up |
| GPSM2 | Homo sapiens G-protein signaling modulator 2 (GPSM2), mRNA [NM_013296] | 1.2651097 | down |
| LOC100507316 | PREDICTED: Homo sapiens hypothetical LOC100507316, transcript variant 2 (LOC100507316), miscRNA [XR_133508] | 1.2418652 | down |
| ANGEL1 | Homo sapiens angel homolog 1 (Drosophila) (ANGEL1), mRNA [NM_015305] | 1.2776603 | down |
| C6orf192 | Homo sapiens chromosome 6 open reading frame 192 (C6orf192), mRNA [NM_052831] | 1.2418283 | down |
| ROR1 | Homo sapiens receptor tyrosine kinase-like orphan receptor 1 (ROR1), transcript variant 1, mRNA [NM_005012] | 1.2607716 | up |
| ZNF512B | Homo sapiens zinc finger protein 512B (ZNF512B), mRNA [NM_020713] | 1.2092795 | down |
| ETFA | Homo sapiens electron-transfer-flavoprotein, alpha polypeptide (ETFA), nuclear gene encoding mitochondrial protein, transcript variant 1, mRNA [NM_000126] | 1.3193426 | down |
| TENC1 | Homo sapiens tensin like C1 domain containing phosphatase (tensin 2) (TENC1), transcript variant 1, mRNA [NM_015319] | 1.3568672 | down |
| PKDREJ | Homo sapiens polycystic kidney disease (polycystin) and REJ homolog (sperm receptor for egg jelly homolog, sea urchin) (PKDREJ), mRNA [NM_006071] | 1.213759 | down |
| LOC100506190 |  | 1.2593985 | down |
| FLYWCH1 | Homo sapiens FLYWCH-type zinc finger 1 (FLYWCH1), transcript variant 1, mRNA [NM_032296] | 1.7653705 | up |
| SDC4 | Homo sapiens syndecan 4 (SDC4), mRNA [NM_002999] | 1.5699774 | up |
| RAPGEF3 | Homo sapiens Rap guanine nucleotide exchange factor (GEF) 3 (RAPGEF3), transcript variant 2, mRNA [NM_006105] | 1.3269075 | up |
| P39188 | ALU1_HUMAN (P39188) Alu subfamily J sequence contamination warning entry, partial (7%) [THC2525908] | 1.6005138 | up |
| DYNC2H1 | Homo sapiens dynein, cytoplasmic 2, heavy chain 1 (DYNC2H1), transcript variant 2, mRNA [NM_001080463] | 1.2278992 | down |
| SERPINE1 | Homo sapiens serpin peptidase inhibitor, clade E (nexin, plasminogen activator inhibitor type 1), member 1 (SERPINE1), transcript variant 1, mRNA [NM_000602] | 1.7274132 | up |
| GDF15 | Homo sapiens growth differentiation factor 15 (GDF15), mRNA [NM_004864] | 1.9862024 | up |
| GGA1 | Homo sapiens golgi-associated, gamma adaptin ear containing, ARF binding protein 1 (GGA1), transcript variant 5, mRNA [NM_001172688] | 1.273307 | down |
| C21orf104 | Homo sapiens mRNA for hypothetical protein, partial. [AJ011409] | 1.438056 | down |
| STIP1 | Homo sapiens stress-induced-phosphoprotein 1 (STIP1), mRNA [NM_006819] | 1.2425948 | down |
|  | lincRNA:chr2:130194980-130200205 reverse strand | 1.4376867 | down |
| XLOC_005798 | BROAD Institute lincRNA (XLOC_005798), lincRNA [TCONS_00012240] | 1.7258611 | up |
| NBEAL2 | neurobeachin-like 2 [Source:HGNC Symbol;Acc:31928] [ENST00000296147] | 1.5167996 | down |
| RAPGEFL1 | Homo sapiens Rap guanine nucleotide exchange factor (GEF)-like 1 (RAPGEFL1), mRNA [NM_016339] | 1.5929958 | up |
| SMOC1 | Homo sapiens SPARC related modular calcium binding 1 (SMOC1), transcript variant 1, mRNA [NM_001034852] | 1.2508188 | down |
| MXD3 | Homo sapiens MAX dimerization protein 3 (MXD3), transcript variant 1, mRNA [NM_031300] | 1.2439626 | down |
| NOG | Homo sapiens noggin (NOG), mRNA [NM_005450] | 1.2608179 | up |
| OR5F1 | Homo sapiens olfactory receptor, family 5, subfamily F, member 1 (OR5F1), mRNA [NM_003697] | 1.3672866 | up |
| TTC9C | tetratricopeptide repeat domain 9C [Source:HGNC Symbol;Acc:28432] [ENST00000294161] | 1.2295051 | down |
|  |  | 4.5811377 | up |
| PODXL | Homo sapiens podocalyxin-like (PODXL), transcript variant 1, mRNA [NM_001018111] | 1.2508376 | up |
|  | lincRNA:chr3:155663806-155671056 forward strand | 1.5901955 | up |
| OSBPL5 | Homo sapiens oxysterol binding protein-like 5 (OSBPL5), transcript variant 1, mRNA [NM_020896] | 1.3014226 | down |
|  |  | 1.4133586 | down |
| BCL2L1 | Homo sapiens BCL2-like 1 (BCL2L1), nuclear gene encoding mitochondrial protein, transcript variant 1, mRNA [NM_138578] | 1.2023691 | up |
| KLK2 | Homo sapiens kallikrein-related peptidase 2 (KLK2), transcript variant 2, mRNA [NM_001002231] | 1.5838519 | up |
| GPATCH1 | Homo sapiens G patch domain containing 1 (GPATCH1), mRNA [NM_018025] | 1.2484603 | down |
| LSM2 | Homo sapiens LSM2 homolog, U6 small nuclear RNA associated (S. cerevisiae) (LSM2), mRNA [NM_021177] | 1.2230575 | down |
| APOE | Homo sapiens apolipoprotein E (APOE), mRNA [NM_000041] | 1.3274893 | up |
| GJA3 | Homo sapiens gap junction protein, alpha 3, 46kDa (GJA3), mRNA [NM_021954] | 4.421641 | up |
| C3orf17 | Homo sapiens chromosome 3 open reading frame 17 (C3orf17), transcript variant 1, mRNA [NM_015412] | 1.2820601 | down |
| IRS2 | Homo sapiens insulin receptor substrate 2 (IRS2), mRNA [NM_003749] | 1.2504034 | up |
| ABLIM2 | Homo sapiens actin binding LIM protein family, member 2 (ABLIM2), transcript variant 6, mRNA [NM_032432] | 1.264699 | up |
| UFSP1 | Homo sapiens UFM1-specific peptidase 1 (non-functional) (UFSP1), mRNA [NM_001015072] | 1.2133061 | down |
| FRMD5 | Homo sapiens FERM domain containing 5 (FRMD5), transcript variant 2, mRNA [NM_032892] | 1.3065083 | up |
| NCF2 | Homo sapiens neutrophil cytosolic factor 2 (NCF2), transcript variant 1, mRNA [NM_000433] | 1.7276777 | up |
| ZNF148 | Homo sapiens zinc finger protein 148 (ZNF148), mRNA [NM_021964] | 1.2206329 | down |
| C6orf163 | Homo sapiens chromosome 6 open reading frame 163 (C6orf163), mRNA [NM_001010868] | 1.5426602 | down |
| PARD6A | Homo sapiens par-6 partitioning defective 6 homolog alpha (C. elegans) (PARD6A), transcript variant 1, mRNA [NM_016948] | 1.2931665 | down |
| NDC80 | Homo sapiens NDC80 homolog, kinetochore complex component (S. cerevisiae) (NDC80), mRNA [NM_006101] | 1.2162914 | down |
| HIST1H2AK | Homo sapiens histone cluster 1, H2ak (HIST1H2AK), mRNA [NM_003510] | 1.2271988 | down |
| MB21D2 | Homo sapiens Mab-21 domain containing 2 (MB21D2), mRNA [NM_178496] | 1.2914784 | up |
| MYBPHL | Homo sapiens myosin binding protein H-like (MYBPHL), mRNA [NM_001010985] | 4.374709 | up |
|  | lincRNA:chr4:47840518-47846712 reverse strand | 1.2357935 | down |
| ARL9 | Homo sapiens ADP-ribosylation factor-like 9 (ARL9), mRNA [NM_206919] | 4.3043528 | up |
| LRPPRC | Homo sapiens leucine-rich PPR-motif containing (LRPPRC), mRNA [NM_133259] | 1.21414 | down |
| C14orf159 | Homo sapiens chromosome 14 open reading frame 159 (C14orf159), transcript variant 3, mRNA [NM_024952] | 1.2800113 | down |
| PRPF18 | PRP18 pre-mRNA processing factor 18 homolog (S. cerevisiae) [Source:HGNC Symbol;Acc:17351] [ENST00000320054] | 1.2286332 | up |
| UGT2B15 | Homo sapiens UDP glucuronosyltransferase 2 family, polypeptide B15 (UGT2B15), mRNA [NM_001076] | 1.4139754 | down |
| COA5 | Homo sapiens cytochrome C oxidase assembly factor 5 (COA5), mRNA [NM_001008215] | 1.2488067 | down |
| BTBD3 | Homo sapiens BTB (POZ) domain containing 3 (BTBD3), transcript variant 1, mRNA [NM_014962] | 1.27654 | down |
| GTPBP6 | Homo sapiens GTP binding protein 6 (putative) (GTPBP6), mRNA [NM_012227] | 1.2157271 | down |
| B4GALNT4 | Homo sapiens beta-1,4-N-acetyl-galactosaminyl transferase 4 (B4GALNT4), mRNA [NM_178537] | 1.3301737 | down |
| KIF15 | Homo sapiens kinesin family member 15 (KIF15), mRNA [NM_020242] | 1.255964 | down |
| FBL | Homo sapiens fibrillarin (FBL), mRNA [NM_001436] | 1.2072994 | down |
| CROCC | Homo sapiens ciliary rootlet coiled-coil, rootletin (CROCC), mRNA [NM_014675] | 1.2137567 | down |
| DBX1 | Homo sapiens developing brain homeobox 1 (DBX1), mRNA [NM_001029865] | 1.4843273 | down |
| AIMP2 | Homo sapiens aminoacyl tRNA synthetase complex-interacting multifunctional protein 2 (AIMP2), mRNA [NM_006303] | 1.2135898 | down |
| PARP3 | Homo sapiens poly (ADP-ribose) polymerase family, member 3 (PARP3), transcript variant 2, mRNA [NM_005485] | 1.2116556 | down |
|  | lincRNA:chr20:10607175-10613750 forward strand | 1.2335696 | down |
| MAPK8 | Homo sapiens mitogen-activated protein kinase 8 (MAPK8), transcript variant JNK1-b2, mRNA [NM_139047] | 1.7772 | up |
| LILRB3 | Homo sapiens leukocyte immunoglobulin-like receptor, subfamily B (with TM and ITIM domains), member 3 (LILRB3), transcript variant 2, mRNA [NM_006864] | 1.6643647 | up |
| IMPA2 | Homo sapiens inositol(myo)-1(or 4)-monophosphatase 2 (IMPA2), mRNA [NM_014214] | 1.3754127 | down |
| PHTF1 | Homo sapiens putative homeodomain transcription factor 1 (PHTF1), mRNA [NM_006608] | 1.2029641 | down |
| IFT140 | Homo sapiens intraflagellar transport 140 homolog (Chlamydomonas) (IFT140), mRNA [NM_014714] | 1.353376 | down |
| RHOBTB2 | Homo sapiens Rho-related BTB domain containing 2 (RHOBTB2), transcript variant 3, mRNA [NM_015178] | 1.2135482 | down |
| C19orf59 | Homo sapiens chromosome 19 open reading frame 59 (C19orf59), mRNA [NM_174918] | 1.5923606 | down |
| FAM46B | Homo sapiens family with sequence similarity 46, member B (FAM46B), mRNA [NM_052943] | 1.2128127 | up |
| LMO4 | Homo sapiens LIM domain only 4 (LMO4), mRNA [NM_006769] | 1.2552209 | down |
| SSBP2 | Homo sapiens single-stranded DNA binding protein 2 (SSBP2), mRNA [NM_012446] | 1.2420666 | down |
| PYCRL | Homo sapiens pyrroline-5-carboxylate reductase-like (PYCRL), mRNA [NM_023078] | 1.2071034 | down |
| LARP6 | Homo sapiens La ribonucleoprotein domain family, member 6 (LARP6), transcript variant 1, mRNA [NM_018357] | 1.3160002 | up |
| B2M | Homo sapiens beta-2-microglobulin (B2M), mRNA [NM_004048] | 1.2816403 | up |
| HEPHL1 | Homo sapiens hephaestin-like 1 (HEPHL1), mRNA [NM_001098672] | 4.205481 | up |
| DNHD1 | Homo sapiens dynein heavy chain domain 1 (DNHD1), transcript variant 1, mRNA [NM_144666] | 1.3972046 | up |
| XLOC_l2_010558 | BROAD Institute lincRNA (XLOC_l2_010558), lincRNA [TCONS_l2_00020391] | 1.44999 | down |
|  | lincRNA:chr21:40541155-40546455 forward strand | 1.3021162 | down |
| LOC100127946 | PREDICTED: Homo sapiens hypothetical protein LOC100127946 (LOC100127946), mRNA [XM_001717040] | 1.4035304 | down |
| HERC6 | Homo sapiens hect domain and RLD 6 (HERC6), transcript variant 1, mRNA [NM_017912] | 4.1981096 | up |
| HIST1H2BF | Homo sapiens histone cluster 1, H2bf (HIST1H2BF), mRNA [NM_003522] | 1.3076099 | down |
| LOC202025 | Homo sapiens mRNA; cDNA DKFZp667E0512 (from clone DKFZp667E0512). [AL713660] | 1.203991 | up |
| C2orf27A | Homo sapiens chromosome 2 open reading frame 27A (C2orf27A), mRNA [NM_013310] | 1.3022742 | down |
| DEPDC5 | Homo sapiens DEP domain containing 5 (DEPDC5), transcript variant 4, mRNA [NM_001242896] | 1.2528949 | down |
|  | lincRNA:chr4:54788293-54804443 reverse strand | 1.574592 | down |
| DOK7 | Homo sapiens docking protein 7 (DOK7), transcript variant 1, mRNA [NM_173660] | 1.4515353 | up |
| FAM105A | Homo sapiens family with sequence similarity 105, member A (FAM105A), mRNA [NM_019018] | 1.3188547 | down |
| TSIX | Homo sapiens TSIX transcript, XIST antisense RNA (non-protein coding) (TSIX), antisense RNA [NR_003255] | 1.3638555 | down |
| LYST | Homo sapiens lysosomal trafficking regulator (LYST), mRNA [NM_000081] | 1.2667392 | up |
| TRANK1 | Homo sapiens tetratricopeptide repeat and ankyrin repeat containing 1 (TRANK1), mRNA [NM_014831] | 1.5256264 | up |
| ANAPC1 | Homo sapiens anaphase promoting complex subunit 1 (ANAPC1), mRNA [NM_022662] | 1.2503537 | down |
|  |  | 1.3520026 | down |
| LOC100505702 | Homo sapiens uncharacterized LOC100505702 (LOC100505702), transcript variant 6, non-coding RNA [NR_038308] | 1.4218988 | up |
| STC2 | Homo sapiens stanniocalcin 2 (STC2), mRNA [NM_003714] | 1.3768082 | up |
|  | lincRNA:chrX:102074319-102166119 forward strand | 1.379686 | up |
| HOXB2 | Homo sapiens homeobox B2 (HOXB2), mRNA [NM_002145] | 1.2254238 | up |
| ISG20 | Homo sapiens interferon stimulated exonuclease gene 20kDa (ISG20), mRNA [NM_002201] | 4.081272 | up |
| LRIT1 | Homo sapiens leucine-rich repeat, immunoglobulin-like and transmembrane domains 1 (LRIT1), mRNA [NM_015613] | 3.9907932 | up |
|  | lincRNA:chr3:156455706-156471081 reverse strand | 1.3062949 | down |
| MKL2 | Homo sapiens MKL/myocardin-like 2 (MKL2), mRNA [NM_014048] | 1.2559814 | down |
| MAP1A | Homo sapiens microtubule-associated protein 1A (MAP1A), mRNA [NM_002373] | 1.3763278 | down |
| DLX1 | Homo sapiens distal-less homeobox 1 (DLX1), transcript variant 1, mRNA [NM_178120] | 1.2154667 | down |
| PGF | Homo sapiens placental growth factor (PGF), transcript variant 1, mRNA [NM_002632] | 1.4699726 | up |
| ARPC4-TTLL3 | Homo sapiens ARPC4-TTLL3 readthrough (ARPC4-TTLL3), mRNA [NM_001198793] | 1.2357851 | up |
| TBX2 | Homo sapiens T-box 2 (TBX2), mRNA [NM_005994] | 1.5831118 | up |
| ABL2 | Homo sapiens v-abl Abelson murine leukemia viral oncogene homolog 2 (ABL2), transcript variant b, mRNA [NM_007314] | 1.3484657 | up |
| CBY1 | Homo sapiens chibby homolog 1 (Drosophila) (CBY1), transcript variant 2, mRNA [NM_001002880] | 1.273364 | down |
| MRGPRF | Homo sapiens MAS-related GPR, member F (MRGPRF), transcript variant 2, mRNA [NM_145015] | 1.3191546 | down |
| CBX5 | Homo sapiens chromobox homolog 5 (CBX5), transcript variant 1, mRNA [NM_001127322] | 1.2191503 | down |
| ANXA11 | annexin A11 [Source:HGNC Symbol;Acc:535] [ENST00000372234] | 1.3608043 | down |
| FBXO10 | Homo sapiens F-box protein 10 (FBXO10), mRNA [NM_012166] | 1.294016 | up |
|  | lincRNA:chr19:56795502-56798051 reverse strand | 1.399628 | up |
| ZC3HAV1 | Homo sapiens zinc finger CCCH-type, antiviral 1 (ZC3HAV1), transcript variant 2, mRNA [NM_024625] | 1.6464332 | up |
| MKI67 | Homo sapiens antigen identified by monoclonal antibody Ki-67 (MKI67), transcript variant 1, mRNA [NM_002417] | 1.2825783 | down |
| L2HGDH | Homo sapiens L-2-hydroxyglutarate dehydrogenase (L2HGDH), nuclear gene encoding mitochondrial protein, mRNA [NM_024884] | 1.216369 | down |
| NEFH | Homo sapiens neurofilament, heavy polypeptide (NEFH), mRNA [NM_021076] | 1.2014933 | down |
| XLOC_005327 | NM_105511 ATP binding {Arabidopsis thaliana} (exp=-1; wgp=0; cg=0), partial (4%) [THC2716204] | 1.3664447 | up |
| ABCC6 | Homo sapiens ATP-binding cassette, sub-family C (CFTR/MRP), member 6 (ABCC6), transcript variant 2, mRNA [NM_001079528] | 1.3129479 | up |
| JUN | Homo sapiens jun proto-oncogene (JUN), mRNA [NM_002228] | 1.6743962 | up |
| LOC100131067 | Homo sapiens uncharacterized LOC100131067 (LOC100131067), transcript variant 1, non-coding RNA [NR_034121] | 1.4479845 | down |
|  | lincRNA:chr5:77285769-77295769 forward strand | 1.4442441 | up |
| XLOC_002286 | BROAD Institute lincRNA (XLOC_002286), lincRNA [TCONS_00004414] | 1.2958257 | down |
| ZNF645 | Homo sapiens zinc finger protein 645 (ZNF645), mRNA [NM_152577] | 3.9077044 | up |
|  | lincRNA:chrX:98168819-98180169 reverse strand | 1.7240725 | down |
| ARID3B | Homo sapiens AT rich interactive domain 3B (BRIGHT-like) (ARID3B), mRNA [NM_006465] | 1.2806368 | up |
| NEXN | Homo sapiens nexilin (F actin binding protein) (NEXN), transcript variant 1, mRNA [NM_144573] | 1.295029 | up |
| IL7R | Homo sapiens interleukin 7 receptor (IL7R), mRNA [NM_002185] | 1.5346459 | up |
| TMBIM1 | Homo sapiens transmembrane BAX inhibitor motif containing 1 (TMBIM1), mRNA [NM_022152] | 1.3218538 | up |
| FLJ32255 | PREDICTED: Homo sapiens hypothetical LOC643977 (FLJ32255), miscRNA [XR_108575] | 1.4693143 | up |
| EMP1 | Homo sapiens epithelial membrane protein 1 (EMP1), mRNA [NM_001423] | 1.3953197 | up |
|  | lincRNA:chr6:155240958-155286533 forward strand | 1.6620679 | up |
| LOC100128670 | Homo sapiens cDNA FLJ44998 fis, clone BRAWH3010833. [AK126945] | 1.332645 | down |
| SLC19A3 | Homo sapiens solute carrier family 19, member 3 (SLC19A3), mRNA [NM_025243] | 1.4092767 | down |
| XLOC_l2_003666 | BROAD Institute lincRNA (XLOC_l2_003666), lincRNA [TCONS_l2_00007378] | 1.3821989 | down |
| HTR1D | 5-hydroxytryptamine (serotonin) receptor 1D [Source:HGNC Symbol;Acc:5289] [ENST00000314113] | 1.3201553 | up |
| HIST1H3B | Homo sapiens histone cluster 1, H3b (HIST1H3B), mRNA [NM_003537] | 1.3223685 | down |
| HSPA8 | heat shock 70kDa protein 8 [Source:HGNC Symbol;Acc:5241] [ENST00000527983] | 1.3165078 | down |
| ONECUT2 | Homo sapiens one cut homeobox 2 (ONECUT2), mRNA [NM_004852] | 1.2733794 | down |
| LOC645249 | Homo sapiens uncharacterized LOC645249 (LOC645249), non-coding RNA [NR_038835] | 1.3215066 | down |
| CCDC33 | Homo sapiens coiled-coil domain containing 33 (CCDC33), transcript variant 1, mRNA [NM_025055] | 1.7421585 | up |
| GADD45A | Homo sapiens growth arrest and DNA-damage-inducible, alpha (GADD45A), transcript variant 1, mRNA [NM_001924] | 1.7873728 | up |
| GPRC5B | Homo sapiens G protein-coupled receptor, family C, group 5, member B (GPRC5B), mRNA [NM_016235] | 1.5471522 | up |
| MYOM3 | Homo sapiens myomesin family, member 3 (MYOM3), mRNA [NM_152372] | 1.2877454 | up |
| PARN | Homo sapiens poly(A)-specific ribonuclease (PARN), transcript variant 1, mRNA [NM_002582] | 1.2846341 | down |
| PRIC285 | Homo sapiens peroxisomal proliferator-activated receptor A interacting complex 285 (PRIC285), transcript variant 1, mRNA [NM_001037335] | 1.6414539 | up |
| C1orf54 | Homo sapiens chromosome 1 open reading frame 54 (C1orf54), mRNA [NM_024579] | 1.4667262 | up |
| MOXD2P | Homo sapiens monooxygenase, DBH-like 2, pseudogene (MOXD2P), non-coding RNA [NR_024346] | 1.265286 | down |
| TYSND1 | Homo sapiens trypsin domain containing 1 (TYSND1), transcript variant 1, mRNA [NM_173555] | 1.2195283 | down |
| LOC388906 | Homo sapiens opioid growth factor receptor pseudogene (LOC388906), non-coding RNA [NR_036498] | 1.2129107 | down |
| PLA2G6 | Homo sapiens phospholipase A2, group VI (cytosolic, calcium-independent) (PLA2G6), transcript variant 1, mRNA [NM_003560] | 1.8226699 | up |
| SFXN2 | Homo sapiens sideroflexin 2 (SFXN2), mRNA [NM_178858] | 1.2262222 | down |
|  | Homo sapiens Williams-Beuren syndrome chromosome region 23, mRNA (cDNA clone MGC:161587 IMAGE:8992025), complete cds. [BC126309] | 1.2570474 | down |
| NRG1 | Homo sapiens neuregulin 1 (NRG1), transcript variant HRG-gamma, mRNA [NM_004495] | 1.5016973 | up |
| LOC100506190 |  | 1.2567527 | down |
| SULF1 | Homo sapiens sulfatase 1 (SULF1), transcript variant 3, mRNA [NM_015170] | 1.5800663 | up |
| ANTXR2 | Homo sapiens anthrax toxin receptor 2 (ANTXR2), transcript variant 1, mRNA [NM_058172] | 1.7499909 | up |
| ABL2 | Homo sapiens v-abl Abelson murine leukemia viral oncogene homolog 2 (ABL2), transcript variant b, mRNA [NM_007314] | 1.338702 | up |
| SHROOM2 | Homo sapiens shroom family member 2 (SHROOM2), mRNA [NM_001649] | 1.5840685 | up |
| LTK | Homo sapiens leukocyte receptor tyrosine kinase (LTK), transcript variant 1, mRNA [NM_002344] | 1.4322505 | up |
|  | lincRNA:chr15:95807796-95868971 forward strand | 3.8768933 | up |
| H1FX-AS1 | Homo sapiens H1FX antisense RNA 1 (non-protein coding) (H1FX-AS1), non-coding RNA [NR_026991] | 1.2702398 | down |
| NKPD1 | Homo sapiens NTPase, KAP family P-loop domain containing 1 (NKPD1), mRNA [NM_198478] | 1.520669 | up |
| B3GNT5 | Homo sapiens UDP-GlcNAc:betaGal beta-1,3-N-acetylglucosaminyltransferase 5 (B3GNT5), mRNA [NM_032047] | 1.2313402 | up |
| WDR74 | WD repeat domain 74 [Source:HGNC Symbol;Acc:25529] [ENST00000538098] | 1.297238 | down |
| HIST1H4A | Homo sapiens histone cluster 1, H4a (HIST1H4A), mRNA [NM_003538] | 1.2104318 | down |
| UBXN7 | Homo sapiens UBX domain protein 7 (UBXN7), mRNA [NM_015562] | 1.5462825 | down |
| CASQ1 | Homo sapiens calsequestrin 1 (fast-twitch, skeletal muscle) (CASQ1), nuclear gene encoding mitochondrial protein, mRNA [NM_001231] | 1.2194936 | up |
| SLC4A9 | Homo sapiens solute carrier family 4, sodium bicarbonate cotransporter, member 9 (SLC4A9), mRNA [NM_031467] | 1.3460511 | down |
| NUDT16L1 | Homo sapiens nudix (nucleoside diphosphate linked moiety X)-type motif 16-like 1 (NUDT16L1), transcript variant 1, mRNA [NM_032349] | 1.2211797 | down |
| MUTYH | Homo sapiens mutY homolog (E. coli) (MUTYH), transcript variant alpha1, mRNA [NM_012222] | 1.2687205 | down |
| PRAMEF5 | Homo sapiens PRAME family member 5 (PRAMEF5), mRNA [NM_001013407] | 1.7753475 | down |
| HAUS7 | Homo sapiens HAUS augmin-like complex, subunit 7 (HAUS7), mRNA [NM_017518] | 1.2481662 | down |
| PPIL4 | Homo sapiens peptidylprolyl isomerase (cyclophilin)-like 4 (PPIL4), mRNA [NM_139126] | 1.4466336 | up |
| ADORA3 | Homo sapiens adenosine A3 receptor (ADORA3), transcript variant 1, mRNA [NM_020683] | 1.357341 | up |
|  | lincRNA:chr6:29709195-29716778 reverse strand | 1.3934112 | up |
| ALDH3A1 | Homo sapiens aldehyde dehydrogenase 3 family, member A1 (ALDH3A1), transcript variant 1, mRNA [NM_001135168] | 1.371915 | down |
| ABHD2 | Homo sapiens abhydrolase domain containing 2 (ABHD2), transcript variant 1, mRNA [NM_007011] | 1.2249346 | up |
| VAPA | Homo sapiens VAMP (vesicle-associated membrane protein)-associated protein A, 33kDa (VAPA), transcript variant 1, mRNA [NM_003574] | 1.3126364 | down |
|  |  | 1.3189075 | down |
|  |  | 1.3891546 | up |
| MIR137HG | Homo sapiens cDNA FLJ35409 fis, clone SKNSH2009435. [AK092728] | 1.3098257 | up |
| HBEGF | Homo sapiens heparin-binding EGF-like growth factor (HBEGF), mRNA [NM_001945] | 1.6641798 | up |
|  | lincRNA:chr13:50664690-50666442 reverse strand | 1.4200561 | down |
|  | lincRNA:chr5:38812198-38814135 reverse strand | 1.2134075 | up |
| DKK1 | Homo sapiens dickkopf homolog 1 (Xenopus laevis) (DKK1), mRNA [NM_012242] | 1.8052841 | up |
| IFI44 | Homo sapiens interferon-induced protein 44 (IFI44), mRNA [NM_006417] | 3.789871 | up |
| RASAL2 | Homo sapiens RAS protein activator like 2 (RASAL2), transcript variant 2, mRNA [NM_170692] | 1.3015949 | up |
|  | lincRNA:chr1:41956288-41971088 forward strand | 3.7769814 | up |
| IER3 | Homo sapiens immediate early response 3 (IER3), mRNA [NM_003897] | 1.358217 | up |
| TRIM46 | Homo sapiens tripartite motif containing 46 (TRIM46), mRNA [NM_025058] | 1.2165167 | down |
| AK2 | Homo sapiens adenylate kinase 2 (AK2), nuclear gene encoding mitochondrial protein, transcript variant 2, mRNA [NM_013411] | 1.2061425 | down |
|  | lincRNA:chr12:69800933-69810508 reverse strand | 1.3050649 | down |
| DKFZp761E198 | Homo sapiens uncharacterized protein DKFZp761E198 (DKFZp761E198), mRNA [NM_138368] | 1.243756 | up |
| DLK2 | Homo sapiens delta-like 2 homolog (Drosophila) (DLK2), transcript variant 2, mRNA [NM_206539] | 1.3278288 | up |
| FAM83E | Homo sapiens family with sequence similarity 83, member E (FAM83E), mRNA [NM_017708] | 1.3394314 | up |
| FLOT2 | Homo sapiens flotillin 2 (FLOT2), mRNA [NM_004475] | 1.2422673 | down |
| NUP210 | Homo sapiens nucleoporin 210kDa (NUP210), mRNA [NM_024923] | 1.2240351 | down |
|  | lincRNA:chr17:42100749-42112035 reverse strand | 1.3557835 | up |
| EGR1 | Homo sapiens early growth response 1 (EGR1), mRNA [NM_001964] | 3.6857336 | up |
|  | lincRNA:chr14:61762697-61781247 forward strand | 1.7638034 | up |
|  | lincRNA:chr12:97866326-97868402 forward strand | 1.3541322 | up |
| OLR1 | Homo sapiens oxidized low density lipoprotein (lectin-like) receptor 1 (OLR1), transcript variant 1, mRNA [NM_002543] | 1.9968678 | up |
|  | lincRNA:chr6:3906210-3906346 forward strand | 1.2057523 | down |
|  | lincRNA:chr1:59049237-59065662 reverse strand | 1.3372346 | down |
| PBX1 | Homo sapiens pre-B-cell leukemia homeobox 1 (PBX1), transcript variant 1, mRNA [NM_002585] | 1.2228616 | down |
|  | GB | 3.665779 | up |
|  | Homo sapiens mRNA for FLJ00217 protein. [AK074144] | 1.6207718 | up |
|  | DB026495 TESTI2 Homo sapiens cDNA clone TESTI2008712 5', mRNA sequence [DB026495] | 1.8424631 | up |
| BEX5 | Homo sapiens brain expressed, X-linked 5 (BEX5), transcript variant 1, mRNA [NM_001012978] | 3.5879073 | up |
| CCDC77 | Homo sapiens coiled-coil domain containing 77 (CCDC77), transcript variant 1, mRNA [NM_032358] | 1.2281188 | down |
| CD274 | Homo sapiens CD274 molecule (CD274), mRNA [NM_014143] | 1.5377506 | up |
| LAT2 | Homo sapiens linker for activation of T cells family, member 2 (LAT2), transcript variant 1, mRNA [NM_032464] | 1.9900771 | up |
| KRTAP5-4 | Homo sapiens keratin associated protein 5-4 (KRTAP5-4), mRNA [NM_001012709] | 1.4172199 | down |
| LOC646626 | Homo sapiens uncharacterized LOC646626 (LOC646626), non-coding RNA [NR_045484] | 1.27419 | up |
| GRIN2A | Homo sapiens cDNA, FLJ98903. [AK308862] | 1.318156 | down |
| GTPBP6 | Homo sapiens GTP binding protein 6 (putative) (GTPBP6), mRNA [NM_012227] | 1.3278967 | down |
| VEGFC | Homo sapiens vascular endothelial growth factor C (VEGFC), mRNA [NM_005429] | 1.3337926 | up |
| XLOC_006725 | BROAD Institute lincRNA (XLOC_006725), lincRNA [TCONS_00014630] | 1.4963117 | up |
|  |  | 1.3384155 | up |
| SUMF1 | Homo sapiens sulfatase modifying factor 1 (SUMF1), transcript variant 1, mRNA [NM_182760] | 1.2130576 | down |
| INPP5D | Homo sapiens inositol polyphosphate-5-phosphatase, 145kDa (INPP5D), transcript variant 1, mRNA [NM_001017915] | 1.722999 | up |
| KAAG1 | Homo sapiens kidney associated antigen 1 (KAAG1), mRNA [NM_181337] | 3.5834892 | up |
| ANKMY1 | Homo sapiens ankyrin repeat and MYND domain containing 1 (ANKMY1), transcript variant 1, mRNA [NM_016552] | 1.2695303 | down |
| RHOF | Homo sapiens ras homolog gene family, member F (in filopodia) (RHOF), mRNA [NM_019034] | 1.3538158 | up |
|  | lincRNA:chr6:14649596-14677521 reverse strand | 1.2028521 | up |
| CDKN1A | Homo sapiens cyclin-dependent kinase inhibitor 1A (p21, Cip1) (CDKN1A), transcript variant 2, mRNA [NM_078467] | 1.4839529 | up |
| SLC16A10 | Homo sapiens solute carrier family 16, member 10 (aromatic amino acid transporter) (SLC16A10), mRNA [NM_018593] | 1.6122017 | down |
| PLEKHM1 | Homo sapiens pleckstrin homology domain containing, family M (with RUN domain) member 1 (PLEKHM1), transcript variant 1, mRNA [NM_014798] | 1.2290063 | up |
| LCN1 | Homo sapiens lipocalin 1 (tear prealbumin) (LCN1), mRNA [NM_002297] | 3.5834591 | up |
| NUAK1 | Homo sapiens NUAK family, SNF1-like kinase, 1 (NUAK1), mRNA [NM_014840] | 1.5447793 | up |
| HLX | Homo sapiens H2.0-like homeobox (HLX), mRNA [NM_021958] | 1.2131935 | up |
| LIMK2 | Homo sapiens LIM domain kinase 2 (LIMK2), transcript variant 1, mRNA [NM_001031801] | 1.6268892 | up |
| FLJ35390 | Homo sapiens uncharacterized LOC255031 (FLJ35390), transcript variant 2, non-coding RNA [NR_015401] | 3.5002742 | up |
|  | lincRNA:chr12:57808558-57820783 reverse strand | 1.3060261 | down |
| P39188 | ALU1_HUMAN (P39188) Alu subfamily J sequence contamination warning entry, partial (6%) [THC2579385] | 1.4100044 | down |
| ACVR1 | Homo sapiens activin A receptor, type I (ACVR1), transcript variant 1, mRNA [NM_001105] | 1.2003596 | up |
|  |  | 1.2197195 | up |
|  |  | 1.4043546 | down |
| KCNRG | Homo sapiens potassium channel regulator (KCNRG), transcript variant 2, mRNA [NM_199464] | 1.5130616 | down |
| BTN2A1 | Homo sapiens butyrophilin, subfamily 2, member A1 (BTN2A1), transcript variant 4, mRNA [NM_001197234] | 1.2561632 | up |
|  | lincRNA:chr7:128523814-128529589 forward strand | 1.2093127 | up |
| PTGER4 | Homo sapiens prostaglandin E receptor 4 (subtype EP4) (PTGER4), mRNA [NM_000958] | 1.4123204 | up |
| DKFZp451A211 | PREDICTED: Homo sapiens DKFZp451A211 protein (DKFZp451A211), mRNA [XM_003403663] | 1.3789768 | up |
| LOC100126447 | PREDICTED: Homo sapiens hypothetical LOC100126447 (LOC100126447), miscRNA [XR_109781] | 1.2631463 | up |
| DDX60 | Homo sapiens DEAD (Asp-Glu-Ala-Asp) box polypeptide 60 (DDX60), mRNA [NM_017631] | 3.4243658 | up |
|  | lincRNA:chr21:29918229-30215104 reverse strand | 1.9142 | up |
| CA6 | carbonic anhydrase VI [Source:HGNC Symbol;Acc:1380] [ENST00000319474] | 1.848261 | up |
| ITPR3 | Homo sapiens inositol 1,4,5-trisphosphate receptor, type 3 (ITPR3), mRNA [NM_002224] | 1.2380327 | up |
| NAALADL2 | N-acetylated alpha-linked acidic dipeptidase-like 2 [Source:HGNC Symbol;Acc:23219] [ENST00000495900] | 1.2308588 | up |
| AJUBA | Homo sapiens jub, ajuba homolog (Xenopus laevis) (JUB), transcript variant 1, mRNA [NM_032876] | 1.264108 | up |
| NRP1 | neuropilin 1 [Source:HGNC Symbol;Acc:8004] [ENST00000374818] | 1.229449 | up |
| LOC728705 | Homo sapiens cDNA FLJ31150 fis, clone IMR322001534. [AK055712] | 1.3331832 | down |
| WHSC1L1 | Homo sapiens Wolf-Hirschhorn syndrome candidate 1-like 1 (WHSC1L1), transcript variant long, mRNA [NM_023034] | 1.2200314 | up |
|  | lincRNA:chr20:25213650-25221750 forward strand | 1.2892379 | up |
| ISG15 | Homo sapiens ISG15 ubiquitin-like modifier (ISG15), mRNA [NM_005101] | 3.2201073 | up |
| LOC391767 | PREDICTED: Homo sapiens putative TAF11-like protein ENSP00000332601-like (LOC391767), mRNA [XM_001715028] | 1.2804279 | up |
| EPB41L4A | Homo sapiens erythrocyte membrane protein band 4.1 like 4A (EPB41L4A), mRNA [NM_022140] | 1.3839643 | down |
|  |  | 1.3094381 | up |
| DCLRE1C | Homo sapiens DNA cross-link repair 1C (DCLRE1C), transcript variant c, mRNA [NM_001033858] | 1.3526318 | up |
| TUSC5 | Homo sapiens tumor suppressor candidate 5 (TUSC5), mRNA [NM_172367] | 1.3379608 | down |
| LSP1 | Homo sapiens lymphocyte-specific protein 1 (LSP1), transcript variant 5, mRNA [NM_001242932] | 1.2116969 | up |
| N4BP2 | Homo sapiens NEDD4 binding protein 2 (N4BP2), mRNA [NM_018177] | 1.2233418 | up |
|  | lincRNA:chr6:27663246-27683586 forward strand | 1.254658 | down |
| POLD2 | Homo sapiens polymerase (DNA directed), delta 2, regulatory subunit 50kDa (POLD2), transcript variant 2, mRNA [NM_006230] | 1.2803117 | down |
| CALB1 | Homo sapiens calbindin 1, 28kDa (CALB1), mRNA [NM_004929] | 3.1544352 | up |
| PRRX2 | Homo sapiens paired related homeobox 2 (PRRX2), mRNA [NM_016307] | 1.4536844 | up |
| TRIML2 | Homo sapiens tripartite motif family-like 2 (TRIML2), mRNA [NM_173553] | 1.9720995 | up |
| FCAR | Homo sapiens Fc fragment of IgA, receptor for (FCAR), transcript variant 1, mRNA [NM_002000] | 1.288608 | up |
| GSTA2 | Homo sapiens glutathione S-transferase alpha 2 (GSTA2), mRNA [NM_000846] | 1.2078196 | up |
| RGS20 | Homo sapiens regulator of G-protein signaling 20 (RGS20), transcript variant 1, mRNA [NM_170587] | 1.373983 | up |
|  | lincRNA:chr5:144813732-145123982 reverse strand | 1.2110108 | up |
| FLJ26850 | Homo sapiens FLJ26850 protein (FLJ26850), non-coding RNA [NR_027257] | 3.1345048 | up |
| CD86 | Homo sapiens CD86 molecule (CD86), transcript variant 2, mRNA [NM_006889] | 1.2670447 | up |
|  | lincRNA:chr2:233444129-233451012 forward strand | 1.2383642 | up |
| RIT1 | Homo sapiens Ras-like without CAAX 1 (RIT1), mRNA [NM_006912] | 1.4457033 | up |
|  | lincRNA:chr14:71927902-71939072 forward strand | 1.3615971 | down |
|  | zinc finger, DHHC-type containing 11B [Source:HGNC Symbol;Acc:32962] [ENST00000522356] | 1.341592 | up |
|  | lincRNA:chr20:57408055-57413680 reverse strand | 1.4790727 | up |
| C9orf86 | Homo sapiens chromosome 9 open reading frame 86 (C9orf86), transcript variant 4, mRNA [NM_001173989] | 1.2558689 | down |
| C20orf27 | Homo sapiens chromosome 20 open reading frame 27 (C20orf27), mRNA [NM_001039140] | 1.2318649 | down |
| FAM132A | Homo sapiens family with sequence similarity 132, member A (FAM132A), mRNA [NM_001014980] | 1.2953782 | up |
| GSTA4 | Homo sapiens glutathione S-transferase alpha 4 (GSTA4), mRNA [NM_001512] | 1.2356591 | down |
| TMEM151A | Homo sapiens transmembrane protein 151A (TMEM151A), mRNA [NM_153266] | 1.3817376 | up |
|  | lincRNA:chr7:130589527-130590094 reverse strand | 1.4787537 | up |
| MXD1 | Homo sapiens MAX dimerization protein 1 (MXD1), transcript variant 1, mRNA [NM_002357] | 1.5511123 | up |
|  | lincRNA:chr14:96507172-96661947 reverse strand | 1.5977418 | up |
| IL8 | Homo sapiens interleukin 8 (IL8), mRNA [NM_000584] | 3.0696785 | up |
| GALNT3 | Homo sapiens UDP-N-acetyl-alpha-D-galactosamine:polypeptide N-acetylgalactosaminyltransferase 3 (GalNAc-T3) (GALNT3), mRNA [NM_004482] | 1.2123297 | up |
| SH3TC1 | Homo sapiens SH3 domain and tetratricopeptide repeats 1 (SH3TC1), mRNA [NM_018986] | 1.2280654 | up |
|  | coatomer protein complex, subunit gamma 2 [Source:HGNC Symbol;Acc:2237] [ENST00000445977] | 1.2321943 | down |
| RRAGD | Homo sapiens Ras-related GTP binding D (RRAGD), mRNA [NM_021244] | 1.7157729 | up |
| HRASLS2 | Homo sapiens HRAS-like suppressor 2 (HRASLS2), mRNA [NM_017878] | 3.0583081 | up |
| POLE | Homo sapiens polymerase (DNA directed), epsilon (POLE), mRNA [NM_006231] | 1.2058781 | down |
| IL11 | Homo sapiens interleukin 11 (IL11), mRNA [NM_000641] | 1.6658893 | up |
| ZNF697 | Homo sapiens zinc finger protein 697 (ZNF697), mRNA [NM_001080470] | 1.2520369 | up |
| C7orf34 | Homo sapiens chromosome 7 open reading frame 34 (C7orf34), mRNA [NM_178829] | 3.0392518 | up |
| ERBB4 | Homo sapiens v-erb-a erythroblastic leukemia viral oncogene homolog 4 (avian) (ERBB4), transcript variant JM-a/CVT-1, mRNA [NM_005235] | 1.3742843 | down |
|  | lincRNA:chr4:14744207-14889782 reverse strand | 1.4352809 | up |
| HYDIN | Homo sapiens hydrocephalus inducing homolog (mouse) (HYDIN), transcript variant 3, mRNA [NM_001198542] | 1.2673752 | down |
| MGC23284 | Homo sapiens uncharacterized LOC197187 (MGC23284), transcript variant 1, non-coding RNA [NR_024402] | 1.2164363 | down |
| LOC100287314 | Homo sapiens uncharacterized LOC100287314 (LOC100287314), non-coding RNA [NR_040245] | 1.2105988 | up |
| ARVCF | Homo sapiens armadillo repeat gene deleted in velocardiofacial syndrome (ARVCF), mRNA [NM_001670] | 1.3449169 | up |
|  | lincRNA:chr3:156894106-156971481 reverse strand | 3.0271018 | up |
|  | lincRNA:chr1:200331904-200332270 reverse strand | 1.2572587 | up |
| COX6B2 | Homo sapiens cytochrome c oxidase subunit VIb polypeptide 2 (testis) (COX6B2), mRNA [NM_144613] | 1.2282577 | up |
| C17orf74 | Homo sapiens chromosome 17 open reading frame 74 (C17orf74), mRNA [NM_175734] | 1.2241532 | up |
| SLC25A37 | Homo sapiens solute carrier family 25, member 37 (SLC25A37), nuclear gene encoding mitochondrial protein, mRNA [NM_016612] | 1.2937214 | up |
| SPTBN5 | Homo sapiens spectrin, beta, non-erythrocytic 5 (SPTBN5), mRNA [NM_016642] | 1.3244394 | up |
| FKBPL | Homo sapiens FK506 binding protein like (FKBPL), mRNA [NM_022110] | 1.2159349 | down |
| Q2I700 | Q2I700_9GAST (Q2I700) NADH dehydrogenase subunit 1, partial (7%) [THC2764903] | 1.2442567 | up |
|  |  | 3.0093234 | up |
| C13orf33 | Homo sapiens chromosome 13 open reading frame 33 (C13orf33), mRNA [NM_032849] | 1.5487534 | up |
| OR13A1 | Homo sapiens olfactory receptor, family 13, subfamily A, member 1 (OR13A1), mRNA [NM_001004297] | 1.2783372 | down |
| CECR5-AS1 | Homo sapiens CECR5 antisense RNA 1 (non-protein coding) (CECR5-AS1), transcript variant 2, non-coding RNA [NR_024482] | 1.2496668 | down |
| LOC100129617 | Homo sapiens uncharacterized LOC100129617 (LOC100129617), non-coding RNA [NR_045112] | 1.296973 | up |
| CSRNP1 | Homo sapiens cysteine-serine-rich nuclear protein 1 (CSRNP1), mRNA [NM_033027] | 1.3130648 | up |
| ABCD1 | ATP-binding cassette, sub-family D (ALD), member 1 [Source:HGNC Symbol;Acc:61] [ENST00000370129] | 1.2410175 | down |
| XLOC_002473 | BROAD Institute lincRNA (XLOC_002473), lincRNA [TCONS_00004564] | 1.2714614 | up |
| AKAP5 | Homo sapiens A kinase (PRKA) anchor protein 5 (AKAP5), mRNA [NM_004857] | 1.4642715 | up |
|  |  | 2.9692862 | up |
| ZSWIM4 | Homo sapiens zinc finger, SWIM-type containing 4 (ZSWIM4), mRNA [NM_023072] | 1.4232528 | up |
| RUNX1 | Homo sapiens runt-related transcription factor 1 (RUNX1), transcript variant 2, mRNA [NM_001001890] | 1.4139042 | up |
| C2orf62 | Homo sapiens chromosome 2 open reading frame 62 (C2orf62), mRNA [NM_198559] | 1.4483558 | down |
| ACTL8 | Homo sapiens actin-like 8 (ACTL8), mRNA [NM_030812] | 1.4141319 | down |
| CHRDL2 | Homo sapiens chordin-like 2 (CHRDL2), mRNA [NM_015424] | 1.4464962 | down |
| SLC35A1 | Homo sapiens solute carrier family 35 (CMP-sialic acid transporter), member A1 (SLC35A1), transcript variant 2, mRNA [NM_001168398] | 1.2050812 | down |
|  | lincRNA:chr8:55303672-55311247 forward strand | 1.6519567 | down |
| UNC5B | Homo sapiens unc-5 homolog B (C. elegans) (UNC5B), transcript variant 1, mRNA [NM_170744] | 2.9104006 | up |
|  | lincRNA:chr3:184997356-185017181 reverse strand | 1.2166102 | up |
| IFIT2 | Homo sapiens interferon-induced protein with tetratricopeptide repeats 2 (IFIT2), mRNA [NM_001547] | 2.8934648 | up |
| OASL | Homo sapiens 2'-5'-oligoadenylate synthetase-like (OASL), transcript variant 1, mRNA [NM_003733] | 2.8825095 | up |
| TBL1X | Homo sapiens transducin (beta)-like 1X-linked (TBL1X), transcript variant 1, mRNA [NM_005647] | 1.3356436 | down |
|  | ALU4_HUMAN (P39191) Alu subfamily SB2 sequence contamination warning entry, partial (18%) [THC2784818] | 1.2372833 | up |
|  | lincRNA:chr5:27050368-27625243 forward strand | 1.2044955 | down |
| C16orf59 | Homo sapiens chromosome 16 open reading frame 59 (C16orf59), mRNA [NM_025108] | 1.2451144 | down |
| IFIT3 | Homo sapiens interferon-induced protein with tetratricopeptide repeats 3 (IFIT3), transcript variant 1, mRNA [NM_001549] | 2.8781607 | up |
| FLJ37786 | PREDICTED: Homo sapiens hypothetical LOC642691 (FLJ37786), miscRNA [XR_108343] | 1.4018317 | up |
| ZAN | Homo sapiens zonadhesin (ZAN), transcript variant 6, mRNA [NM_173059] | 1.2186966 | up |
| UPP1 | uridine phosphorylase 1 [Source:HGNC Symbol;Acc:12576] [ENST00000457596] | 1.2010431 | up |
| CYR61 | Homo sapiens cysteine-rich, angiogenic inducer, 61 (CYR61), mRNA [NM_001554] | 1.4695663 | up |
| SLFNL1 | Homo sapiens schlafen-like 1 (SLFNL1), transcript variant 1, mRNA [NM_144990] | 2.8751254 | up |
| ATF3 | Homo sapiens activating transcription factor 3 (ATF3), transcript variant 4, mRNA [NM_001040619] | 2.8342135 | up |
| ATF3 | Homo sapiens activating transcription factor 3 (ATF3), transcript variant 1, mRNA [NM_001674] | 1.6301392 | up |
| HHLA1 | Homo sapiens HERV-H LTR-associating 1 (HHLA1), mRNA [NM_001145095] | 1.625335 | up |
| NOTUM | Homo sapiens notum pectinacetylesterase homolog (Drosophila) (NOTUM), mRNA [NM_178493] | 1.2833437 | up |
| DHRS2 | Homo sapiens dehydrogenase/reductase (SDR family) member 2 (DHRS2), transcript variant 1, mRNA [NM_182908] | 1.4379594 | up |
| LMO7 | Homo sapiens cDNA FLJ34733 fis, clone MESAN2006953, highly similar to LIM domain only 7 isoform c. [AK092052] | 1.2702253 | down |
| ZDHHC22 | Homo sapiens zinc finger, DHHC-type containing 22 (ZDHHC22), mRNA [NM_174976] | 1.4421666 | up |
| WTIP | Homo sapiens Wilms tumor 1 interacting protein (WTIP), mRNA [NM_001080436] | 1.5397294 | up |
| Q6P4E4 | Q6P4E4_HUMAN (Q6P4E4) RPL37A protein, partial (54%) [THC2593447] | 1.4156942 | up |
|  | lincRNA:chr19:19887392-19897442 forward strand | 1.5131979 | up |
| PCDH1 | Homo sapiens protocadherin 1 (PCDH1), transcript variant 1, mRNA [NM_002587] | 1.4939421 | up |
| FLJ39095 | Homo sapiens cDNA FLJ39095 fis, clone NT2RP7020379. [AK096414] | 1.3574022 | up |
|  | PREDICTED: Homo sapiens hypothetical LOC100506376 (LOC100506376), miscRNA [XR_132470] | 1.8557391 | up |
|  |  | 1.6720452 | up |
|  | lincRNA:chr8:28912381-28922959 forward strand | 1.2483243 | up |
| POTED | Homo sapiens POTE ankyrin domain family, member D (POTED), mRNA [NM_174981] | 1.4636544 | up |
| RAB3D | Homo sapiens RAB3D, member RAS oncogene family (RAB3D), mRNA [NM_004283] | 1.2008462 | down |
| LOC100499194 | Homo sapiens uncharacterized LOC100499194 (LOC100499194), non-coding RNA [NR_034130] | 1.4768586 | up |
| DYRK2 | Homo sapiens dual-specificity tyrosine-(Y)-phosphorylation regulated kinase 2 (DYRK2), transcript variant 2, mRNA [NM_006482] | 1.2411299 | up |
|  |  | 2.814143 | up |
| NEU1 | Homo sapiens sialidase 1 (lysosomal sialidase) (NEU1), mRNA [NM_000434] | 1.2214903 | up |
| ATF3 | Homo sapiens activating transcription factor 3 (ATF3), transcript variant 4, mRNA [NM_001040619] | 2.7962499 | up |
| DQX1 | Homo sapiens DEAQ box RNA-dependent ATPase 1 (DQX1), mRNA [NM_133637] | 1.4073889 | up |
| PRSS47 | PREDICTED: Homo sapiens protease, serine, 47 (PRSS47), miscRNA [XR_108978] | 1.6284714 | up |
| KRI1 | Homo sapiens KRI1 homolog (S. cerevisiae) (KRI1), mRNA [NM_023008] | 1.2547917 | down |
|  | fs15d03.y2 Human Lens cDNA (Normalized): fs Homo sapiens cDNA clone fs15d03 5', mRNA sequence [CD674797] | 1.3192841 | up |
|  | lincRNA:chr17:67547498-67549996 forward strand | 1.6073271 | down |
|  |  | 1.2014264 | down |
|  | Uncharacterized protein [Source:UniProtKB/TrEMBL;Acc:E7ES41] [ENST00000378417] | 1.3167683 | up |
| LOC100507547 | Homo sapiens uncharacterized LOC100507547 (LOC100507547), transcript variant 1, non-coding RNA [NR_037169] | 1.2213578 | down |
| FLJ45248 | Homo sapiens cDNA FLJ45248 fis, clone BRHIP2006819. [AK127183] | 1.3582904 | up |
| CFLAR | Homo sapiens CASP8 and FADD-like apoptosis regulator (CFLAR), transcript variant 3, mRNA [NM_001127184] | 1.2481065 | up |
| OR56B4 | Homo sapiens olfactory receptor, family 56, subfamily B, member 4 (OR56B4), mRNA [NM_001005181] | 1.4369365 | up |
| ATHL1 | Homo sapiens ATH1, acid trehalase-like 1 (yeast) (ATHL1), mRNA [NM_025092] | 1.5279068 | up |
|  |  | 1.3965845 | down |
| HIST1H2AJ | Homo sapiens histone cluster 1, H2aj (HIST1H2AJ), mRNA [NM_021066] | 1.226946 | down |
| DCAF6 | Homo sapiens DDB1 and CUL4 associated factor 6 (DCAF6), transcript variant 2, mRNA [NM_001017977] | 1.2451861 | down |
| RHBDL2 | Homo sapiens rhomboid, veinlet-like 2 (Drosophila) (RHBDL2), mRNA [NM_017821] | 1.2489058 | up |
| HIP1R | Homo sapiens huntingtin interacting protein 1 related (HIP1R), mRNA [NM_003959] | 1.229263 | up |
| HIST1H1B | Homo sapiens histone cluster 1, H1b (HIST1H1B), mRNA [NM_005322] | 1.5024763 | down |
| PDE7B | Homo sapiens phosphodiesterase 7B (PDE7B), mRNA [NM_018945] | 1.4170305 | down |
| CDCA7L | Homo sapiens cell division cycle associated 7-like (CDCA7L), transcript variant 1, mRNA [NM_018719] | 1.2731245 | down |
| SPTSSA | Homo sapiens serine palmitoyltransferase, small subunit A (SPTSSA), mRNA [NM_138288] | 1.375714 | down |
| LOC730651 | PREDICTED: Homo sapiens zinc finger protein 709-like (LOC730651), miscRNA [XR_132666] | 1.39935 | down |
|  | Homo sapiens clone DNA172120 micronovel (UNQ9353) mRNA, complete cds. [AY358253] | 1.2651787 | up |
|  | Homo sapiens mRNA; cDNA DKFZp686H0413 (from clone DKFZp686H0413). [AL832737] | 1.8901132 | up |
| IFI6 | Homo sapiens interferon, alpha-inducible protein 6 (IFI6), transcript variant 3, mRNA [NM_022873] | 2.73708 | up |
|  | lincRNA:chr1:59049237-59065662 reverse strand | 2.724613 | up |
| DNPEP | Homo sapiens aspartyl aminopeptidase (DNPEP), mRNA [NM_012100] | 1.353603 | down |
| HHIP | Homo sapiens hedgehog interacting protein (HHIP), mRNA [NM_022475] | 1.4850367 | up |
| MRGPRE | Homo sapiens MAS-related GPR, member E (MRGPRE), mRNA [NM_001039165] | 1.7252251 | up |
| CLCNKB | Homo sapiens chloride channel Kb (CLCNKB), transcript variant 1, mRNA [NM_000085] | 1.3467476 | up |
| ZDHHC11 | Homo sapiens zinc finger, DHHC-type containing 11 (ZDHHC11), mRNA [NM_024786] | 1.3116243 | up |
|  | lincRNA:chr10:97639610-97666960 forward strand | 1.3125988 | up |
| PRSS38 | Homo sapiens protease, serine, 38 (PRSS38), mRNA [NM_183062] | 1.4124804 | down |
| Q58MP6 | Q58MP6_9CAUD (Q58MP6) Dioxygenase, partial (8%) [THC2619587] | 1.7638953 | up |
| IPW | Homo sapiens imprinted in Prader-Willi syndrome (non-protein coding) (IPW), non-coding RNA [NR_023915] | 1.3934293 | down |
|  | histone cluster 1, H2ai [Source:HGNC Symbol;Acc:4725] [ENST00000358739] | 1.5728277 | down |
|  | lincRNA:chr12:57761837-57762303 reverse strand | 1.6655191 | down |
| ERRFI1 | Homo sapiens ERBB receptor feedback inhibitor 1 (ERRFI1), mRNA [NM_018948] | 1.2673681 | up |
|  | lincRNA:chr5:171237095-171263795 forward strand | 1.5637774 | up |
| IRAK2 | Homo sapiens interleukin-1 receptor-associated kinase 2 (IRAK2), mRNA [NM_001570] | 1.4448102 | up |
| RNF126 | Homo sapiens ring finger protein 126 (RNF126), mRNA [NM_194460] | 1.7216982 | up |
| IWS1 | IWS1 homolog (S. cerevisiae) [Source:HGNC Symbol;Acc:25467] [ENST00000409725] | 1.3224599 | down |
|  | lincRNA:chr12:13697208-13702308 reverse strand | 1.3208132 | down |
|  | lincRNA:chr7:20258275-20270775 forward strand | 1.3087184 | up |
| HIST1H3D | Homo sapiens histone cluster 1, H3d (HIST1H3D), mRNA [NM_003530] | 1.2874408 | down |
| GPNMB | Homo sapiens glycoprotein (transmembrane) nmb (GPNMB), transcript variant 1, mRNA [NM_001005340] | 1.3142484 | up |
| GRIN3B | Homo sapiens glutamate receptor, ionotropic, N-methyl-D-aspartate 3B (GRIN3B), mRNA [NM_138690] | 1.2864792 | up |
|  | hg18:lincRNA:chr2:239460050-239536125 forward strand | 1.2633665 | down |
| KCNK6 | Homo sapiens potassium channel, subfamily K, member 6 (KCNK6), mRNA [NM_004823] | 1.557463 | up |
| HIST1H3H | Homo sapiens histone cluster 1, H3h (HIST1H3H), mRNA [NM_003536] | 1.2246933 | down |
| RUNX1 | Homo sapiens runt-related transcription factor 1 (RUNX1), transcript variant 3, mRNA [NM_001122607] | 1.2883857 | up |
|  | lincRNA:chr11:70238327-70244052 reverse strand | 1.3267025 | down |
| AFF3 | AF4/FMR2 family, member 3 [Source:HGNC Symbol;Acc:6473] [ENST00000483600] | 1.6593612 | up |
| NCAPD2 | Homo sapiens non-SMC condensin I complex, subunit D2 (NCAPD2), mRNA [NM_014865] | 1.2729622 | down |
| RPGRIP1L | Homo sapiens RPGRIP1-like (RPGRIP1L), transcript variant 1, mRNA [NM_015272] | 1.2154806 | down |
| STIP1 | Homo sapiens stress-induced-phosphoprotein 1 (STIP1), mRNA [NM_006819] | 1.2009089 | down |
| PXN | Homo sapiens paxillin (PXN), transcript variant 1, mRNA [NM_002859] | 1.2346061 | up |
| PBK | Homo sapiens PDZ binding kinase (PBK), mRNA [NM_018492] | 1.2040272 | down |
|  | lincRNA:chr11:89926502-89932477 reverse strand | 1.275125 | down |
|  | lincRNA:chr1:142789202-142921002 reverse strand | 1.4946297 | up |
|  | Homo sapiens cDNA FLJ46531 fis, clone THYMU3036310. [AK128388] | 1.366689 | up |
|  | lincRNA:chrX:110792894-110849319 forward strand | 2.6964004 | up |
| SPANXB2 | Homo sapiens SPANX family, member B2 (SPANXB2), mRNA [NM_145664] | 1.305456 | up |
|  | lincRNA:chr6:56723191-56734391 reverse strand | 1.2493024 | down |
| FAM25A | Homo sapiens family with sequence similarity 25, member A (FAM25A), mRNA [NM_001146157] | 1.2924687 | up |
| LRRC32 | Homo sapiens leucine rich repeat containing 32 (LRRC32), transcript variant 1, mRNA [NM_005512] | 2.693087 | up |
| SPINK1 | Homo sapiens serine peptidase inhibitor, Kazal type 1 (SPINK1), mRNA [NM_003122] | 2.647552 | up |
|  |  | 1.2813969 | up |
| CD302 | Homo sapiens CD302 molecule (CD302), transcript variant 1, mRNA [NM_014880] | 1.2169218 | down |
| LOC100506459 | PREDICTED: Homo sapiens hypothetical LOC100506459 (LOC100506459), miscRNA [XR_110492] | 1.3124193 | up |
| PTPN13 | Homo sapiens protein tyrosine phosphatase, non-receptor type 13 (APO-1/CD95 (Fas)-associated phosphatase) (PTPN13), transcript variant 4, mRNA [NM_080685] | 1.2995559 | down |
| RELB | Homo sapiens v-rel reticuloendotheliosis viral oncogene homolog B (RELB), mRNA [NM_006509] | 2.638489 | up |
| GIT2 | Homo sapiens G protein-coupled receptor kinase interacting ArfGAP 2 (GIT2), transcript variant 4, mRNA [NM_139201] | 1.2019196 | down |
| FAT4 | Homo sapiens FAT tumor suppressor homolog 4 (Drosophila) (FAT4), mRNA [NM_024582] | 1.3441762 | down |
|  |  | 1.3107128 | up |
| C20orf195 | Homo sapiens chromosome 20 open reading frame 195 (C20orf195), mRNA [NM_024059] | 1.517268 | up |
| HMGA2 | Homo sapiens high mobility group AT-hook 2 (HMGA2), transcript variant 2, mRNA [NM_003484] | 1.6390476 | up |
|  | lincRNA:chrX:56807400-56832200 reverse strand | 1.2875692 | down |
|  | lincRNA:chr9:2739400-2746875 forward strand | 1.4848084 | up |
| HIST1H2AC | Homo sapiens histone cluster 1, H2ac (HIST1H2AC), mRNA [NM_003512] | 1.2056857 | down |
|  | lincRNA:chr14:71020897-71044497 reverse strand | 1.7155728 | down |
| ZFYVE28 | Homo sapiens zinc finger, FYVE domain containing 28 (ZFYVE28), transcript variant 4, mRNA [NM_001172657] | 1.3418573 | up |
| MAP2K3 | Homo sapiens mitogen-activated protein kinase kinase 3 (MAP2K3), transcript variant B, mRNA [NM_145109] | 1.206935 | up |
| LOC440297 | Homo sapiens chondroitin sulfate proteoglycan 4 pseudogene (LOC440297), non-coding RNA [NR_033579] | 1.4845718 | up |
|  |  | 2.632037 | up |
| RPL10 | Homo sapiens ribosomal protein L10 (RPL10), transcript variant 1, mRNA [NM_006013] | 1.4532723 | up |
| LOC440896 | Homo sapiens uncharacterized LOC440896 (LOC440896), non-coding RNA [NR_015361] | 1.2974554 | down |
| LOC100132363 | Homo sapiens cDNA FLJ39193 fis, clone OCBBF2004866. [AK096512] | 1.3822182 | up |
| GPR153 | Homo sapiens G protein-coupled receptor 153 (GPR153), mRNA [NM_207370] | 1.4205652 | up |
| MAFF | Homo sapiens v-maf musculoaponeurotic fibrosarcoma oncogene homolog F (avian) (MAFF), transcript variant 1, mRNA [NM_012323] | 2.6083841 | up |
| PIK3C2B | phosphoinositide-3-kinase, class 2, beta polypeptide [Source:HGNC Symbol;Acc:8972] [ENST00000367184] | 2.6069026 | up |
| CLDN4 | Homo sapiens claudin 4 (CLDN4), mRNA [NM_001305] | 1.3686265 | up |
| NCAPD2 | Homo sapiens non-SMC condensin I complex, subunit D2 (NCAPD2), mRNA [NM_014865] | 1.2316759 | down |
| BRI3 | Homo sapiens brain protein I3 (BRI3), transcript variant 1, mRNA [NM_015379] | 1.2103925 | up |
| RPL28 | Homo sapiens ribosomal protein L28 (RPL28), transcript variant 1, mRNA [NM_001136134] | 1.2145764 | down |
|  | lincRNA:chr4:53578995-53580281 reverse strand | 1.3140239 | down |
| LOC148413 | Homo sapiens uncharacterized LOC148413 (LOC148413), non-coding RNA [NR_015434] | 1.3097544 | down |
| ELOVL7 | Homo sapiens ELOVL fatty acid elongase 7 (ELOVL7), transcript variant 1, mRNA [NM_024930] | 1.4963541 | up |
| SNHG13 | Homo sapiens small nucleolar RNA host gene 13 (non-protein coding) (SNHG13), non-coding RNA [NR_024031] | 1.3552057 | down |
| SLC12A6 | Homo sapiens solute carrier family 12 (potassium/chloride transporters), member 6 (SLC12A6), transcript variant 5, mRNA [NM_001042496] | 1.2251586 | up |
| TMEM203 | Homo sapiens transmembrane protein 203 (TMEM203), mRNA [NM_053045] | 1.2153485 | down |
| AEN | Homo sapiens apoptosis enhancing nuclease (AEN), mRNA [NM_022767] | 1.2388831 | up |
| MANBA | Homo sapiens mannosidase, beta A, lysosomal (MANBA), mRNA [NM_005908] | 1.3713498 | up |
|  | lincRNA:chr5:25385943-25445890 forward strand | 1.7032591 | up |
| GUSBP10 | Homo sapiens glucuronidase, beta pseudogene 10 (GUSBP10), non-coding RNA [NR_030766] | 1.26943 | up |
| HIST1H1A | Homo sapiens histone cluster 1, H1a (HIST1H1A), mRNA [NM_005325] | 1.2377547 | down |
| ITGA4 | Homo sapiens integrin, alpha 4 (antigen CD49D, alpha 4 subunit of VLA-4 receptor) (ITGA4), mRNA [NM_000885] | 1.3516318 | up |
| LOC100128198 | Homo sapiens cDNA FLJ43053 fis, clone BRTHA3006856. [AK125043] | 1.5764918 | up |
| TMEM201 | Homo sapiens transmembrane protein 201 (TMEM201), transcript variant 1, mRNA [NM_001130924] | 1.3464803 | up |
| ZBED3 | Homo sapiens zinc finger, BED-type containing 3 (ZBED3), mRNA [NM_032367] | 1.3055636 | down |
| WDR6 | Homo sapiens WD repeat domain 6 (WDR6), mRNA [NM_018031] | 1.2060963 | down |
| NEK7 | Homo sapiens NIMA (never in mitosis gene a)-related kinase 7 (NEK7), mRNA [NM_133494] | 1.2122467 | up |
| BCAT2 | Homo sapiens branched chain amino-acid transaminase 2, mitochondrial (BCAT2), nuclear gene encoding mitochondrial protein, transcript variant a, mRNA [NM_001190] | 1.2741852 | down |
| GAB2 | Homo sapiens GRB2-associated binding protein 2 (GAB2), transcript variant 2, mRNA [NM_012296] | 1.2883644 | up |
|  | lincRNA:chr7:157072599-157073109 reverse strand | 1.3139365 | down |
| PROKR2 | Homo sapiens prokineticin receptor 2 (PROKR2), mRNA [NM_144773] | 2.5369892 | up |
| WDR27 | Homo sapiens WD repeat domain 27 (WDR27), transcript variant 1, mRNA [NM_182552] | 1.3130717 | down |
|  | lincRNA:chr6:114722782-114743483 forward strand | 1.2693179 | up |
| IL31RA | Homo sapiens interleukin 31 receptor A (IL31RA), transcript variant 4, mRNA [NM_001242638] | 1.349534 | up |
| RGR | retinal G protein coupled receptor [Source:HGNC Symbol;Acc:9990] [ENST00000372092] | 1.2949975 | up |
| OR10A5 | Homo sapiens olfactory receptor, family 10, subfamily A, member 5 (OR10A5), mRNA [NM_178168] | 1.349655 | up |
| UCA1 | Homo sapiens urothelial cancer associated 1 (non-protein coding) (UCA1), non-coding RNA [NR_015379] | 1.8481379 | up |
| C16orf73 | Homo sapiens chromosome 16 open reading frame 73 (C16orf73), transcript variant 2, mRNA [NM_152764] | 1.3261672 | up |
| ARSG | arylsulfatase G [Source:HGNC Symbol;Acc:24102] [ENST00000448504] | 1.4917705 | down |
| RBM23 | Homo sapiens RNA binding motif protein 23 (RBM23), transcript variant 1, mRNA [NM_001077351] | 1.2475158 | down |
|  |  | 1.6030334 | down |
| P39192 | ALU5_HUMAN (P39192) Alu subfamily SC sequence contamination warning entry, partial (4%) [THC2545150] | 1.2537533 | up |
| PSG8 | Homo sapiens pregnancy specific beta-1-glycoprotein 8 (PSG8), transcript variant 1, mRNA [NM_182707] | 1.344094 | up |
|  |  | 1.4812098 | down |
| CTSL1P2 | Homo sapiens cathepsin L1 pseudogene 2 (CTSL1P2), non-coding RNA [NR_033407] | 2.5042844 | up |
| BRWD1 | Homo sapiens bromodomain and WD repeat domain containing 1 (BRWD1), transcript variant 1, mRNA [NM_018963] | 1.4178463 | down |
| SEC22C | Homo sapiens SEC22 vesicle trafficking protein homolog C (S. cerevisiae) (SEC22C), transcript variant 4, mRNA [NM_001201584] | 1.2304544 | down |
| TBXA2R | Homo sapiens thromboxane A2 receptor (TBXA2R), transcript variant a, mRNA [NM_001060] | 1.2001752 | up |
| CCNB2 | Homo sapiens cyclin B2 (CCNB2), mRNA [NM_004701] | 1.234615 | down |
| NAT6 | Homo sapiens N-acetyltransferase 6 (GCN5-related) (NAT6), transcript variant 1, mRNA [NM_012191] | 1.229594 | down |
|  |  | 1.2063527 | down |
|  | lincRNA:chr15:64753847-64771422 reverse strand | 1.4081227 | down |
|  | acyl-CoA synthetase long-chain family member 6 [Source:HGNC Symbol;Acc:16496] [ENST00000413683] | 1.329001 | up |
| CEP72 | Homo sapiens centrosomal protein 72kDa (CEP72), mRNA [NM_018140] | 1.227093 | down |
| RIOK3 | Homo sapiens RIO kinase 3 (yeast) (RIOK3), mRNA [NM_003831] | 1.2329961 | up |
| FOXO4 | Homo sapiens forkhead box O4 (FOXO4), transcript variant 1, mRNA [NM_005938] | 1.3116496 | down |
| NT5DC1 | Homo sapiens 5'-nucleotidase domain containing 1 (NT5DC1), mRNA [NM_152729] | 1.2793784 | down |
|  | lincRNA:chr2:70276172-70277331 reverse strand | 1.2597713 | down |
| PDE8B | Homo sapiens phosphodiesterase 8B (PDE8B), transcript variant 1, mRNA [NM_003719] | 1.3824381 | down |
| SLC2A5 | Homo sapiens solute carrier family 2 (facilitated glucose/fructose transporter), member 5 (SLC2A5), transcript variant 1, mRNA [NM_003039] | 1.3539062 | up |
| AKR1B10 | Homo sapiens aldo-keto reductase family 1, member B10 (aldose reductase) (AKR1B10), mRNA [NM_020299] | 1.2884351 | down |
|  | lincRNA:chr10:118588285-118590480 reverse strand | 1.2493325 | down |
| FUT10 | fucosyltransferase 10 (alpha (1,3) fucosyltransferase) [Source:HGNC Symbol;Acc:19234] [ENST00000335589] | 1.2741796 | down |
| NUAK1 | Homo sapiens NUAK family, SNF1-like kinase, 1 (NUAK1), mRNA [NM_014840] | 1.2287512 | up |
| LOC375196 | Homo sapiens uncharacterized LOC375196 (LOC375196), non-coding RNA [NR_028386] | 1.2826463 | down |
|  | lincRNA:chr20:34628511-34640236 forward strand | 1.4713223 | up |
| CBFA2T3 | Homo sapiens core-binding factor, runt domain, alpha subunit 2; translocated to, 3 (CBFA2T3), transcript variant 1, mRNA [NM_005187] | 1.4117534 | up |
| TMEM45A | Homo sapiens transmembrane protein 45A (TMEM45A), mRNA [NM_018004] | 1.2738895 | up |
| HPDL | Homo sapiens 4-hydroxyphenylpyruvate dioxygenase-like (HPDL), mRNA [NM_032756] | 1.3160173 | down |
|  | lincRNA:chr20:32861439-32866564 forward strand | 1.2661009 | up |
| C10orf53 | Homo sapiens chromosome 10 open reading frame 53 (C10orf53), transcript variant 1, mRNA [NM_182554] | 2.502094 | up |
| IGFL2 | Homo sapiens IGF-like family member 2 (IGFL2), transcript variant 1, mRNA [NM_001002915] | 1.8969827 | up |
| LOC283454 | Homo sapiens cDNA FLJ37411 fis, clone BRAMY2028682. [AK094730] | 2.4867368 | up |
| PLA2G1B | Homo sapiens phospholipase A2, group IB (pancreas) (PLA2G1B), mRNA [NM_000928] | 1.4759729 | up |
| TDRG1 | Homo sapiens testis development related protein 1 (TDRG1), non-coding RNA [NR_024015] | 2.484406 | up |
| CD177 | Homo sapiens CD177 molecule (CD177), mRNA [NM_020406] | 1.313717 | up |
| ZFYVE1 | Homo sapiens zinc finger, FYVE domain containing 1 (ZFYVE1), transcript variant 1, mRNA [NM_021260] | 1.2019427 | up |
|  | COEA1_HUMAN (Q05707) Collagen alpha-1(XIV) chain precursor (Undulin), partial (27%) [THC2511093] | 1.2474643 | down |
| SCARA5 | Homo sapiens scavenger receptor class A, member 5 (putative) (SCARA5), mRNA [NM_173833] | 1.367512 | down |
| CYP51A1 | Homo sapiens cytochrome P450, family 51, subfamily A, polypeptide 1 (CYP51A1), transcript variant 1, mRNA [NM_000786] | 1.233057 | up |
|  | lincRNA:chr6:38124747-38131872 reverse strand | 1.7086806 | up |
| VKORC1 | Homo sapiens cDNA FLJ43630 fis, clone SPLEN2030479. [AK125618] | 1.2132305 | down |
| RUSC2 | Homo sapiens RUN and SH3 domain containing 2 (RUSC2), mRNA [NM_014806] | 1.249127 | up |
| KCNK1 | Homo sapiens potassium channel, subfamily K, member 1 (KCNK1), mRNA [NM_002245] | 1.2647332 | up |
| LOC100506190 |  | 1.2493347 | down |
|  | lincRNA:chr1:201482952-201545677 reverse strand | 1.3245541 | up |
| GRIK2 | Homo sapiens glutamate receptor, ionotropic, kainate 2 (GRIK2), transcript variant 2, mRNA [NM_175768] | 1.360235 | down |
| CBR1 | Homo sapiens cDNA, FLJ18261. [AK311219] | 1.7516433 | up |
| PLK3 | Homo sapiens polo-like kinase 3 (PLK3), mRNA [NM_004073] | 1.3428193 | up |
|  | lincRNA:chr16:11581190-11588611 forward strand | 1.3814647 | up |
| ZNF114 | Homo sapiens zinc finger protein 114 (ZNF114), mRNA [NM_153608] | 2.4656637 | up |
|  | lincRNA:chr8:90615284-90628709 forward strand | 1.2301613 | up |
| CDRT1 | Homo sapiens CMT1A duplicated region transcript 1 (CDRT1), mRNA [NM_006382] | 1.297674 | up |
| DHRS12 | Homo sapiens dehydrogenase/reductase (SDR family) member 12 (DHRS12), transcript variant 1, mRNA [NM_001031719] | 1.220581 | down |
| SLC25A10 | Homo sapiens solute carrier family 25 (mitochondrial carrier; dicarboxylate transporter), member 10 (SLC25A10), nuclear gene encoding mitochondrial protein, mRNA [NM_012140] | 1.2561573 | down |
| CDC14C | Homo sapiens CDC14 cell division cycle 14 homolog C (S. cerevisiae) (CDC14C), non-coding RNA [NR_003595] | 1.2273972 | up |
| SGK110 | Homo sapiens putative uncharacterized serine/threonine-protein kinase SgK110-like (SGK110), mRNA [NM_001199824] | 1.4323577 | up |
|  | lincRNA:chr6:56709666-56716191 forward strand | 1.2765555 | up |
| SAMD9L | Homo sapiens sterile alpha motif domain containing 9-like (SAMD9L), mRNA [NM_152703] | 1.9867636 | up |
| PTK6 | Homo sapiens PTK6 protein tyrosine kinase 6 (PTK6), mRNA [NM_005975] | 1.244375 | up |
| FAM91A1 | Homo sapiens family with sequence similarity 91, member A1 (FAM91A1), mRNA [NM_144963] | 1.2379761 | up |
| PANX1 | Homo sapiens pannexin 1 (PANX1), mRNA [NM_015368] | 1.3833374 | up |
| MEF2C | Homo sapiens myocyte enhancer factor 2C (MEF2C), transcript variant 1, mRNA [NM_002397] | 1.4790523 | down |
| QRICH2 | Homo sapiens glutamine rich 2 (QRICH2), mRNA [NM_032134] | 1.8716854 | up |
| IFT80 | Homo sapiens intraflagellar transport 80 homolog (Chlamydomonas) (IFT80), transcript variant 1, mRNA [NM_020800] | 1.2127186 | down |
| LOC100288292 | PREDICTED: Homo sapiens putative uncharacterized protein FLJ44672-like (LOC100288292), miscRNA [XR_132538] | 1.3208275 | up |
| RNF19B | Homo sapiens ring finger protein 19B (RNF19B), transcript variant 1, mRNA [NM_153341] | 1.3685186 | up |
| ROR1 | receptor tyrosine kinase-like orphan receptor 1 [Source:HGNC Symbol;Acc:10256] [ENST00000371079] | 1.3042331 | up |
| KCTD12 | Homo sapiens potassium channel tetramerisation domain containing 12 (KCTD12), mRNA [NM_138444] | 1.3220999 | down |
| LOC100499194 | Homo sapiens uncharacterized LOC100499194 (LOC100499194), non-coding RNA [NR_034130] | 1.2619148 | up |
| RAB3A | Homo sapiens RAB3A, member RAS oncogene family (RAB3A), mRNA [NM_002866] | 1.4161397 | down |
|  | lincRNA:chr21:36128280-36139780 forward strand | 2.4523501 | up |
| ZNF267 | Homo sapiens zinc finger protein 267 (ZNF267), transcript variant 498723, mRNA [NM_003414] | 1.2141006 | up |
| GLB1L3 | galactosidase, beta 1-like 3 [Source:HGNC Symbol;Acc:25147] [ENST00000389887] | 2.450822 | up |
| TMEM125 | Homo sapiens transmembrane protein 125 (TMEM125), mRNA [NM_144626] | 1.3926344 | up |
| DDAH1 | Homo sapiens dimethylarginine dimethylaminohydrolase 1 (DDAH1), transcript variant 1, mRNA [NM_012137] | 1.2624111 | up |
