# Supplementary material for: White spot syndrome viral protein VP9 alters the cellular higher‐order chromatin structure
Source: FASEB Bioadv. 2020 Mar 17;2(4):264–79. doi: 10.1096/fba.2019-00086 (PMC7133739; doi:10.1096/fba.2019-00086)
Supplement: Supplementary file 10 [file FBA2-2-264-s010.pdf]

Supplemental information

Fig S1. VP9 does not directly affect the extension of naked dsDNA. (A) The extension of a single molecule of naked dsDNA at each of the constant forces (0.17, 0.46, 1.19, and 3.2 pN) was tested with four different concentrations of VP9 (0, 0.01, 0.1 and 1 mg/ml). The extension of dsDNA remained almost the same at all of the forces tested, irrespective of VP9 concentration. (B) The elastic response of DNA was tested by applying different forces (from 3.2 pN, down to 0.17 pN, and back to 3.2 pN) over a period of 600 seconds. Varying the concentration of VP9 did not affect the elastic response of DNA. DNA manipulation experiments were carried out in a buffer containing 10 mM Tris-HCl, pH 7.6, 150 mM NaCl, 1 mM EDTA at 23°C.

Fig S2. Stepwise folding and unfolding of nucleosomes. (A) 3.6 μM histone octamers were pre-incubated with 10 uM of histone chaperone NAP-1 for 2h in a buffer containing pH 7.6, 150 mM NaCl, 10 mM Tris, 1 mM EDTA. Stepwise folding was observed under the constant force of 5 pN; (B) Stepwise unfolding of DNA under a constant force of 15 pN; (C) An enlarged part of Figure B demonstrates the step size is 50 nm, which corresponds to a size of 146 bp DNA.

Fig S3. VP9 impedes DNA folding around histone octamers. (A) Dynamic DNA folding in a 100 nM histone octamer solution. Each color denotes a constant force as shown in panel E. The DNA extension was recorded at each constant force for 200 sec. (B) and (C) Dynamics of DNA folding when incubated in premixed solutions of 100 nM histone octamers with 10 μM and 100 μM of VP9, respectively. Incubation with VP9 allows the DNA to be stretched longer under a constant force of 6.13 pN (red arrows) in a concentration dependent manner, demonstrating that

VP9 expression impedes DNA folding. (D) Dynamic DNA folding in a premixed 100 nM of histone octamers and 100 μM BSA. All reaction buffers contain 150 mM NaCl, 10 mM Tris and 1 mM EDTA at pH 7.6.

Fig S4. Transfection efficiency measured with pXJ40-mCherry. HeLa cells were transfected with 0.4 μg of pXJ40-VP9 (non-fluorescent) and 0.1 μg of pXJ40-mCherry plasmids. Expression of mCherry was used as an indicator to optimize transfection conditions to achieve at least 60% transfection efficiency. The two representative images of VP9 transfection (Region 1 and 2) were taken at 24 h post-transfection. Left column: bright field; Right column: mCherry

Fig S5. Detection of apoptosis in VP9-transfected cells. HeLa cells were transfected with 0.1 μg of pXJ40-mCherry (mCherry only) and 0.4 μg of pXJ40-VP9 (mCherry-VP9). After 24 h post transfection, cells were collected, stained with annexin V and DAPI (ThermoFisher Scientific) and analyzed by flow cytometry (BD LSRFortessa). The percentage in each square represents the proportion of cells undergoing: Q1=necrosis, Q2=late apoptosis/necrosis; Q3=viable cells; Q4=early apoptosis. No significant difference in cell death was observed between cells transfected with the empty vector or VP9. The data in A and B represent two independent experimental results.

Fig S6. Color-coded anisotropy maps. (A) Representative color-coded H3-EGFP anisotropy images of control and VP9-expressing cell nuclei. Red represents high anisotropy, i.e. compact chromatin, while blue represents low anisotropy, i.e. decompacted chromatin. (B) Homo-FRET does not contribute to anisotropy. Representative images of intensity (top panel) and anisotropy

(bottom panel) from VP9-transfected H3-EGFP cell nuclei show that anisotropy does not change before and after photo-bleaching. White arrows indicate the bleached region.

Fig S7. Salt fractionation of nuclear proteins. Nuclei were isolated from control- and VP9-transfected (24 post-transfection) cells, and aliquoted into 7 tubes. One tube that did not undergo extraction was used as a control for total protein. The other 6 aliquots were extracted with buffers containing different concentrations of NaCl. (A) SDS–polyacrylamide gel analysis (SDS-PAGE) of proteins in salt fractions. The proportion of soluble histone factions was elevated following extraction with 0.6 M NaCl (red square) in the presence of VP9 expression (bottom panel) as compared to the control group (top panel); (B) Overlay of quantitative analysis of the relative intensity of soluble histone fractions at different salt concentrations for control group (top panel) and VP9-transfected group (bottom panel).

Fig S8. VP9 imposes gene expression changes in HeLa cells. (A) Gene Ontology (GO) analysis of 400 differentially regulated genes upon VP9 expression. The pie chart shows significantly ($P < 0.05$) altered gene clusters. DAVID bioinformatics resources were used for this analysis. Note: Phosphorylation, proteolysis and regulation of protein localization are included in protein functions; (B) Fold changes of selected genes from major functional clusters: including genes involved in DNA / RNA metabolism, the apoptotic signal pathway, nucleosome organization, and control of cell cycle.
